# A 1-phytase type III effector interferes with plant hormone signaling

**DOI:** 10.1038/s41467-017-02195-8

**Published:** 2017-12-18

**Authors:** Doreen Blüher, Debabrata Laha, Sabine Thieme, Alexandre Hofer, Lennart Eschen-Lippold, Antonia Masch, Gerd Balcke, Igor Pavlovic, Oliver Nagel, Antje Schonsky, Rahel Hinkelmann, Jakob Wörner, Nargis Parvin, Ralf Greiner, Stefan Weber, Alain Tissier, Mike Schutkowski, Justin Lee, Henning Jessen, Gabriel Schaaf, Ulla Bonas

**Affiliations:** 1 Institute for Biology, Department of Genetics, Martin-Luther University Halle-Wittenberg, Weinbergweg 10, 06120 Halle (Saale), Germany; 2 Institute of Crop Science and Resource Conservation, Department of Plant Nutrition, University of Bonn, Karlrobert-Kreiten-Strasse 13, 53115 Bonn, Germany; 3 Center for Plant Molecular Biology, Department of Plant Physiology, Eberhard Karls University Tübingen, Auf der Morgenstelle 32, 72076 Tübingen, Germany; 4 Department of Chemistry, University of Zurich, Winterthurerstrasse 190, 8057 Zurich, Switzerland; 5 Department of Stress and Developmental Biology, Leibniz Institute of Plant Biochemistry, Weinberg 3, 06120 Halle (Saale), Germany; 6 Institute for Biochemistry and Biotechnology, Department of Enzymology, Martin-Luther University Halle-Wittenberg, Kurt-Mothes-Strasse 3, 06120 Halle (Saale), Germany; 7 Department of Cell and Metabolic Biology, Leibniz Institute of Plant Biochemistry, Weinberg 3, 06120 Halle (Saale), Germany; 8 Institute of Organic Chemistry, Albert-Ludwigs University Freiburg, Albertstrasse 21, 79104 Freiburg, Germany; 9 Institute of Physical Chemistry, Albert-Ludwigs University Freiburg, Albertstrasse 21, 79104 Freiburg, Germany; 10 Department of Food Technology and Bioprocess Engineering, Max-Rubner-Institut, Federal Research Institute of Nutrition and Food, Haid-und-Neu-Straße 9, 76131 Karlsruhe, Germany

## Abstract

Most Gram-negative phytopathogenic bacteria inject type III effector (T3E) proteins into plant cells to manipulate signaling pathways to the pathogen’s benefit. In resistant plants, specialized immune receptors recognize single T3Es or their biochemical activities, thus halting pathogen ingress. However, molecular function and mode of recognition for most T3Es remains elusive. Here, we show that the *Xanthomonas* T3E XopH possesses phytase activity, i.e., dephosphorylates phytate (*myo*-inositol-*hexakis*phosphate, InsP_6_), the major phosphate storage compound in plants, which is also involved in pathogen defense. A combination of biochemical approaches, including a new NMR-based method to discriminate inositol polyphosphate enantiomers, identifies XopH as a naturally occurring 1-phytase that dephosphorylates InsP_6_ at C1. Infection of *Nicotiana benthamiana* and pepper by *Xanthomonas* results in a XopH-dependent conversion of InsP_6_ to InsP_5._ 1-phytase activity is required for XopH-mediated immunity of plants carrying the *Bs7* resistance gene, and for induction of jasmonate- and ethylene-responsive genes in *N. benthamiana*.

## Abstract (web summary)

Plant pathogens translocate type III effector (T3E) proteins that may be recognized by plants to trigger immunity. Here, the authors show that the *Xanthomonas* T3E XopH possesses a novel 1-phytase activity that is required for XopH-mediated immunity of plants carrying the *Bs7* resistance gene.

## Introduction

**Fig. 1:**
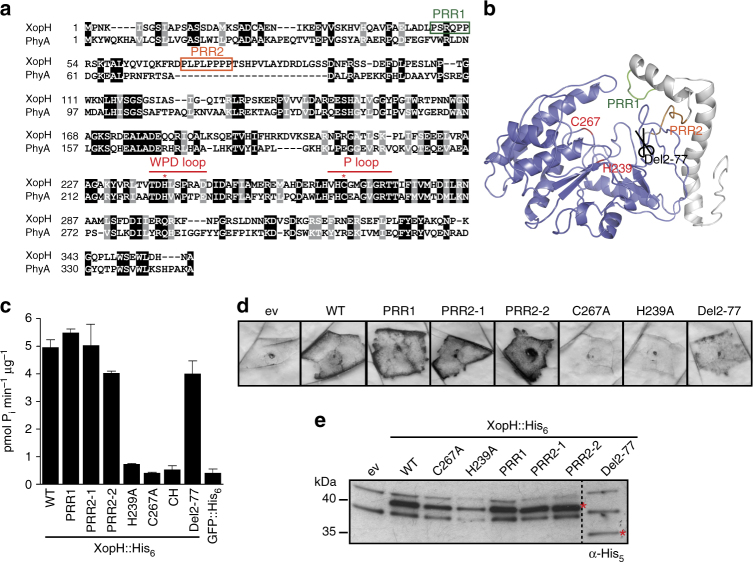
XopH possesses phytate-degrading activity that is required for HR induction. **a** Amino acid (aa) sequence alignment of XopH with the *Selenomonas ruminantium* phytase generated with T-Coffee^66^. Identical and similar aa are shaded black and gray, respectively, using Boxshade^67^. Dashes indicate gaps. Catalytic residues in the WPD and P loops are marked by asterisks. Proline-rich regions (PRRs) are boxed. **b** XopH protein structure modeled after the *S. ruminantium* phytase crystal structure (pdb 1U24) using Phyre2^14^, visualized by PyMol^68^. Blue, phytase domain; gray, N-terminal domain (aa 1–77). **c** InsP_6_ dephosphorylation by XopH (WT) and mutants, respectively. PRR1, P48,52,53A; PRR2-1, P69,71A; PRR2-2, P73,74,75,76A; CH, H239A/C267A; Del2-77, deletion of aa 2–77. GFP served as negative control. Values are means of two technical replicates. Error bars indicate s.d. The experiment was performed twice with similar results, using two independent protein preparations each. **d** HR induction in pepper ECW-70R (*Bs7*) leaves after *Agrobacterium-*mediated expression of XopH and mutant variants. Two days post inoculation (dpi), leaves were bleached in ethanol for better visualization of the HR. **e** Protein expression, two dpi, in the same plants analyzed in **d**. Immunoblot signals at expected sizes are marked by asterisks. The experiment was repeated twice with similar results

Gram-negative phytopathogenic xanthomonads infect a broad range of plant species causing substantial crop yield losses. Pathogenicity depends in most cases on a conserved type III secretion (T3S) system that translocates effector proteins directly into the plant cell cytosol^1^. The pepper and tomato pathogen *Xanthomonas campestris* pv. *vesicatoria* (*Xcv*, also termed *X. euvesicatoria* ^2^) encodes more than 30 type III effector (T3E) proteins, designated Xops (*Xanthomonas* outer proteins), whose collective action in host cells results in bacterial spot disease^3,4^. In resistant plant cultivars, single effectors are recognized by specific immune receptors^1^ often inducing the hypersensitive response (HR), a rapid, local programmed cell death at the infection site which restricts pathogen ingress^5^. While the molecular functions of most T3Es from *Xanthomonas* are elusive, members of the large family of transcription activator-like (TAL) effectors act as transcription factors in the plant cell^6^. Other T3Es display enzymatic activities such as the E3 ubiquitin ligase XopL^7^ or AvrBsT, a member of the YopJ/AvrRxv family of acetyltransferases^8^. XopH (also designated AvrBs1.1^9^) possesses typical features of dual-specific protein phosphatases, i.e., conserved amino acid residues in the active site (P loop) and the WPD loop involved in catalysis^10^ (Fig. 1a). Indeed, XopH dephosphorylates the generic phosphatase substrate pNPP (*p*-nitrophenyl phosphate) although its activity is weak^9^. Mutation of the P loop compromises both protein phosphatase activity and the XopH-dependent HR induction in resistant pepper plants^9^.

In this study, we reveal that XopH dephosphorylates *myo*-inositol-*hexakis*phosphate (phytate, InsP_6_) in vitro and in vivo. We provide evidence that XopH’s phytase activity is stereoselective for position C1, is required for XopH-dependent HR induction in plants carrying the *Bs7* resistance gene and induces the upregulation of hormone-responsive genes.

## Results

### XopH has weak protein phosphatase activity

Using optimized buffer conditions (Supplementary Fig. 1a), we determined essential amino acid residues for XopH-mediated phosphatase activity on pNPP: H239 in the WPD loop and C267 in the active site (Supplementary Fig. 1b). Similar results were obtained with the phosphotyrosine-containing peptide pTyr2, the best substrate out of six tested commercial phosphopeptides (Supplementary Fig. 1c, d). The XopH N-terminal region harbors two proline-rich regions (PRRs), putative peptide/protein interaction sites^11^ that might be involved in substrate recognition (Fig. 1a). Mutations in both PRR motifs compromised catalytic activity albeit less in case of the PRR1 motif. Deletion of the first 77 amino acid residues led to a complete loss of protein phosphatase activity (Supplementary Fig. 1d). To determine XopH substrate specificity, high-density peptide microarrays comprising more than 6000 pTyr peptides were incubated with XopH and the catalytically inactive C267A variant, respectively (for details see “Methods” section). The top 72 XopH substrates showed >70% cleavage by WT XopH and were compared to the negative sample set represented by all peptides displayed on the array. The resulting two-sample logo is shown in Supplementary Fig. 1e. Next, kinetic constants of XopH protein phosphatase activity were determined using three different phosphopeptides and optimized conditions (Supplementary Fig. 1f) in a discontinuous HPLC (high-performance liquid chromatography)-based assay. The randomly selected non-substrate from the microarray experiment was not dephosphorylated by XopH. By contrast, *K*_M_ values revealed similar affinities for the positive control peptide pTyr2 and the top substrate identified in the peptide microarray (“pTyr-chip”), while *k*_cat_ values indicated a threefold higher enzyme efficiency of XopH against “pTyr-chip” (Supplementary Fig. 1g and Table 1). Nonetheless, turnover rates were low compared to known protein tyrosine phosphatases^12,13^ raising the possibility that protein dephosphorylation does not represent XopH´s primary activity.

**Table 1:** Catalytic constants of tyrosine phosphatase and phytase activity of XopH^a^

Substrate	Additive	*V*_max_ (µmol L^−1^ min^−1^)	*K*_M_ (µM)	*k*_cat_ (s^−1^)	*k*_cat_/*K*_M_ (M^−1^ · s^−1^)	*R* ^2^
pTyr2	10 mM NaCl, 1 mM MgCl_2_	0.63 ± 0.16	25.7 ± 12.2	0.009	3.50 × 10^2^	0.89
pTyr-chip	10 mM NaCl, 1 mM MgCl_2_	0.21 ± 0.12	29.7 ± 3.0	0.028	9.42 × 10^2^	0.99
		**(p mol µg** ^**−1**^ **min** ^**−1**^ **)**				
InsP_6_	100 mM NaCl, 1 mM MgCl_2_	40.0 ± 5.1	72.9 ± 13.7	19.4	2.66 × 10^5^	0.99
InsP_6_	10 mM NaCl	110.6 ± 9.5	138.3 ± 15.0	45.3	3.28 × 10^5^	0.99

^a^Activity was measured in 50 mM HEPES (pH 7.0) under reducing conditions as described in “Methods” section

### XopH degrades phytate

**Fig. 2:**
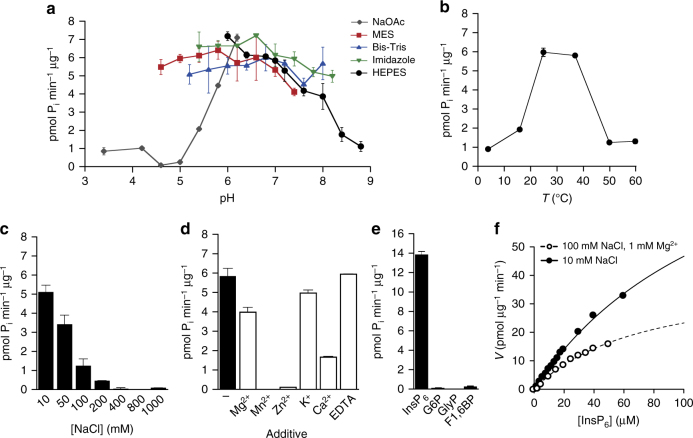
Characterization of the XopH phytase activity. **a** XopH activity in presence of 10 mM NaCl in different buffering systems. **b** XopH activity in presence of 100 mM NaCl and 1 mM MgCl_2_ at different temperatures (16, 25, 37, 50, and 60 °C). **c** XopH activity in buffer with 10–1000 mM NaCl. **d** XopH activity in absence and presence of 1 mM metal ions or 1 mM EDTA. Reaction buffer contained 10 mM NaCl. **e** XopH-dependent dephosphorylation of 0.1 mM InsP_6_, glucose-6-phosphate (G6P), glycerol phosphate (GlyP), and fructose 1,6-bisphosphate (F1,6BP), respectively. **f** Kinetics of InsP_6_ dephosphorylation by XopH. Data were fitted to Michaelis Menten equation using KaleidaGraph4.0 (www.synergy.com). *V*_max_ and *K*_M_ in presence of 100 mM NaCl and 1 mM MgCl_2_ (open circles) are 40.0 ± 5.1 pmol µg^−1^ min^−1^ and 72.9 ± 13.7 µM, and in presence of 10 mM NaCl (closed circles) 110.6 ± 9.5 pmol µg^−1^ min^−1^ and 138.3 ± 15.0 µM. Values are means of two technical replicates. Error bars indicate s.d. The experiments were performed twice with similar results, using two independent protein preparations each

**Fig. 3:**
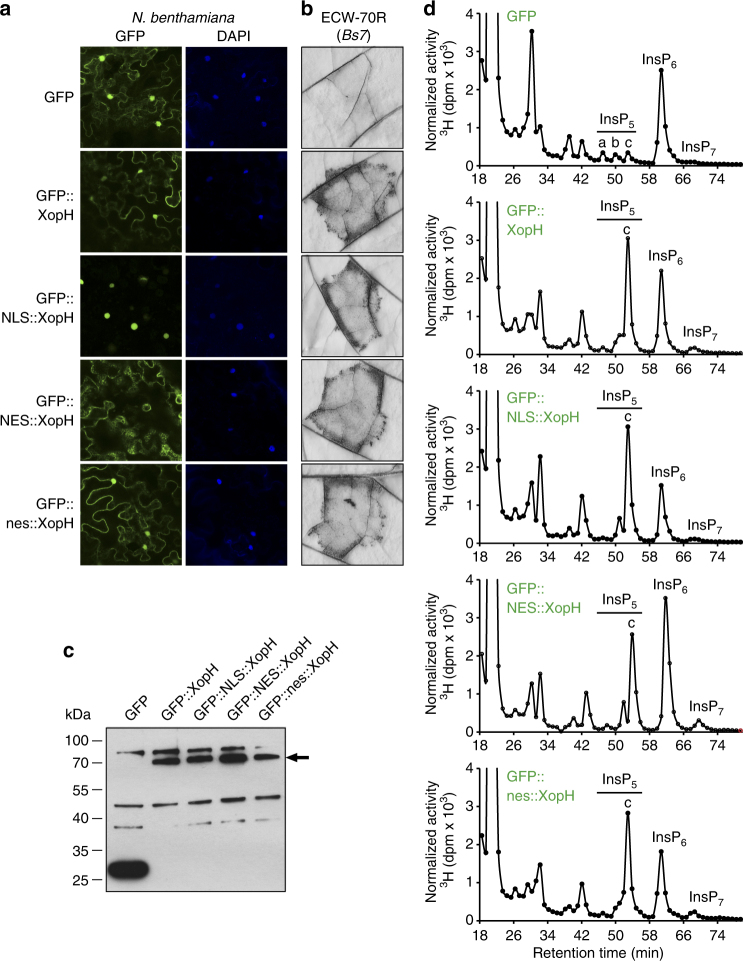
Both cytoplasmic and nuclear-localized XopH variants are biologically active. **a** Confocal laser scanning microscopy of *N. benthamiana* leaves two dpi with *Agrobacterium*-mediating expression of GFP, XopH, or XopH fused to a nuclear localization signal (NLS), a nuclear export signal (NES), and a mutated NES (nes), respectively. DAPI (4′,6′-Diamidino-2-phenylindole) staining indicates nuclei. **b** HR induction by XopH and derivatives. The same *Agrobacterium* strains analyzed in **a** were infiltrated into leaves of resistant pepper ECW-70R plants. Leaves were harvested two dpi and bleached with ethanol. **c** *Agrobacterium*-mediated expression of His_6_-tagged GFP fusion proteins. Samples were harvested two dpi from the same areas as investigated in **a** and analyzed by immunoblot. Signals at expected sizes are marked by an arrow. **d** SAX-HPLC profiles of extracts from [^3^H]-*myo*-inositol-labeled transgenic *N. benthamiana* seedlings that were inoculated with the *Agrobacterium* strains described in **a**. The experiments were repeated twice (**a**–**c**) and once (**d**), respectively, with similar results

XopH structure predictions using Phyre2^14^ revealed a high similarity to the phytase PhyA from the anaerobic bacterium *Selenomonas ruminantium* ^15^ (Fig. 1b). Phytases dephosphorylate *myo*-inositol *hexakis*phosphate (InsP_6_, synonym.: phytate) to lower phosphorylated *myo*-inositol derivatives and *ortho*-phosphate. Phytate is the major phosphate storage compound in plant seeds^16^ and is involved in plant defenses against viral, bacterial, and fungal pathogens^17,18^. We tested InsP_6_ dephosphorylation by XopH in vitro at pH 7.0, which resembles physiological conditions for XopH activity because the protein is localized in both the nucleus and cytoplasm of the plant cell^19^. As shown in Fig. 1c, XopH indeed dephosphorylated phytate. To the best of our knowledge, XopH is the first type III effector with phytate-degrading activity to be reported. The WPD loop mutant H239A retained residual catalytic activity on InsP_6_, whereas the P loop mutant C267A and the double mutant H239A, C267A (CH) displayed a complete loss of function (Fig. 1c). Since mutations in the PRR motifs and deletion of the first 77 amino acids of XopH had only minor effects on its activity (Fig. 1c), the N-terminal region flanking the predicted phytase domain appears to be dispensable for phytate dephosphorylation. This is in agreement with the modular structure of T3Es, which contain a structurally disordered N-terminal region carrying signals for type III-dependent secretion and translocation, and at least one domain for subcellular localization and function in the host cell^1^. Detailed characterization of the XopH phytate-degrading activity revealed a broad pH activity profile with an optimal pH range of 5–7 depending on the buffer used, as well as a rather low temperature optimum of 25 °C compared to other phytases^20^ (Fig. 2a, b). In addition, we tested the effects of ionic strength and different metal ions. XopH activity is highest at low salt condition (Fig. 2c) and inhibited by Zn^2+^, Mn^2+^, and partially by Ca^2+^ and Mg^2+^, but not by the metal ion chelator EDTA (Fig. 2d). There was no detectable XopH-dependent dephosphorylation of the alternative phytase substrates glucose-6-phosphate, glycerol phosphate, and fructose 1,6-bisphosphate^21^ suggesting a high substrate specificity of XopH (Fig. 2e). Using optimized conditions, the specificity constant *k*_cat_/*K*_m_ was found to be 3.28 × 10^5^ M^−1^ **s** ^−1^ (Fig. 2f and Table 1), thus three orders of magnitude higher than the dephosphorylation *k*_cat_/*K*_m_ of the best peptide substrate “pTyr-chip” and comparable to the activity of the *S. ruminantium* phytase^22^. We conclude, therefore, that the primary enzymatic activity of XopH is that of a phytase. Notably, mutant analyses showed that the XopH-induced HR in pepper plants containing the *Bs7* resistance (*R*) gene^9^ depends on the phytase but not protein phosphatase activity (Fig. 1d, e). This suggests that Bs7 recognizes the consequence of XopH activity, e.g., changes in *myo*-inositol polyphosphates, rather than the protein itself. This hypothesis is further corroborated by the fact that changes in the intracellular localization of XopH by fusion to nuclear localization or export signals (NLS, NES) do not significantly affect the HR induction (Fig. 3a–c).

### XopH is a 1-phytase

**Fig. 4:**
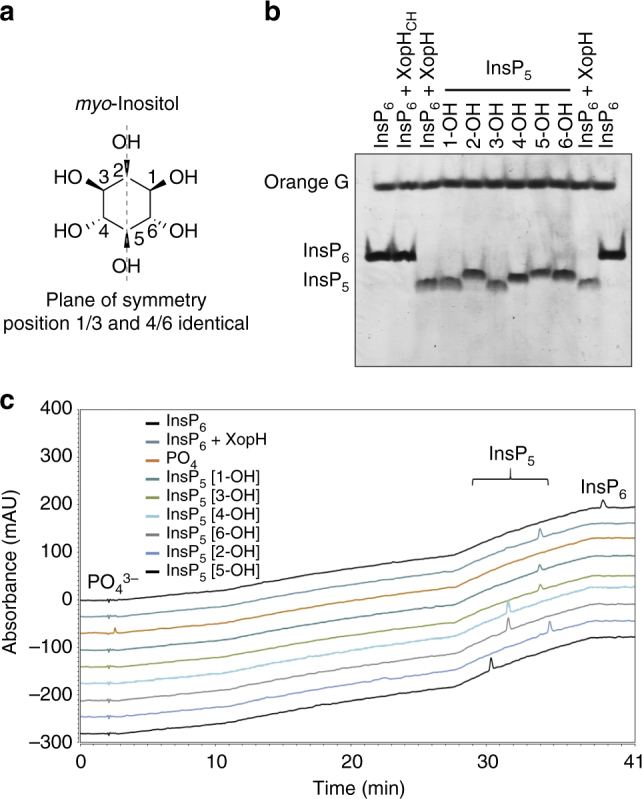
XopH hydrolyzes InsP_6_ to InsP_5_ [1/3-OH]. **a** Structure and symmetry of *myo*-inositol. **b** InsP_6_ (10 nmol) was incubated with XopH or XopH_CH_ (catalytically dead). Reaction products were separated by PAGE and stained. InsP_6_ and InsP_5_ isomers (10 nmol) served as controls. Orange G, loading dye. The experiment was repeated twice with similar results. **c** Stacked ion chromatograms of InsP_6_ (2 nmol), XopH (0.13 µg/µL) + InsP_6_ (2 nmol), a phosphate standard (35 nmol) and all InsP_5_ isomers (2 nmol each) eluted on CarboPac PA100: eluent: 0.5 M HCl and water (18 MΩ * cm); detection: post-column reaction with Fe^3^^+^, UV, 290 nm, injection volume: 10 µL

One possibility to classify phytases is based on the number assigned to the carbon atom of the *myo*-inositol ring at which the first dephosphorylation takes place. *Myo*-inositol itself is a meso-compound with a plane of symmetry dissecting the 2 and 5 positions (Fig. 4a). This plane of symmetry is retained in InsP_6_, however, dephosphorylation at either the 1/3 or 4/6 position will create mirror images (enantiomers) by breaking local symmetry (Supplementary Fig. 2). While plants often express 4-phytases that first target the phosphate at C position 4, activities of all phytase types have been detected in nature, with the exception of 1-phytases^20^. Polyacrylamide gel electrophoresis (PAGE) revealed that the XopH reaction product had the same electrophoretic mobility as InsP_5_ (Fig. 4b); it appears to resist further dephosphorylation by XopH even during extended incubation (up to 24 h, Supplementary Fig. 3a). MALDI-MS and LC-QToF-MS/MS analyses of the XopH reaction product confirmed a main molecular ion peak at 578.9 corresponding to InsP_5_ (Supplementary Fig. 3b-d). PAGE analyses in comparison to the six possible InsP_5_ isomers revealed that the XopH cleavage product displays the same electrophoretic mobility as the enantiomeric 1-OH and 3-OH InsP_5_ isomers (Fig. 4a). InsP_5_ [1/3-OH] isomer identity was corroborated by spiking experiments (Supplementary Fig. 4a) and ion chromatography (Fig. 4c and Supplementary Fig. 3e). To elucidate enantiomer identity of the XopH reaction product, we investigated susceptibility of all InsP_5_ isomers to degradation by XopH. Except for InsP_5_ [1-OH], all isomers (in particular InsP_5_ [3-OH]) were readily degraded, albeit the 2-OH and the 6-OH isomer with lower efficiency (Supplementary Fig. 4b). Importantly, resistance of commercial InsP_5_ [1-OH] to XopH-mediated dephosphorylation was not caused by a contaminating phytase inhibitor since addition of InsP_6_ to the InsP_5_ [1-OH]/XopH reaction mixture resulted in rapid hydrolysis of InsP_6_ to InsP_5_ (Supplementary Fig. 4c). These results suggest that InsP_5_ [1-OH] represents the product of XopH-mediated InsP_6_ hydrolysis.

### A new NMR-based method confirms InsP_5_ [1-OH] as XopH product

**Fig. 5:**
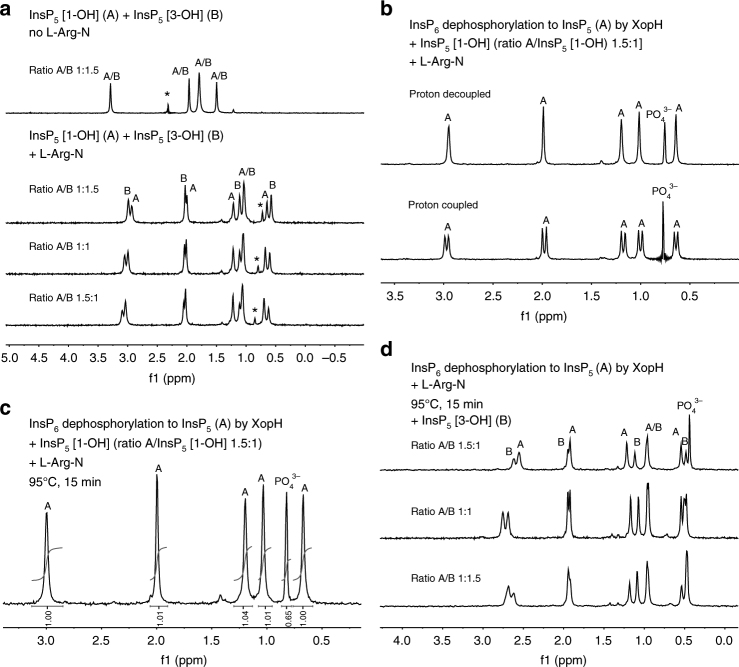
^31^P-NMR in the presence of L-arginine amide identifies InsP_5_ [1-OH] as the XopH reaction product. **a** Upper trace: Mixture of InsP_5_ [1-OH] (A) and InsP_5_ [3-OH] (B) in different ratios in ammonium acetate buffer (pH 7.1) in the absence of L-arginine amide (L-Arg-N). No peak separation was observed and integration gives the expected values 1:1:2:1 (from left to right). Addition of L-Arg-N in excess (ca. 100-fold) leads to separation of the resonances of InsP_5_ [1-OH] (A) or InsP_5_ [3-OH] (B) in all three different ratios studied (1:1.5, 1:1, and 1.5:1). Asterisks mark an impurity. **b** Digest of 600 nmol InsP_6_ by XopH in ammonium acetate buffer (pH 7.1), spiking with 400 nmol InsP_5_ [1-OH] and addition of L-Arg-N in excess (ca. 100-fold). No additional peaks can be seen in a proton-decoupled spectrum (upper trace). A proton-coupled spectrum identifies all resonances belonging to inositol-bound phosphates (A). **c** Digest of 600 nmol InsP_6_ by XopH, spiking with InsP_5_ [1-OH] and addition of L-Arg-N in excess (ca. 100-fold). The mixture was heated to 95 °C for 15 min to denature residual XopH. Integration identifies the phosphate resonance (expected ratio is 1.0:0.6) and shows that no decomposition after boiling takes place. **d** XopH digest of 600 nmol and excess L-Arg-N (ca. 100-fold) boiled at 95 °C for 15 min. Subsequent addition of InsP_5_ [3-OH] (B) in different ratios leads to appearance of new resonances

To unambiguously reveal isomer identity of the XopH cleavage product, we developed a novel method based on nuclear magnetic resonance (NMR) spectroscopy, which can be applied to samples in the presence of buffers and salts that do not interfere with ^31^P-NMR. As briefly discussed above, *myo*-inositol and phytate are meso-compounds displaying an internal plane of symmetry (Fig. 4a and Supplementary Fig. 2). Thus, for phytate a ^31^P-NMR shows two distinct resonances for the phosphates in the 2 and 5 positions, one for the two phosphates in the 1 and 3 positions and another one for the 4 and 6 positions due to internal symmetry. The integrals of the resonances, therefore, display a ratio of 1 (position 2):2 (positions 1 and 3):2 (positions 4 and 6):1 (position 5) (Supplementary Fig. 5a, b). Breaking the symmetry by dephosphorylation in either the 1/3 or 4/6 position will result in distinct signals for every single phosphate, even though they may accidentally have the same chemical shift. Using NMR, one cannot discriminate enantiomers, as they will generate identical resonances in an environment that is achiral (Fig. 5a, top trace). Our method is based on the addition of an enantiopure counter ion that forms one or multiple ion pairs with the inositol phosphate giving rise to diastereomeric salts. This will generate different local environments for the atoms and, therefore, different chemical shifts of the resonances. Such strategies have been developed for other acidic compounds mainly based on ^1^H-NMR^23,24^. Since phosphates associate tightly with guanidinium groups, we screened several candidate compounds and identified L-arginine amide hydrochloride salt (L-Arg-N) as a suitable counter ion to generate diastereomeric ion pairs with InsP_5_ even in aqueous buffer. Separate ^31^P-NMR measurements of both InsP_5_ [1-OH] and InsP_5_ [3-OH] in the presence of excess L-Arg-N resulted in slightly different shifts for all five peaks (Supplementary Fig. 6). Since ^31^P-NMR chemical shifts are very sensitive to the matrix and especially small changes in pH^25^, we performed spiking experiments mixing InsP_5_ [1-OH] and InsP_5_ [3-OH] in different ratios (1:1.5, 1:1, and 1.5:1). While the chiral non-racemic mixture showed four peaks in a ratio of 1:1:2:1, addition of an excess of L-Arg-N caused a large shift in all resonances and led to resolvable differences for four of the five phosphate signals of both enantiomers (Fig. 5a). This effect was observed in all three different ratios measured. Thus, ^31^P-NMR can be used to identify enantiomeric inositol phosphates in the presence of excess L-Arg-N as chiral solvating agent.

We used our method to analyze the XopH hydrolysis product without purification in phosphate-free buffer. Furthermore, commercial InsP_5_ [1-OH] was added to the reaction mixture. The spectrum showed the expected five resonances for InsP_5_ and an additional resonance for phosphate that was easily identified by (i) recording of a proton-coupled spectrum (Fig. 5b, lower trace) and (ii) integration of the peaks (Fig. 5c). The proton-coupled spectrum showed peak splitting (doublets) only for the phosphates bound to the inositol scaffold, whereas in PO_4_^3−^ no bound protons were available for coupling. The spiked spectrum also showed that the integral of PO_4_^3−^ was smaller than those of the other resonances, as only InsP_5_ [1-OH] was added. Heating of the sample to destroy residual XopH activity did not cause decomposition of InsP_5_ (Fig. 5c). Next, a XopH digest obtained as described above was inactivated at 95 °C and then spiked with InsP_5_ [3-OH] in different ratios in presence of an excess of L-Arg-N. As with the InsP_5_ [1-OH/3-OH] mixture, a clear separation of four of the five phosphate signals was achieved and easily assigned by peak intensity (Fig. 5d). Notably, addition of InsP_5_ [1-OH] under the same conditions did not lead to the separation of any of the resonances (Fig. 5c). In combination with the PAGE and IC analyses, we conclude that the XopH reaction product corresponds to InsP_5_ [1-OH]. Hence, XopH is the first naturally occurring 1-phytase.

### XopH leads to InsP_5_ accumulation in vivo

**Fig. 6:**
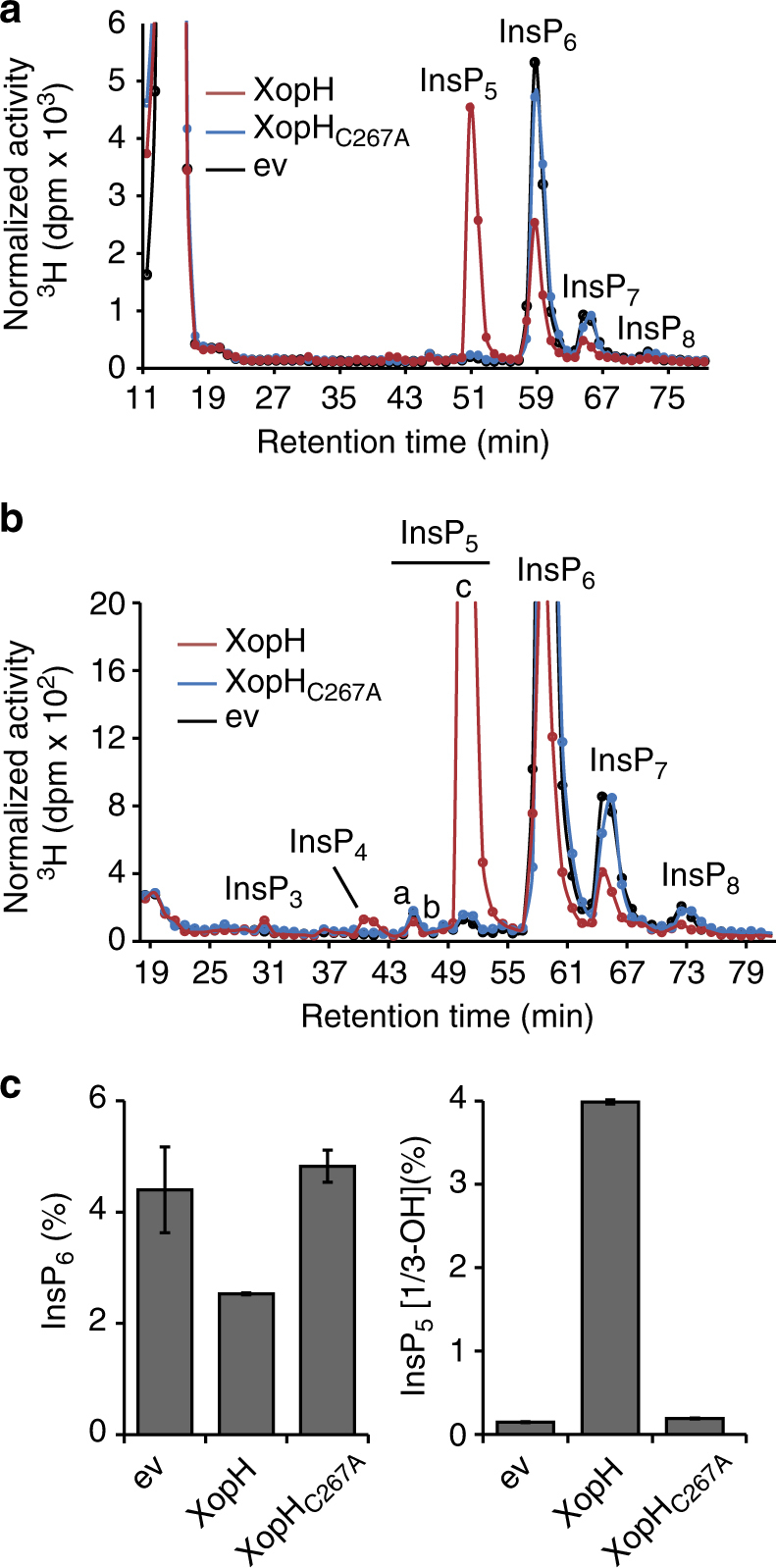
XopH changes InsP_x_ contents in yeast. **a** HPLC profile of extracts from yeast transformants labeled with [^3^H]-*myo*-inositol. ev empty vector. Based on published chromatographic mobilities^69^, InsP_5a_ represents InsP_5_ [2-OH], InsP_5b_ represents InsP_5_ [4/6-OH], and InsP_5c_ represents InsP_5_ [1/3-OH]. The isomeric natures of InsP_3_, InsP_4_, InsP_7_, and InsP_8_ are unknown. **b** Zoom-in on the HPLC profile. **c** Relative amounts of InsP_6_ and InsP_5c_ in the yeast transformants. Error bars indicate s.e.m. The experiment was repeated independently with similar results

To analyze the consequences of XopH phytate-degrading activity in vivo, we compared the composition of *myo*-inositol polyphosphates in *Saccharomyces cerevisiae* ectopically expressing XopH or the phytase-inactive XopH_C267A_ mutant, by strong anion exchange (SAX) HPLC, a method that by itself does not allow to discriminate between enantiomers in the absence of chiral selectors. Ectopic expression of XopH in yeast caused a strong reduction of InsP_6_ and a robust accumulation of InsP_5_ with the chromatographic mobility of InsP_5_ [1/3-OH] (Fig. 6). Furthermore, an increase in one InsP_4_ and one InsP_3_ species, both of unknown stereochemistry, was detected. By contrast, expression of the catalytically dead mutant XopH_CH_ did not cause significant changes in the HPLC profile as compared to the control (Fig. 6).

**Fig. 7:**
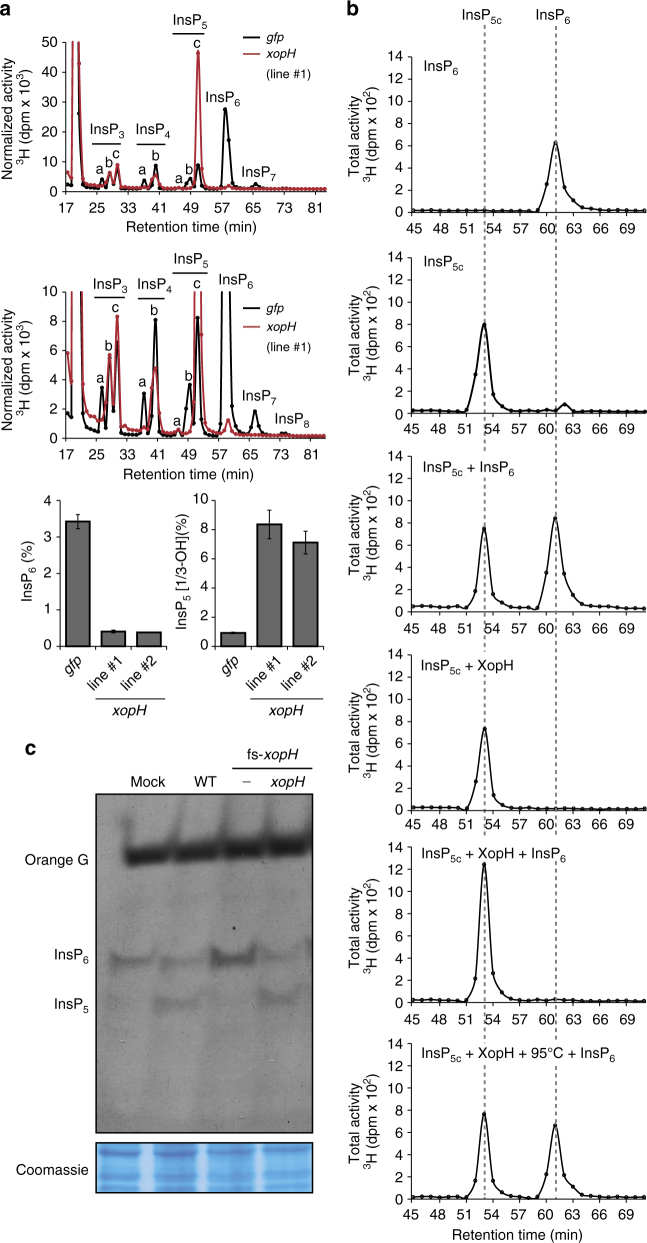
XopH causes InsP_5_ accumulation in planta. **a** Upper panel: HPLC profiles of extracts from transgenic *N. benthamiana* leaves, labeled with [^3^H]-*myo*-inositol. Based on published chromatographic mobilities^69^, InsP_5a_ represents InsP_5_ [2-OH], InsP_5b_ represents InsP_5_ [4/6-OH], and InsP_5c_ represents InsP_5_ [1/3-OH]. The isomeric natures of InsP_3a-c_, InsP_4a-b_, InsP_7_, and InsP_8_ are unknown. Middle panel: Zoom-in on the HPLC profiles. Lower panels: Relative amounts of InsP_6_ and InsP_5c_ in *N. benthamiana* expressing *gfp* and two independent lines expressing *xopH*. Error bars indicate s.e.m. **b** Digestion and HPLC analyses of plant-purified InsP_x_ species. InsP_5c_ and InsP_6_ were purified from [^3^H]-*myo*-inositol-labeled *xopH-* and *gfp-*expressing *N. benthamiana* seedlings, respectively (see “Methods” section). XopH-treated or non-treated InsP_x_ species (as designated) were then separated by SAX-HPLC. XopH was inactivated by incubating the reaction mixture at 95 °C for 15 min. **c** XopH hydrolyzes InsP_6_ to InsP_5_ during *Xcv* infection. TiO_2_ bead enrichment of inositol polyphosphates from *N. benthamiana* leaf extracts infected with *Xcv* 85-10Δ*xopQ* (WT), 85-10Δ*xopQ*_fs-*xopH* (fs-*xopH*), and the complemented mutant, where *xopH* was re-integrated into the genome (see “Methods” section). Inositol polyphosphates were eluted from TiO_2_ beads, resolved by PAGE and visualized by toluidine blue staining. Protein extracts were visualized by Coomassie blue as a loading control. The experiments were repeated twice (**a**, **c**) or once (**b**) with similar results

In agreement with a role of XopH in InsP_6_ dephosphorylation, transgenic *Nicotiana benthamiana* plants constitutively expressing *xopH* showed a robust accumulation of InsP_5_ [1/3-OH] at the cost of InsP_6_ (Fig. 7a and Supplementary Fig. 7). Altered localization of XopH to only nucleus or cytoplasm had no major effect on its ability to hydrolyze InsP_6_ (Fig. 3d), a small molecule likely to diffuse freely between nucleo- and cytoplasm. Given the biochemical data described above, the observed InsP_5c_ isomer likely represents InsP_5_ [1-OH]. To test this idea, InsP_5c_ and InsP_6_ were purified from [^3^H]-*myo*-inositol-labeled *xopH*- and *GFP*-transgenic *N. benthamiana* seedlings, subjected to digest by XopH and separated by SAX-HPLC. Under these conditions, XopH did not further dephosphorylate InsP_5c_, whereas purified InsP_6_ was readily degraded, thus increasing the InsP_5c_ peak (Fig. 7b and Supplementary Fig. 8). This is consistent with the XopH-dependent InsP_5c_ corresponding to InsP_5_ [1-OH] which, in contrast to all other InsP_5_ isomers, is resistant to XopH-mediated dephosphorylation (see above). The observation that InsP_5_, presumably the InsP_5_ [1-OH] enantiomer, strongly accumulates and is not degraded further by plant enzymes implies that suitable endogenous phosphatases might be absent in *N. benthamiana*.

To investigate whether InsP_5_ [1-OH] accumulates in the plant during natural infection, i.e., after XopH translocation via the *Xcv* T3S system, we inoculated the *Xcv* WT strain, a *xopH* frameshift mutant and the complemented mutant into *N. benthamiana*. InsP_6_ and InsP_5_ contents were determined by PAGE after titanium oxide (TiO_2_) bead enrichment. Although *N. benthamiana* is not a natural host of *Xcv*, we recently showed that this is solely due to the recognition of a single T3E, XopQ, which triggers a defense reaction and prevents bacterial growth^26^. Deletion of *xopQ* turns *Xcv* strain 85-10 into a *N. benthamiana* pathogen that grows in the tissue and elicits typical disease symptoms, i.e., water-soaked lesions^26^. As shown in Fig. 7c, inoculation of 85-10Δ*xopQ* (“WT”) induced a slight decrease in the plant's InsP_6_ content and an additional InsP_5_ signal. By contrast, leaves inoculated with the respective *xopH* frameshift mutant (85-10Δ*xopQ*_fs-*xopH*) contained even more InsP_6_ than mock-infiltrated plant tissue and no InsP_5_ in detectable amounts. 85-10Δ*xopQ*_fs-*xopH* was complemented by re-integration of *xopH* into the genome (Fig. 7c).

In addition, we analyzed XopH-dependent changes in InsP_6_ and InsP_5_ contents in the *Xcv*-pepper system. Although InsP_5_ was barely detectable in pepper, there was a clear InsP_6_ decrease after inoculation of strains expressing XopH, but not the inactive XopH_CH_ mutant version (Supplementary Fig. 9). Notably, the *xopH* frameshift mutant was complemented by expression of XopH_Del77_ confirming that the phytase, and not the protein phosphatase, activity is responsible for the observed InsP_6_ decrease. Leaf tissue inoculated with a T3S-deficient strain (Δ*hrcN*) showed a similar InsP_6_ content as that infected with the *xopH* mutant. This suggests that XopH is the only T3E in *Xcv* strain 85-10, which significantly affects phytate levels.

### XopH affects plant hormone pathways

**Fig. 8:**
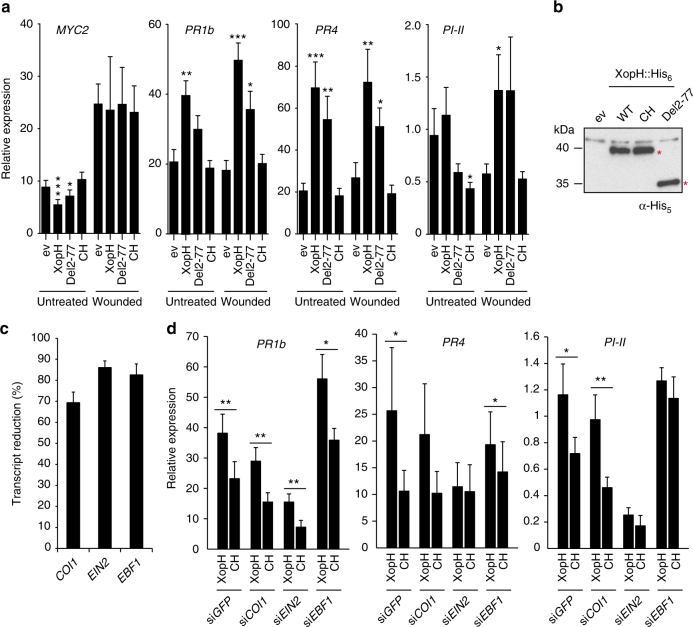
XopH phytase activity affects plant hormone pathways. **a** qRT-PCR analysis of *N. benthamiana* leaves transiently expressing XopH and mutant derivatives, untreated or 20 min post wounding. ev empty T-DNA. Asterisks indicate statistically significant differences to corresponding ev samples (Mann–Whitney test; *p < 0,05; **p < 0,01; ***p < 0,001). **b** Protein expression two dpi in the same plants analyzed in **a**. Immunoblot signals at expected sizes are marked by asterisks. **c** qRT-PCR analysis of *COI1*-, *EIN2*-, and *EBF1*-silenced *N. benthamiana* plants (si*COI1*, si*EIN2*, and si*EBF1*) transiently expressing XopH and its catalytically inactive variant (CH), respectively. *GFP*-silenced plants (si*GFP*) served as control. Asterisks indicate statistically significant differences (Student’s *t* test; *p < 0,05; **p < 0,01). **d** Silencing efficiencies determined by qRT-PCR. Values indicate percent reduction of *COI1*, *EIN2*, and *EBF1* transcript levels in the respective silenced plants, relative to *GFP*-silenced plants, both expressing inactive XopH_CH_. Combined data from three independent experiments (with three biological replicates per experiment) are shown (**a**, **c**, **d**). Error bars indicate s.e.m.

Plant hormone signaling pathways can involve InsP_x_ co-factors, as was suggested for auxin and jasmonate (JA) signal transduction^27–29^, and might, therefore, be affected by changes in the InsP_x_ homeostasis. XopH expression in *N. benthamiana* leaves caused a strong concomitant reduction of inositol pyrophosphates InsP_7_ and InsP_8_ (Fig. 7a and Supplementary Fig. 7). The latter is required for jasmonate perception, most likely by inducing an allosteric switch of the jasmonate receptor complex^18,29^. We analyzed the influence of XopH expression on transcript abundance of JA-responsive genes in *N. benthamiana* leaves without or 20 min after wounding. qRT-PCR analysis revealed that XopH led to a slightly reduced *MYC2* expression, but induced *PR1b*, *PR4*, and *PI-II* (Fig. 8a, b), which were used as general JA marker genes in previous studies in solanaceous plants^30,31^. Weaker induction was observed with XopH_Del77_, which is consistent with the reduced activity of this XopH variant (Fig. 1c, d) and demonstrates that the phytase, and not the protein phosphatase, activity is required for gene induction (Fig. 8a, b).

Notably, *PR1b*, *PR4*, and *PI-II* genes are not only JA responsive, but also ethylene (ET) responsive^32,33^, suggesting that XopH might affect the ET pathway. Consistent with this idea, transgenic *N. benthamiana* plants constitutively expressing *xopH* were significantly smaller than transgenic *GFP* control plants of the same age and showed signs of early senescence (Supplementary Fig. 10). We performed virus-induced gene silencing of *EIN2* and *EBF1* encoding a positive and negative regulator of the ET pathway, respectively^34^, in *N. benthamiana* and analyzed the effect on XopH-mediated induction of *PR1b*, *PR4*, and *PI-II*. In addition, we silenced *COI1*, the positive master regulator of the JA pathway^35^. A *GFP* silencing construct served as control. While *COI1* silencing revealed no obvious involvement of the JA pathway in XopH-dependent gene induction, silencing of ET pathway components (especially *EIN2*) suppressed the upregulation of *PR4* and *PI-II*, but not *PR1b* (Fig. 8c, d).

## Discussion

XopH is a T3E with a novel phytate-degrading activity, which we demonstrated in vitro and in planta. Using a combination of different digestion experiments of chemically pure inositol polyphosphates and an NMR-based method to discriminate InsP_5_ isomers, we identified XopH as the prototype of a new class of phytases initiating InsP_6_ dephosphorylation at C position 1. Thus, XopH belongs to the relatively small number of *Xanthomonas* T3Es with identified biochemical activities: the large group of TAL effectors and T3Es with uridylyltransferase (AvrAC), acetyltransferase/cysteine protease (YopJ/AvrRxv family), SUMO protease (XopD), and ubiquitin ligase activity (XopL)^1^.

Our mutant analyses indicate that the phytase and not the protein phosphatase activity is relevant for XopH's biological activities, i.e., HR elicitation in the host plant pepper (Fig. 1d) and gene induction in *N. benthamiana* (Fig. 8a). In this respect, XopH has a different physiological function than its homolog HopAO1 (formerly termed HopPtoD2; 25% identical to XopH) from *Pseudomonas syringae* pv. *tomato*. HopAO1 was reported to reduce tyrosine phosphorylation of the PAMP receptor EFR, after recognition of the elf18 peptide from bacterial elongation factor Tu, thus preventing immune responses^36^. By contrast, XopH does not inhibit elf18-inducible gene expression^19^. Furthermore, membrane proteins, such as EFR (or other proteins that do not enter the nucleus), are unlikely targets of XopH, because nuclear or cytoplasmic XopH variants exhibit similar HR-inducing activities in resistant pepper plants (Fig. 3), and both virulence and HR-inducing activities of T3Es are often connected (see below). Notably, we found that HopAO1 degraded phytate in vitro but, in contrast to XopH, to lower phosphorylated *myo*-inositol derivatives (Supplementary Fig. 11). In conclusion, this suggests different activities for XopH and HopAO1.

The fact that only the phytase domain of XopH is essential for HR induction suggests that Bs7 recognizes the product of the enzyme reaction, i.e., InsP_5_ [1-OH], or changes caused by altered inositol polyphosphate homeostasis rather than the XopH protein itself. This hypothesis is supported by the fact that XopH variants solely localizing to the nucleus or the cytoplasm of the plant cell have the same HR-inducing activity as the WT protein, which correlates with similar phytase activities (Fig. 3). Indeed, most R proteins recognize their cognate T3Es indirectly by detecting effector-triggered changes in plant targets (guard model) or non-functional target mimics (decoy model)^1^. Notably, the molecules targeted by T3Es are usually proteins or, as in case of TAL effectors, DNA sequences and not low-molecular substances like inositol polyphosphates.

What are the consequences of phytate dephosphorylation and InsP_5_ [1-OH] accumulation for susceptible plant cells and the bacterial pathogen, which lives in the intercellular spaces? One possibility is that XopH liberates phosphate from the plant tissue to improve the nutritional status of the pathogen. A role in phosphate acquisition from the extracellular milieu has been reported for the putative phytase PhyA secreted by the rice pathogen *X. oryzae* pv. *oryzae*. PhyA_*Xoo*_ enhances bacterial growth in medium with phytate as sole phosphate source and is required for virulence^37^. Another scenario supported by our qRT-PCR data is that XopH interferes with plant hormone pathways, probably by affecting the abundance of potential co-factors. According to this hypothesis, our data give a first hint that InsP_x_ co-factors are also involved in the ET pathway. Since ET acts synergistically with JA to regulate resistance against necrotrophic pathogens^34^, stimulation of the ET pathway might be beneficial to hemibiotrophic bacteria like *Xcv*. Furthermore, bacterial dispersal may benefit from ET functions in disease development at later infection stages^38^. Notably, XopH has been recently reported to promote disease symptom formation on tomato^19^. A third model is that XopH compromises plant defense mechanisms by degradation of InsP_6_, which has been suggested to play a role in resistance against a wide variety of pathogens^17,18^. XopH might sequester InsP_6_ by degrading it to an InsP_5_ isomer, which is not easily metabolized by the plant (as shown above) and accumulates. Indeed, XopH has been recently shown to inhibit defense reactions, e.g., callose deposition, in Arabidopsis^19^. A similar strategy is imaginable for the phytase LppA from the human pathogen *Legionella pneumophila*: while the product of LppA activity is unknown, LppA has been shown to be translocated via the type IV secretion system and to counteract intracellular bacterial growth restriction by phytate^39^. By taking advantage of an enantiomer-specific *Dictyostelium* InsP_5_ [3-OH] kinase activity, the InsP_5c_ species in mung bean seedlings was proposed to largely consist of InsP_5_ [1-OH]^40^. Future work will have to clarify isomer identity and physiological role of the endogenous (XopH-independent) InsP_5c_ species in *N. benthamiana* and pepper. Because InsP_6_ is ubiquitous in eukaryotes and is known to have critical roles in these systems^16^, we believe that the mechanism employed by *Xanthomonas* could be a common strategy of host immune system avoidance for bacteria and other pathogens. Therefore, our discovery might provide the basis for the development of new strategies to overcome or attenuate crop disease caused by bacterial pathogens.

In addition, the novel activity of XopH reported here adds to the molecular toolbox for future biotechnological manipulation of phytate levels. Because of its high affinity for minerals including iron and zinc ions, phytate is considered an antinutrient for humans and monogastric animals (swine, poultry, and fish), all of which lack the ability to significantly degrade phytate^20^. Hence, phytases are economically important enzymes that are widely used to improve phosphate and mineral availability in animal feed and to reduce phosphate excretion by animals^20^. Furthermore, the novel 1-phytase specificity of XopH gives access to a new set of inositol phosphate isomers. InsP_5_ [1-OH] and its derivatives generated by other phosphatases will, for instance, prove useful for the future characterization of new phytases and phosphatases.

## Methods

### Plant material and inoculations

*Nicotiana benthamiana* and pepper (*Capsicum annuum*) cultivar ECW-70R (*Bs7*)^9^ plants were grown in the greenhouse under standard conditions (day and night temperatures of 23 °C and 19 °C, respectively, for *N. benthamiana*, and 25 °C and 19 °C for pepper, with 16 h light and 40–60% humidity). For in planta protein expression, mature leaves of 5- to 7-week-old plants were inoculated with *Agrobacterium tumefaciens* adjusted to OD_600_ = 0.8 in infiltration medium (10 mM MES pH 5.5, 10 mM MgCl_2_, 150 µM acetosyringone) using a needleless syringe. *Xcv* was inoculated (in 10 mM MgCl_2_; OD_600_ = 0.1 or 0.02) into leaves of 6-week-old pepper or *N. benthamiana* plants using a needleless syringe. Inoculated pepper plants were transferred to a Percival growth chamber (Percival Scientific, Perry, USA).

### Bacterial strains and growth conditions

*Escherichia coli* BL21(DE3) (Agilent Technologies Inc., Santa Clara, USA) and TOP10 (Life Technologies GmbH, Darmstadt, Germany) were cultivated at 37 °C in LB (lysogeny broth) medium^41^, *A. tumefaciens* GV3101^42^ and derivatives at 30 °C in YEB (yeast extract broth) and *Xcv* strain 85-10^3^ and derivatives on NYG (nutrient yeast glycerol) agar plates^43^ supplemented with appropriate antibiotics. Plasmids were introduced into *E. coli* and *A. tumefaciens* by electroporation, and into *Xcv* by conjugation using pRK2013 as helper plasmid in triparental matings^44^.

### Construction of *xopH* mutants in *Xcv*

The *xopH* frameshift mutant (fs-*xopH*) carries a 4-bp insertion after nucleotide (nt) position 53 of the *xopH* ORF generating a *Pfo*I restriction site and resulting in an early stop codon at nt position 76. For this, plasmid pOK-early-stop-xopH was used containing two ~670-bp PCR fragments from chromosomal DNA of *Xcv* strain 85-10, which were combined in pUC57 prior to PCR-based transfer into suicide vector pOK1^45^. Positive *Xcv* 85-10_fs-*xopH* colonies were identified by *Pfo*I digestion and sequencing of PCR amplicons. To generate a Δ*xopQ*_fs-*xopH* double mutant, suicide plasmid pOGG2:xopQ was used^26^.

### XopH derivatives and controls

Generally, DNA fragments were amplified using Hybrid DNA Polymerase (Roboklon, Berlin, Germany) unless stated otherwise. All cloned fragments were sequenced. Oligonucleotides are listed in Supplementary Table 1. Constructs were generated by GATEWAY^46^ and In-Fusion HD (Takara Bio USA, Inc., Mountain View, USA) cloning technology according to the manufacturer's protocols.

To generate expression constructs for XopH-His_6_ and XopH-Del2-77-His_6_, PCR-amplified *xopH* fragments were cloned into pENTR/D-TOPO (Thermo Fisher Scientific, Darmstadt, Germany). To generate pENTR/D:xopH-C267A-his_6_, the Phusion Site-Directed Mutagenesis Kit (Thermo Fisher Scientific) was used according to the manufacturer's protocol. pENTR/D:xopH-H239A-his_6_, pENTR/D:xopH-mutP48-52-53-his_6_ (PRR1 mutant), pENTR/D:xopH-mutP69-71-his_6_ (PRR2-1 mutant), and pENTR/D:xopH-mutP73-74-75-76-his_6_ (PRR2-2 mutant) were derived from pENTR/D:xopH-his_6_ using the QuikChange Lightning Site-Directed Mutagenesis Kit (Agilent Technologies). To generate pENTR/D:xopH-C267A-H239A-his_6_ (CH), pENTR/D:xopH-C267A-his_6_ served as template for site-directed mutagenesis. Entry clones were transferred into the GATEWAY-compatible expression vectors pAG416-GAL-ccdB-HA for yeast (gift from Susan Lindquist; Addgene plasmid #14243) and pGWB2 for plants^47^.

For localization studies, constructs for expression of GFP-XopH-His_6_ fusions with nuclear import (NLS), export (NES), or mutated export (nes) signal were generated by SOE (splicing by overlap extension) PCR using Q5 High Fidelity Polymerase (New England BioLabs, Frankfurt/Main, Germany), cloned into pENTR/D-TOPO and transferred into pGWB2. As control, PCR-amplified *gfp* was cloned into pENTR/D-TOPO and transferred into pGWB408^48^.

For expression in *E. coli*, PCR-amplified *xopH-his*_*6*_ and *xopH-del2-77-his*_*6*_ fragments were cloned into *Nde*I/*Xho*I-digested pET22b(+) vector (Merck Millipore, Darmstadt, Germany) using In-Fusion HD cloning.

For complementation, *xopH*,* xopH*_*CH*_, and *xopH*_*Del2-77*_, respectively, under control of the native *xopH* promoter (344 bp) and followed by the *avrBs1* terminator region (208 bp) was inserted into a defined site into the genome of fs-*xopH* mutants using derivatives of the suicide vector pLAND-P^49^. To allow translocation of XopH_Del2-77_ by the *Xcv* T3S system, a 231-bp fragment encoding the sequence-unrelated T3S signal of AvrBs3 (aa 2–78) was cloned in front of *xopH*_*Del2-77*_. Cloning details are available upon request.

### Generation of transgenic *xopH* plants

*N. benthamiana* was transformed using *Agrobacterium* GV3101 containing pGWB2:xopH-his_6_ and pGWB2:xopH-H239A-C267A-his_6_, respectively. Transgenic plants were confirmed by PCR and immunoblot. Here, T2 plants were used.

### Virus-induced gene silencing

For silencing of *COI1*, *EIN2*, and *EBF1*, 190-, 434-, and 251-bp fragments, respectively, were PCR-amplified from *N. benthamiana* cDNA and cloned into pENTR/D-TOPO (ThermoFisher Scientific). Oligonucleotides are listed in Supplementary Table 1. The fragments were recombined into the GATEWAY-compatible tobacco rattle virus (TRV) RNA2 vector pYL279A^50^. VIGS was initiated by *Agrobacterium*-mediated co-delivery of pYL279A derivatives and pTRV-RNA1^51^.

### Immunoblot analysis

Plant samples were prepared and analyzed as described^52^ using an α-His_5_ antibody (Qiagen, Hilden, Germany; Cat No./ID: 34660; diluted 1:2000). Unprocessed blot images are shown in Supplementary Fig. 12.

### qRT-PCR analysis

Five *N. benthamiana* plants were inoculated with *A. tumefaciens* GV3101 containing pGWB2:xopH-his_6_, pGWB2:xopH-del2-77-his_6_, pGWB2:xopH-H239A-C267A-his_6_, or empty pGWB2. For better protein expression, the strains were mixed 1:1 with GV3101 mediating expression of silencing inhibitor p19^53^. Three dpi, two leaf discs per plant (Ø nine mm), i.e., five biological replicates, were harvested before and 20 min after wounding of the leaf with forceps.

Silencing plants were analyzed 14–15 days after VIGS initiation. Nine plants per silencing construct were inoculated with GV3101 containing pGWB2:xopH-his_6_ and pGWB2:xopH-H239A-C267A-his_6_, respectively, and two leaf discs per plant were harvested. Three plants were pooled per biological replicate.

Total RNA was isolated from leaf samples using standard Trizol reagent^54^. An aliquot of 5 µg of total RNA was reverse-transcribed using RevertAid Reverse Transcriptase (Thermo Fisher Scientific). qPCR analyses were performed on an Mx3005P qPCR System (Agilent Technologies) using the Maxima SYBR Green/ROX qPCR Master Mix (Thermo Fisher Scientific) and oligonucleotides listed in Supplementary Table 1. Data are shown relative to the house-keeping genes *F-Box* and *LR23*.

To ensure adequate statistical power, qPCR data of three independent experiments were combined and statistically analyzed (two-group comparisons; *n* ≥ 9). For XopH overexpression experiments (Fig. 8a), the two-tailed Mann–Whitney test (non-parametric) was used. For VIGS experiments (Fig. 8d), one-tailed paired *t* tests were performed (XopH_CH_ served as reference, assuming induction of transcript accumulation by WT XopH).

### Protein expression in *E. coli* and purification

Freshly transformed *E. coli* BL21(DE3) cells carrying recombinant pET22b(+) and pSKE6, respectively, were cultivated overnight in LB broth containing 50 µg mL^−1^ ampicillin or kanamycin and 0.5% glucose (37 °C, 160 rpm). For expression, LB broth containing 100 µg mL^−1^ ampicillin or 50 µg mL^−1^ kanamycin was inoculated at OD_600_ = 0.05 with overnight culture and incubated (37 °C, 160 rpm). At OD_600_ = 0.8, protein expression was induced by 1 mM IPTG and 3% ethanol (final concentration). After 5 h, cells were harvested by centrifugation at 5000 rpm (Beckman JA-10 fixed-angle rotor) for 10 min at 4 °C. Cell pellets were stored at −20 °C until protein preparation.

For XopH protein preparation, cell pellets were resuspended in 100 mM HEPES pH 7.5, 300 mM NaCl, 0.5% glycerol, 10 mM imidazole containing 1 mg mL^−1^ lysozyme, and EDTA-free protease inhibitor cocktail (Roche, Sigma-Aldrich, Munich, Germany). After 30 min on ice, cells were disrupted by three freeze-thaw cycles (30 °C water bath, liquid nitrogen). After 30 min incubation on ice with 50 µg mL^−1^ DNase 1/RNase A, cell lysate was collected by centrifugation (20,000×*g*, 30 min, 4 °C). Proteins were purified using HisLink™ Protein Purification Resin (Promega, Mannheim, Germany) according to the manufacturer's protocol. Wash buffer: 100 mM HEPES pH 7.5, 300 mM NaCl, 0.5% glycerol, 10 mM imidazole; elution buffer: 100 mM HEPES pH 7.5, 300 mM NaCl, 0.5% glycerol, and 500 mM imidazole. Protein concentration was determined using the molar extinction coefficient^55^. Protein purity (>90%) was checked by SDS-PAGE and Coomassie staining.

The construct pMCSG57-hopAO1 for the synthesis of full-length HopAO1, N-terminally fused to 6xHis, was obtained from the DNASU plasmid repository (https://dnasu.org/DNASU/Home.do). His_6_::HopAO1 was expressed in *E. coli* BL21-CodonPlus (DE3)-RIL cells (Stratagene, San Diego, USA) and purified similar to the yeast Sfh1 protein^56^. Prior to activity assays, the purified protein was dialyzed in buffer containing 300 mM NaCl, 25 mM Na_2_HPO_4_ (pH 7.5), and 5 mM β-mercaptoethanol.

### General phosphatase activity

Phosphatase activity using 0.25 µg µL^−1^ protein and 20 mM pNPP as a substrate was analyzed at 37 °C with the JBS Phosphatase Assay Kit (Jena Bioscience, Jena, Germany) according to the manufacturer's protocol. Reaction buffer contained 100 mM NaCl, 1 mM MgCl_2_, 5% glycerol, 0.1% ß-mercaptoethanol in 50 mM MES, HEPES, or sodium acetate, and different pH. Extinction at 405 nm was recorded using a Spectrostar Nano microplate reader (BMG Labtech, Ortenberg, Germany). The experiment was done twice with similar results, using two independent protein preparations each.

### Phosphopeptide microarray

The 13-mer peptide microarray used for profiling the XopH-phosphatase activity consists of 6207 peptides, each containing one phospho-tyrosine residue in the central position^57–59^. Each phosphopeptide is displayed in triplicates on the glass surface enabling quality control and intra-chip reproducibility. Peptides were synthesized by SPOT synthesis technology on cellulose membranes according to R. Frank^60^. After deprotection of side chains by 95% trifluoroacetic acid, spots were punched out into wells of 96-microtiter plates. Phosphopeptides were released from the cellulose membrane by treatment with 5% aqueous triethylamine. After separation of the peptide solution from the cellulose disks and evaporation of the cleavage solution peptide derivatives were redissolved in printing buffer and transferred into 384-well plates. Peptides were printed onto epoxy-functionalized glass slides using an OmniGrid300 contact printer. Finally, microarray surfaces were passivated at 40 °C using bovine serum albumin in citrate buffer.

Peptide microarrays reacted with XopH or the C267A variant yielded 82 and 238 substrate peptides, respectively, showing more than 70% signal decrease as compared to the control experiment without enzyme. Two types of control experiments were performed to distinguish between signal decrease caused by enzymatic action and signal decrease because of enzyme bound to the immobilized phosphopeptides and masking the epitope for the phospho-specific antibody (P100; Cell Signaling Technologies, Leiden, the Netherlands). First, peptide microarrays were treated with α-XopH-antibody (BioGenes, Berlin, Germany; polyclonal α-peptide antibody against aa 43–56: ELADLPSRQPPRSK) to check if mutant binds to phosphopeptides (trapping mutant). Additional washing steps with 6 M urea were performed to denature bound enzyme molecules. Second, the peptide microarrays were treated with enzyme in presence of inhibitor (100 µM sodium orthovanadate), or in the presence of competing substrate (pTyr2). All control experiments showed that XopH and the C267A variant did not bind to immobilized phosphopeptides under the conditions used. Thus, any epitope-masking effect can be excluded.

### Protein phosphatase assay on peptide microarrays

XopH and XopH_C267A_ were incubated at room temperature (RT) with the peptide microarray using the TECAN Hybstation HS400. Prior to phosphatase treatment, two wash steps with phosphate-buffered saline (pH 7.5) with 0.1% Tween20 (PBST) and one wash step with PBS buffer were applied. Microarray blocking was performed with PBST and 3% bovine serum albumin (BSA) for 10 min, followed by 2× PBST and 1× PBS wash steps. Peptide microarrays were treated for 2 h with 10 µg mL^−1^ enzyme in reaction buffer (50 mM HEPES, pH 7.0, 100 mM NaCl, 1 mM dithiothreitol (DTT), 1 mM MgCl_2_ and 3% BSA). A control experiment without enzyme was done to obtain starting signal intensities for each spot. After washing with 5× PBST and 1× PBS buffer α-phospho-tyrosine mouse antibody (Cell Signaling Technology Europe, Leiden, the Netherlands) was applied at 10 µg mL^−1^ concentration in PBST + 3% BSA for 1 h followed by 5× PBST and 1× PBS wash steps. For staining the phosphopeptide-bound antibody Dylight649-labeled anti-mouse IgG (Pierce, Thermo Fisher Scientific) was used (30 min at 1 µg mL^−1^ in PBST, 3% BSA). Final washing steps were carried out with 5× PBST, 1× PBS, 2× distilled H_2_O and 2× nitrogen drying. To check for XopH binding to immobilized phosphopeptides, we applied α-XopH-antibody (1:5000 diluted, 1 h), followed by 5× PBST, 1× PBS washing steps and Alexa Fluor 532-labeled α-rabbit IgG (Thermo Fisher Scientific) at 1 µg mL^−1^ concentration for 30 min followed by 5× PBST, 1× PBS, 2× dH_2_O and 2× nitrogen drying. To determine if the signal decrease is caused by enzymatic action, we performed control experiments with inhibited phosphatase (0.1 mM sodium orthovanadate (Sigma-Aldrich)) or in the presence of excess of competing substrate (600 µM pTyr2-peptide (DADE(pY)LIPQQG) using similar conditions for the microarray experiments. To remove bound phosphatase molecules from the immobilized phosphopeptides, we added two urea wash steps (20 min each) after XopH incubation (1 × 6 M urea and 1 × 3 M urea in dH_2_O, pH 7.4) followed by 3 × dH_2_O wash steps. Fluorescence imaging of peptide microarrays was done using an Axon GenePix 4000B scanner at appropriate wavelengths, resolution 10 µm per pixel. Image evaluation was performed with GenePix Pro 7.2 (www.moleculardevices.com) followed by statistical analyses to identify top substrates and create two-sample logos (*t* test was chosen as statistical test and amino acid residue was shown if *p* value was below 0.05; http://www.twosamplelogo.org/cgi-bin/tsl/tsl.cgi).

### High-performance liquid chromatography kinetics

Phosphorylated peptides (pTyr2: Ac-DADE-pY-LIPQQGW-NH_2_, pTyr-chip: Ac-KVDVDE-pY-DENKFVW-NH_2_ and negative control Ac-GRKKIK-pY-KSLTRNW-NH_2_) were purchased from JPT Peptide Technologies (Berlin, Germany). Phosphopeptides were dissolved in 50 mM HEPES, pH 7.0, 10 mM NaCl, 1 mM DTT, 1 mM MgCl_2_ at 0.5–50 µM at 37 °C and reaction was started by addition of 0.05 mg mL^−1^ XopH. Reactions were stopped at different time points (1–120 min) by addition of 10% (v/v) trifluoroacetic acid (TFA) to a final concentration of 1% TFA (v/v). Reactions were analyzed using an Agilent 1100 series HPLC and Kinetex 2.6 µm XB-C18 100 A, 50 × 3.0 mm column for separation. Peak areas of phosphorylated and dephosphorylated peptides at different time points were used to determine the velocity of enzymatic reaction. Nonlinear regression according to Michaelis-Menten model and calculation of *K*_M_ and *k*_cat_ was performed using GraphPad Prism 5.01 software (www.graphpad.com).

### Chemical synthesis and analysis of pure InsP_6_

^1^H-NMR spectra were recorded on a Bruker 400 MHz spectrometer at 298 K in the indicated deuterated solvent. Data are reported as follows: chemical shift (δ, ppm), multiplicity (s, singulet; d, doublet; t, triplet; q, quartet; m, multiplet, or not resolved signal; br, broad signal), coupling constant(s) (J, Hz), integration. All signals were referenced to the internal solvent signal as standard (CDCl_3_, δ 7.26; D_2_O, δ 4.79; CD_3_OD, δ 3.31; DMSO-d6, δ 2.50). ^31^P[^1^H]-NMR spectra and ^31^P-NMR spectra were recorded with ^1^H-decoupling or ^1^H coupling on Bruker 162 MHz or 202 MHz spectrometers at 298 K in the indicated deuterated solvent. All signals were referenced to an internal standard (PPP). ^13^C[^1^H]-NMR spectra were recorded with ^1^H-decoupling on Bruker 101 or 125 MHz spectrometers at 298 K in the indicated deuterated solvent. All signals were referenced to the internal solvent signal as standard (CDCl_3_, δ 77.0; CD_3_OD, δ 49.0; DMSO-d6, δ 39.5). Mass spectra were recorded on Finnigan MAT95 MS, Bruker Esquire LC MS, Bruker maXis QToF HRMS and Finnigan TSQ700 MS machines. The chemical synthesis is outlined in Supplementary Fig. 5a, analytical data in Supplementary Fig. 5b–d. Synthesis was carried out as follows:

Synthesis of protected hexakisphosphate **2**: 50.0 mg (0.277 mmol, 1.00 eq.) of *myo*-inositol (**1**) and 2.27 g (4.49 mmol, 16.0 eq.) of 9-fluorenylmethyl phosphoramidite (Fm-PA) were coevaporated with dry MeCN (3 mL). The residue was dissolved in dry DMF (5 mL). To this solution, 654 mg (5.54 mmol, 20.0 eq.) of 4,5-dicyanoimidazole were added. Progress of the reaction was monitored by ^31^P-NMR. After completion of the reaction (30–45 min), oxidation was achieved by slow addition of 763 mg (4.49 mmol, 16.0 eq.) *m*CPBA (70%, moistened with water) at 0 °C. The mixture was concentrated in vacuo and the product was crystalized from MeOH (5 mL) and purified by flash chromatography (CH_2_Cl_2_:MeOH; 10:0.1 to 10:1) yielding 245 mg of **2** as a white solid (0.088 mmol, 32%). TLC (CH_2_Cl_2_:MeOH; 10:0.1 v/v): *R*_f_ = 0.50; ^1^H-NMR (400 MHz, CDCl_3_): δ 7.78–7.52 (m, 30 H), 7.52–7.39 (m, 14 H), 7.39–7.00 (m, 52 H), 4.76–4.19 (m, 28 H), 4.17–3.81 (m, 14 H); ^13^C-NMR (126 MHz, CDCl_3_): δ 143.45–142.89 (m), 141.50–141.21 (m), 127.80–126.92 (m), 125.83–125.23 (m), 120.01–119.76 (m), 77.55, 77.24, 76.92, 74.83, 73.50, 70.07, 69.99, 69.53, 60.47, 47.94, 47.78, 21.14, 14.33; ^31^P{^1^H}-NMR (162 MHz, CDCl_3_) δ 1.09 (s), 0.35 (s), −0.02 (s), −1.69 (s); ^31^P-NMR (162 MHz, CDCl_3_): δ 1.37–0.81 (m), 0.53–0.15 (m), 0.14 – −0.17 (m), −1.40 – −2.02 (m); HRMS (ESI) [M + 2Na]^2+^ calcd for C_174_H_138_Na_2_O_24_P_6_, 1421.8911; found, 1421.8933.

Synthesis of InsP_6_: 40.0 mg (14.3 μmol, 1.00 eq.) of **2** were dissolved in DMF (3 mL) and piperidine (0.5 mL) was added. The solution was stirred for 1 h at RT. After completion of the deprotection, the solution was concentrated and the product was precipitated with 10 mL Et_2_O. The precipitate was isolated by centrifugation, dissolved in MeOH, and crystallized by addition of Et_2_O. Centrifugation was repeated and crystals were dried in vacuo. Piperidinium counter ions were exchanged to sodium ions by addition of excess NaI to a MeOH solution of the piperidinium salt of **3**. After 30 min of stirring, the pure sodium salt of **3** precipitated, evidenced by the absence of proton resonances of piperidinium ions in the ^1^H-NMR spectrum. The purity was confirmed by ion chromatography as described in the next section. Yield: 12.2 mg of **3** (13.2 μmol, 92%). ^1^H-NMR (400 MHz, D_2_O): 4.86 (dd, *J* = 10.1 Hz, *J = *10.1 Hz, 1 H), 4.44 (ddd, *J* = 9.5 Hz, *J* = 9.5 Hz, *J* = 9.5 Hz, 2 H), 4.25–4.13 (m, 3 H); ^13^C-NMR (101 MHz, D_2_O): δ 77.44, 75.99, 75.38, 73.26; ^31^P{^1^H}-NMR (162 MHz, D_2_O): δ 2.34 (s), 1.87 (s), 1.36 (s), 0.89 (s); ^31^P-NMR (162 MHz, D_2_O): δ 2.34 (d, *J* = 9.6 Hz), 1.87 (d, *J* = 9.5 Hz), 1.36 (d, *J* = 9.2 Hz), 0.89 (d, *J* = 9.8 Hz); HRMS (ESI) [M-12Na^+^ + 10 H^+^]^2^^−^ calculated for C_6_H_16_O_24_P_6_, 328.9234; measured, 328.9234.

### Phytase assay

InsP_6_ was purchased from Sichem (Bremen, Germany) or synthesized as outlined above. For initial experiments, also InsP_6_ from Sigma-Aldrich was used. InsP_5_ isomers were from Sichem. Reaction mixtures (50 µL) of 0.05 µg µL XopH::His_6_, 0.01 mM InsP_6_, 50 mM HEPES (pH 7.0), 5% glycerol and 0.1% ß-mercaptoethanol were prepared in 96-well plates. Salt concentration varied from 10 to 1000 mM NaCl. The concentration of tested metal ions (MgCl_2_, CaCl_2_, MnCl_2_, ZnCl_2_, and KCl) and EDTA was 1 mM. For pH profiling, the Wizard pH buffer screen from Rigaku (Bainbridge Island, USA) was used. The concentration of the alternative phytase substrates glucose 6-phosphate, glycerophosphate, and fructose 1,6-bisphosphate (Sigma-Aldrich) was 10 µM. The enzymatic reaction was started by adding substrate and stopped after 1 h incubation at 37 °C by adding 50 µL Molybdate Dye/Additive mixture (Promega). After 5 min, the reaction was analyzed at 620 nm in a Spectrostar Nano microplate reader (BMG Labtech). Phosphate standard was prepared according to the manufacturer's protocol (Promega, Wisconsin, USA). The extinction was corrected for substrate in buffer. Protein phosphatase activity is reported as release of inorganic phosphate (P_i_) per minute per µg protein. All experiments were done at least twice with similar results, using two independent protein preparations each. The enzyme kinetics were analyzed in the range of 0–60 µM InsP_6_. Data were fitted to Michaelis–Menten equation using KaleidaGraph4.0.

### PAGE and purification of the XopH product from the gel

For PAGE, 10 nmol InsP_6_ were incubated with 0.13 µg µL^−1^ XopH for 1 h at 28 °C in 15 µL reaction buffer composed of 50 mM HEPES (pH 7.0), 10 mM NaCl, 5% glycerol, and 0.1% ß-mercaptoethanol. XopH reaction products were separated using PAGE and purified by two dehydration–hydration cycles^61^. Unprocessed gel images are provided in Supplementary Fig. 13.

### MALDI MS

XopH (0.13 µg µL^−1^) digests of InsP_6_ (100 nmol) in 150 µL buffer (50 mM HEPES (pH 7.0), 10 mM NaCl, 5% glycerol and 0.1% ß-mercaptoethanol) for 1 h at 28 °C were purified by PAGE as described above. XopH reaction products were then analyzed by MALDI-ToF-MS (9-aminoacridine matrix)^61^.

### NMR spectroscopy

Spectra were recorded on a Bruker Avance III HD 600 equipped with a BOSS-3 shim system, a digital lock control unit, a BCU II cooling unit, a GAB gradient spectroscopy and 5 mm BB observe probe (1H; 19F−109Ag; 564.7–27.9 MHz). The samples were held at 300 K during the measurements (NS: 8–16k, AQ: 0.34 s, DW: 5.2 us, D1: 2 s)

InsP_5_ [1-OH] and InsP_5_ [3-OH] as decasodium salts (1 mg batches, dissolved as a stock solution in 100 μL D_2_O) were from Sichem. Enantiopure L-arginine amide dihydrochloride (L-Arg-N) was purchased from Sigma-Aldrich.

XopH (0.13 µg µL^−1^) digests of InsP_6_ (600 nmol) in buffer (150 mM ammonium acetate, pH 7.1) were concentrated to a volume of ca. 300 µL and then diluted with D_2_O to enable locking (total volume 600 µL). Residual XopH activity was destroyed by heating to 95 °C for 15 min. Spiking of the sample was performed with commercial InsP_5_ in different ratios (1.5:1, 1:1, and 1:1.5) in the presence of L-Arg-N (100- to 150-fold excess, adjusted during the spiking experiments).

### HPIC analysis

For high-performance ion chromatography (HPIC), two different devices were used. For the data presented in Fig. 4c, a Thermo Scientific Dionex ICS-5000^+^ chromatograph using a dual pump equipped with a 10 µL injector loop, a Dionex CarboPac PA100 guard column (4 × 50 mm) and Dionex CarboPac PA100 analytical column (4 × 250 mm) was used to separate the *myo*-inositol phosphates. They were detected after post-column reaction in a reactor coil with 1% Fe(NO_3_)_3_ ∙ 9 H_2_O in 0.33 M HClO_4_ according to the method of Phillippy and Bland^62^ (Flow rate 0.4 mL min^−1^), with UV detection (290 nm). Samples were prepared in DI water with a concentration of 500 mg L^−1^ and 10 µL were injected. A gradient of A) 0.5 M HCl and B) H_2_O (18 MΩ * cm); 0–8 min, 5–10% A, 95–90% B; 8–25 min, 10–35% A, 90–65% B; 25–35 min, 35–100% A, 65–0% B; 35–42 min, 100% A, 0% B; 42.1–50 min, 100–5%, 0–95% B (Flow rate 1 mL min^−1^) was used to separate the analytes.

For the measurements presented in Supplementary Fig. 3e, InsP_x_ were incubated for 8 h at 28 °C in ammonium acetate buffer (150 mM ammonium acetate, adjusted with NH_3_ to pH 7). Samples were appropriately diluted and chromatographed on an HPLC system using a Pharmacia HR5/5 (5 cm) column and a gradient of HCl (0.005–0.5 M; flow rate of 1.0 mL min^−1^). To achieve post-column derivatization, the eluents were mixed with 0.1 M HCl containing 5.18 mM FeCl_3_ and 0.25 M NaCl via the twisted PTFE coil (length 8 m; i.d. 0.5 mm) (HPLC pump 2248, Pharmacia; flow rate of 0.8 mL min^−1^). A series of InsP_x_ standards was used to confirm the identity of the degradation products.

### Extraction and HPLC analyses of yeast and *N. benthamiana*

Inositol polyphosphate measurements were carried out with a *ddp1*∆ knockout yeast strain (BY4741; *MATa his3Δ leu2Δ met15Δ ura3Δ YOR163w::kanMX4*) to better visualize changes in *myo*-inositol pyrophosphates. The strain was generated by the *Saccharomyces* genome deletion project (http://www-sequence.stanford.edu/group/yeast_deletion_project/deletions3.html) and obtained from Open Biosystems (GE Healthcare, Munich, Germany). Yeast transformants were grown to midlog phase in glucose-free minimal media supplemented with 3% raffinose and 6 µCi mL^−1^ [^3^H]-*myo*-inositol (30–80 Ci mmol^−1^; Biotrend; ART-0261-5), then induced for 3 h in minimal media containing 3% raffinose and 2% galactose. Cells were harvested, extracted, and analyzed by Partisphere SAX HPLC^63^. Inositol polyphosphate analyses with extracts of *N. benthamiana* transgenic lines were done as follows. 10-day-old seedlings grown in MS 2% solid sterile media were transferred into 3 mL liquid 0.5 MS, 1% sucrose, pH 5.7 liquid media supplemented with 100 µCi [^3^H]-*myo*-inositol. The seedlings were labeled for a total of six days. In order to label adult leaves of *N. benthamiana* transgenic lines, leaves were cut from two-month-old plants grown in sterile conditions. The leaves were then kept in 3 mL liquid 0.5 MS, 1% sucrose, pH 5.7 liquid media supplemented with 100 µCi [^3^H]-*myo*-inositol for six days. After labeling, the leaves were thoroughly washed in ultrapure water for two times before freeze harvesting into liquid N_2_. Inositol polyphosphates were extracted from the plant materials and resolved by SAX HPLC with the gradient of buffers A (1 mM EDTA) and B [1 mM EDTA and 1.3 M (NH_4_)_2_HPO_4_, pH 3.8, with H_3_PO_4_]^29,63^. The experiments were repeated three times independently with similar results. Due to a change in the Partisphere SAX column between runs, slight alterations in the elution times of inositol phosphates were observed.

### XopH digestion of HPLC-purified [^3^H]-*myo*-inositol phosphates

[^3^H]-InsP_6_ and [^3^H]-InsP_5c_ were extracted and purified from SAX-HPLC runs of [^3^H]-*myo*-inositol-labeled *gfp*- and *xopH*-expressing *N. benthamiana* seedlings using an anion exchange-based desalting protocol^64^, and subjected to XopH digestion as described above. XopH was inactivated by heating the reaction mixture at 95 °C for 15 min.

### [^3^H]-*myo*-inositol labeling of inoculated tobacco leaves

Two dpi of *Agrobacterium* carrying indicated constructs, *N. benthamiana* leaves were cut into small pieces, placed in 6-well plates (Greiner) and washed two times with 5 mL of sterile ultra-pure water. The leaves were then transferred into 2 mL labeling media consisting of 0.5 MS, 0.25% sucrose, pH 5.7, 35 µCi mL^−1^ of [^3^H]-*myo*-inositol (30 to 80 Ci mmol^−1^; Biotrend; ART-0261-5). The labeling was allowed to proceed for five days before harvesting. Neutralized extracts of tobacco leaves were then resolved by SAX-HPLC with the gradient of buffers A (1 mM EDTA) and B [1 mM EDTA and 1.3 M (NH_4_)_2_HPO_4_, pH 3.8, with H_3_PO_4_]^29^.

### TiO_2_ bead pull down

Leaves of three different *C. annuum* cv. ECW or *N. benthamiana* plants were infected with *Xcv* as described above. Two dpi, three leaves per strain were harvested and frozen in liquid nitrogen. The samples were pooled and aliquoted to 130 mg each for further analysis. One aliquot was used for protein extraction with buffer containing 50 mM Tris-HCl, pH 7.5, 100 mM NaCl, 10 mM imidazole, 10% (v/v) glycerol, 0.1% (v/v) Tween 20 and 5 mM β-mercaptoethanol, and analyzed by SDS-PAGE.

Another aliquot was subjected to TiO_2_ bead enrichment. TiO_2_ beads (Titansphere, 5 µm, GL Sciences) were prepared following the protocol by Wilson et al.^65^. Frozen plant material was homogenized using a mixer mill MM 400 (Retsch), resuspended in 0.5 mL ice-cold perchloric acid and kept on ice for 10 min. After two centrifugation steps (20,000×*g*, 10 min, 4 °C) the supernatants were transferred into microfuge tubes containing 6 mg TiO_2_ beads each, mixed by brief vortexing and rotated (4 °C, 30 min). After centrifugation (6000×*g*, 1 min, 4 °C), the beads were washed twice with 0.5 mL ice-cold perchloric acid. Inositol polyphosphates were eluted by incubation with 0.2 mL of a 10% ammonia solution at RT for 5 min with intermediate brief vortexing. The beads were spun down (6000×*g*, 1 min) and supernatants removed into new tubes. The elution step was repeated yielding a final elution volume of 0.4 mL. Eluted samples were vacuum-dried, resuspended in 0.02 mL ultra-pure water and subjected to PAGE.

### Metabolite profiling

For LC-ToF-MS analysis of *myo*-inositolpolyphosphates in recombinant XopH enzyme assays, 20 nmol InsP_6_ were incubated with XopH for 1 h at 28 °C. Non-targeted ion detection by LC-MS was achieved using an Acquity UPLC (Waters) and a TripleTOF 5600 mass spectrometer using the software Analyst 1.6 TF (Sciex, Toronto, Canada). Inositol phosphates were separated on a Nucleoshell RP18 (150 mm × 2 mm × 2.7 µm, Macherey & Nagel, Düren, Germany) by ion pairing chromatography using solvent A: 10 mM tributyl amine (Sigma-Aldrich), which was acidified with glacial acetic acid to pH 6.2 and solvent B: acetonitrile. Gradient (% B): 0–2 min: 2, 18 min: 36, 21 min: 95, 22.5 min: 95, 22.52 min: 2, 24 min: 2, with a column flow of 400 µL min^−1^ and a column temperature of 40 °C.

Electrospray ionization was achieved in negative mode using a Duo-Source^TM^ (Sciex, Toronto, Canada). Source temperature: 600 °C, ion spray voltage: −4500 V, surtain gas: 35 psi, source gases ½: 60/60 psi. ToF-MS^1^-mass features were assayed between 65 and 1250 Da simultaneously with an array of non-targeted QToF-MS^2^ scan experiments in SWATH mode. During the latter, the transmission range for precursor ions in the Q1-quadrupol was set for 20 ms to mass windows of 33 Da and was incremented from *m/z* = 65–1250 Da. The declustering potential was kept at −35 V, the collision energy was ramped between −10, −45, and −60 V using the collision energy spread option. Mass accuracy (below 5 ppm) was automatically re-calibrated every 20th measurement using the calibrant delivery system, APCI ionization (Duo-Source^TM^) and the APCI calibrant mixture (all Sciex, Toronto, Canada). Simultaneous MS^1^-ToF scanning and SWATH-CID-fragmentation allowed for rapid assessment of MS^1^ and MS^2^ spectra with cycle times of 1 s. The measurements were performed twice with independent samples giving similar results.

### Data availability

The authors declare that all relevant data supporting the findings of this study are available within the paper and its supplementary information files or are available from the corresponding authors on request.

## Electronic supplementary material

Supplementary Information

Peer Review File
