# Supplementary material for: A 1-phytase type III effector interferes with plant hormone signaling
Source: Nat Commun. 2017 Dec 18;8:2159. doi: 10.1038/s41467-017-02195-8 (PMC5735085; doi:10.1038/s41467-017-02195-8)
Supplement: Supplementary file 1 — Supplementary Information [file 41467_2017_2195_MOESM1_ESM.pdf]

SUPPLEMENTARY INFORMATION

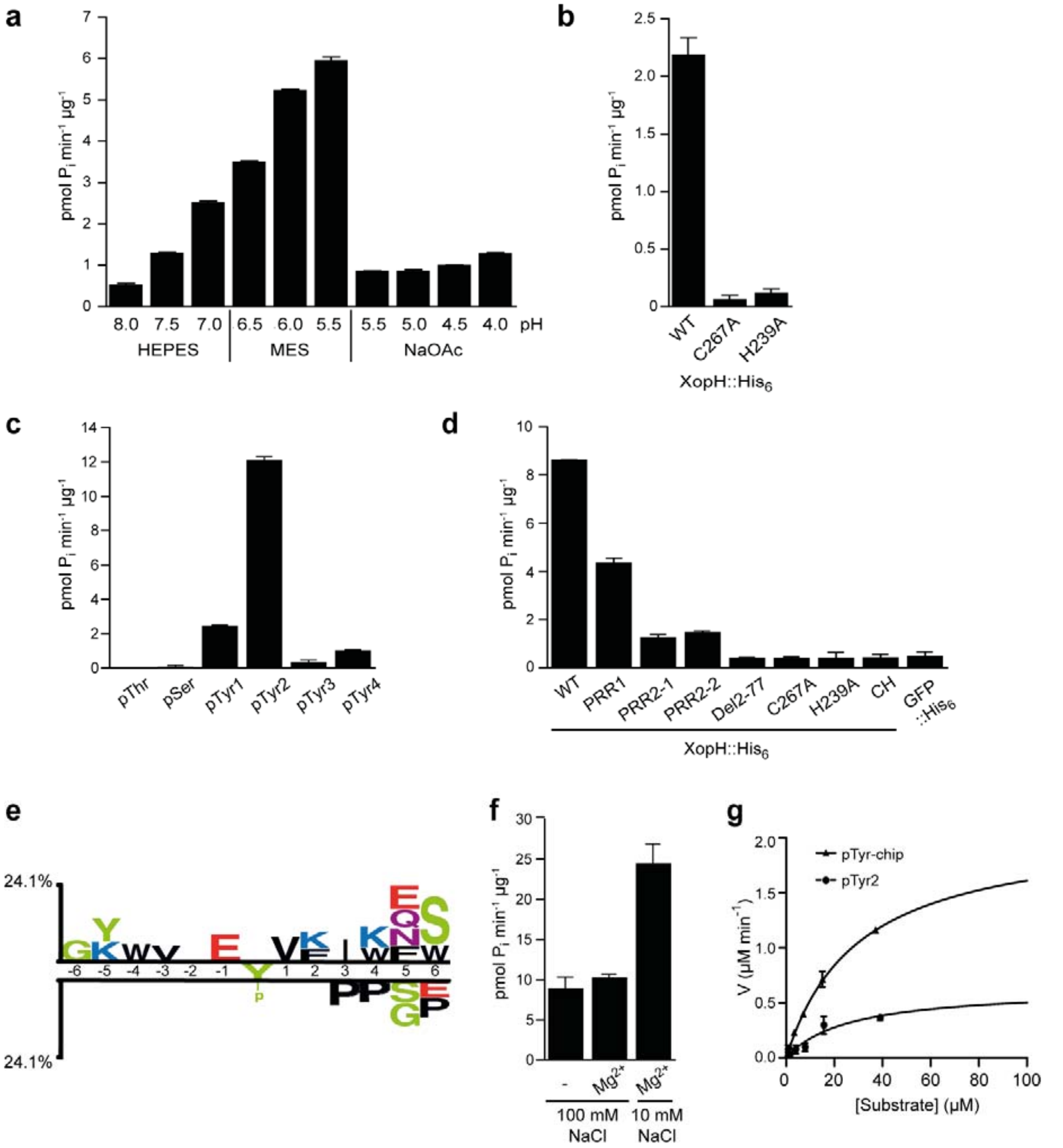

Supplementary Figure 1: Characterization of the XopH protein phosphatase activity.
a, Dephosphorylation of pNPP by XopH in different buffering systems. **b**, Dephosphorylation of pNPP by XopH (WT) and catalytic variants in 50 mM HEPES pH 7.0. **c**, Dephosphorylation of 0.5 mM commercial phosphor-peptides by XopH. pThr, PRApTVA; pSer, PRApSV; pTyr1,

ENDpYINASL; pTyr2, DADEpYLIPQQP; pTyr3, RRLIEDAEpYAARG; pTyr4, TSTEPQpYQPGENL. **d**, pTyr dephosphorylation by XopH (WT) and mutant derivatives. GFP served as negative control. **e**, Two-sample logo of pTyr dephosphorylation by XopH displaying enriched and depleted amino acid residues surrounding the dephosphorylated tyrosine. The logo was created from 72 top substrates out of >6000 peptides tested. **f**, pTyr2 dephosphorylation by XopH under high and low salt conditions. **g**, Kinetics of pTyr2 and pTyr-chip (KVDVDEpYDENKFVW) dephosphorylation by XopH. The maximal velocity V_{max} and Michaelis constant K_M were 0.63 ± 0.16 µM min^{-1} and 25.7 ± 12.2 µM for pTyr2 (circles), 0.21 ± 0.12 µM min-1 and 29.7 ± 3.0 µM for pTyr-chip (triangles). The experiments in a-d, f and g were performed twice with similar results, using two independent protein preparations each. Values are means of two technical replicates. Error bars indicate s.d.

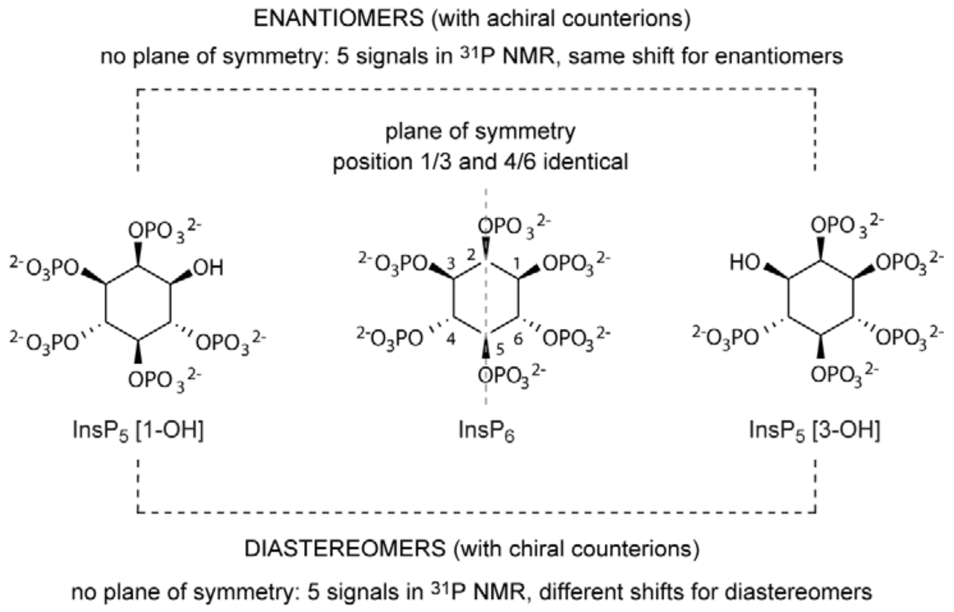

Supplementary Figure 2: Structures and symmetry of $InsP_6$ and dephosphorylated derivatives.

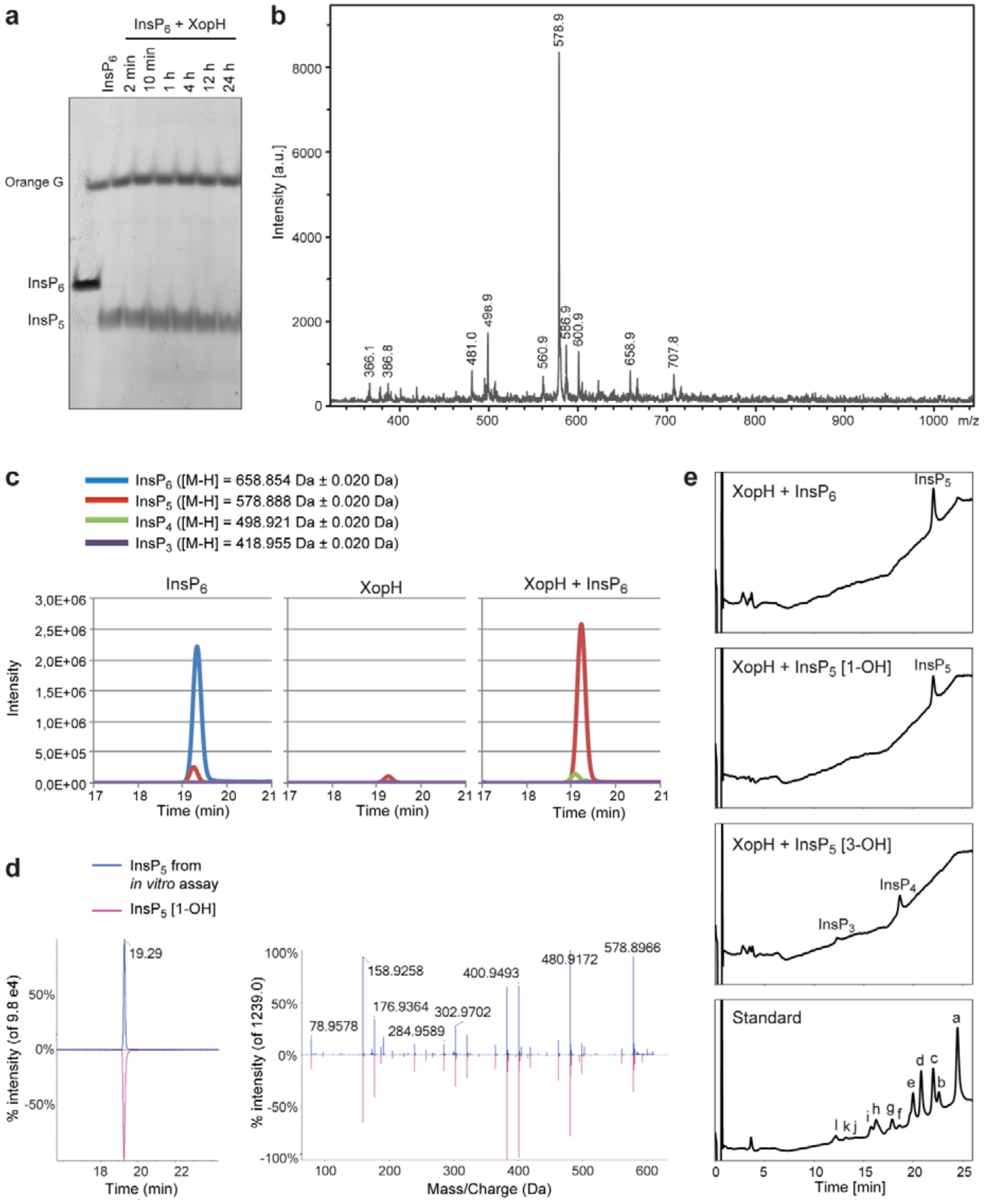

Supplementary Figure 3: $InsP_5$ is the main product of XopH-dependent $InsP_6$ dephosphorylation.

a, $InsP_6$ was incubated with XopH for various time points as indicated. Reaction products were separated by PAGE and visualized by toluidine blue. Undigested $InsP_6$ served as control. Orange

G, loading dye. The experiment was repeated once with similar results. **b**, MALDI-ToF-MS of gel-purified hydrolysis product of $InsP_6$ after treatment with recombinant XopH enzyme. The obtained ion mass (578.9) corresponds to an $InsP_5$. **c**, LC-ToF-MS analysis of *myo*-inositolpolyphosphates in recombinant XopH enzyme assays. Expected retention times: $InsP_6$: 19.33 min, $InsP_5$: 19.26 min (all isomers coelute in one peak), $InsP_4$: 19.08 min (all isomers coelute in one peak), $InsP_3$: 17.5-18.2 min (dependent on isomer). The experiment was repeated twice with similar results. **d**, LC-QToF-MS/MS analysis of $InsP_5$ [1-OH]. Left panel: chromatographic retention of $InsP_5$ monoisotopic precursor ion mass ([M-H] = 578.888 Da ± 0.02 Da), right panel: MS/MS spectra of $InsP_5$ from recombinant XopH enzyme assays and $InsP_5$ [1-OH] standard. **e**, High-performance ion chromatography (HPIC) analysis of XopH-dependent hydrolysis products of $InsP_6$, $InsP_5$ [1-OH] and $InsP_5$ [3-OH]. An $InsP_x$ mix served as standard: a, $InsP_6$; b, Ins(1,3,4,5,6)P_5; c, Ins(1,2,4,5,6)P_5; d, Ins(1,2,3,4,5)P_5; e, Ins(1,2,3,4,6)P_5; f, Ins(1,2,5,6)P_4; g, Ins(1,3,4,5)P_4; h, Ins(1,2,4,5)P_4; i, Ins(1,2,3,4)P_4/Ins(1,3,4,6)P_4; j, Ins(1,2,3,5)P_4/Ins(1,2,4,6)P_4; k, Ins(4,5,6)P_3; l, Ins(1,5,6)P_3.

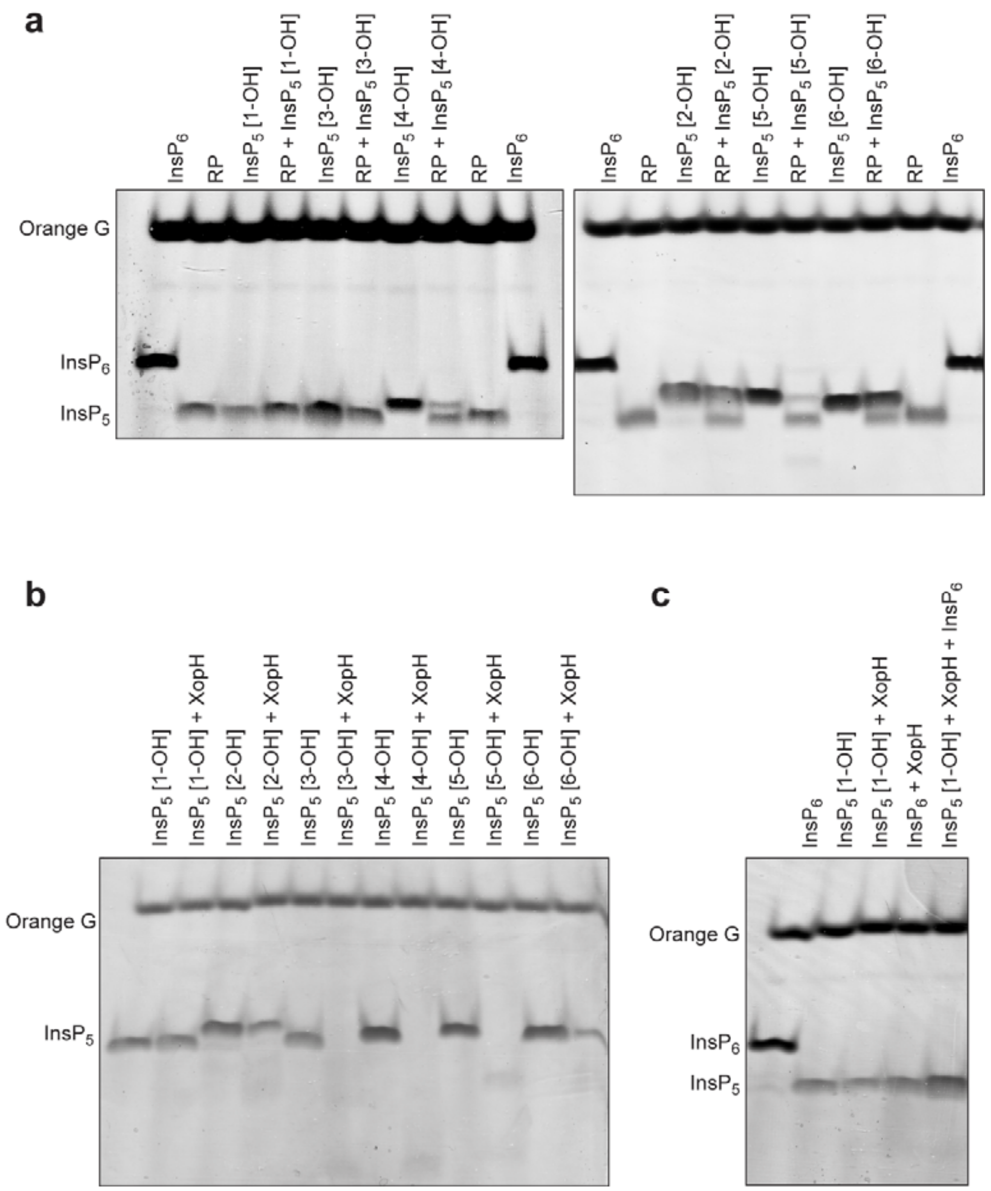

Supplementary Figure 4: $InsP_5$ [1-OH] is the likely XopH-dependent $InsP_6$ dephosphorylation product.

a, Reaction products (RP) obtained by phytate digestion with XopH (0.13µg/µL; 30 min) were mixed with $InsP_5$ isomers as indicated, separated by PAGE and visualized by toluidine blue staining. $InsP_6$ and $InsP_5$ isomers alone served as controls. Double bands report different isomer identities of RP and the respective $InsP_5$ species tested. **b**, All six different $InsP_5$ isomers (10 nmol) were digested with XopH, separated by PAGE and visualized by toluidine blue staining. **c**, An $InsP_5$ [1-OH]/XopH reaction mixture was incubated for 30 min at 28°C, then supplemented with $InsP_6$ (10 nmol) for additional 30 min, separated by PAGE and visualized by toluidine blue staining. $InsP_6$, $InsP_5$ [1-OH] and the $InsP_5$ [1-OH]/XopH reaction mixture (incubated for 1 h at

28°C prior to loading onto the gel) served as controls. The experiments were done twice with similar results.

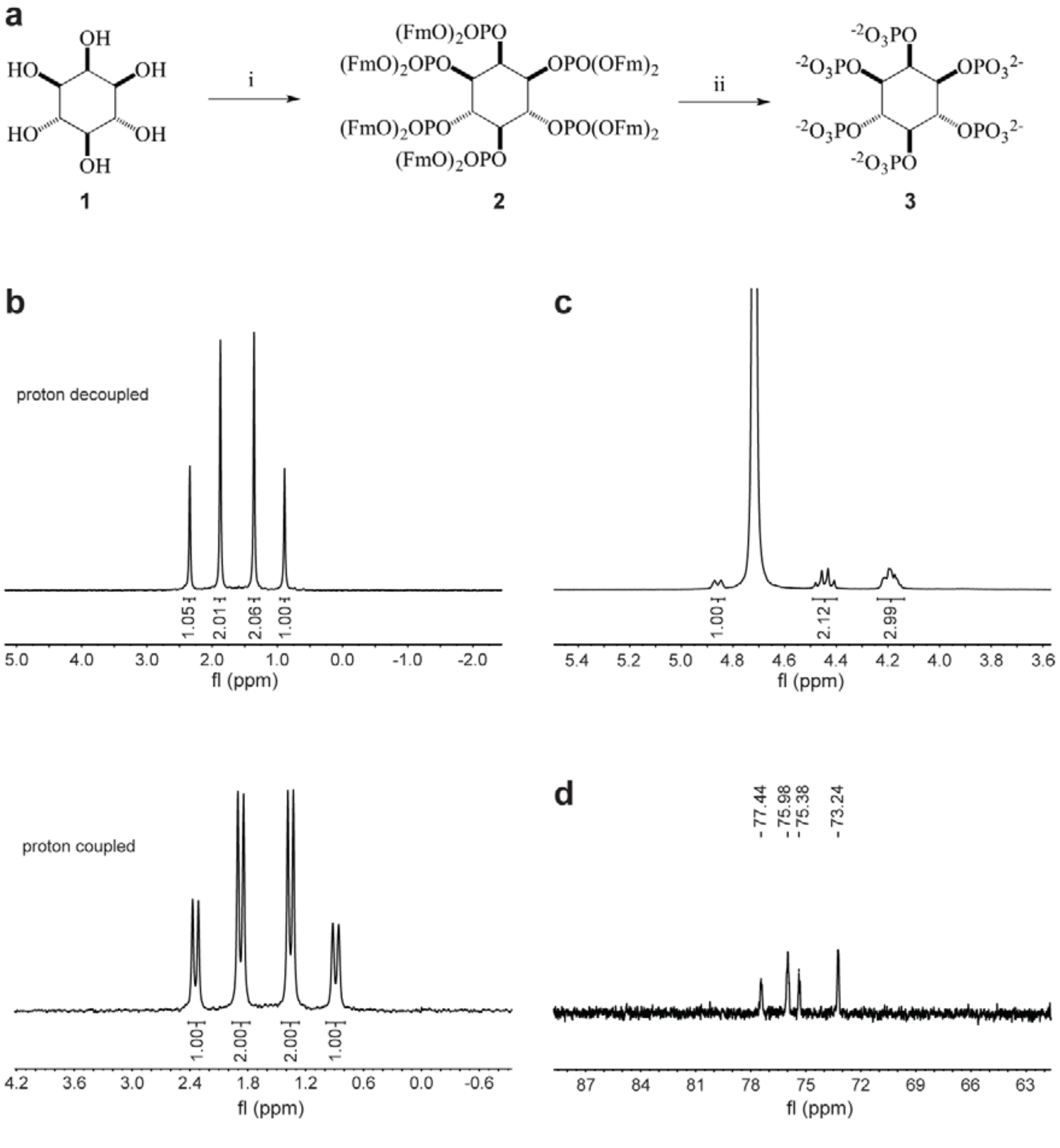

Supplementary Figure 5: $InsP_6$ synthesis and quality control.
a, Chemical synthesis of highly pure $InsP_6$: (i) Bis-fluorenylmethyl-di*iso*propylamino-P-amidite (CAS: 197709-11-8), 4,5-Dicyanoimidazole, then *m*CPBA, 32 % yield; (ii) 5% Piperidine in DMF, then precipitation (and ion exchange with NaI), 92% yield. **b-d**, Quality control of synthetic $InsP_6$. **b**, $InsP_6$, ^{31}P-NMR in D_2O. **c**, $InsP_6$, ^{1}H-NMR in D_2O. **d**, $InsP_6$, ^{13}C NMR in D_2O.

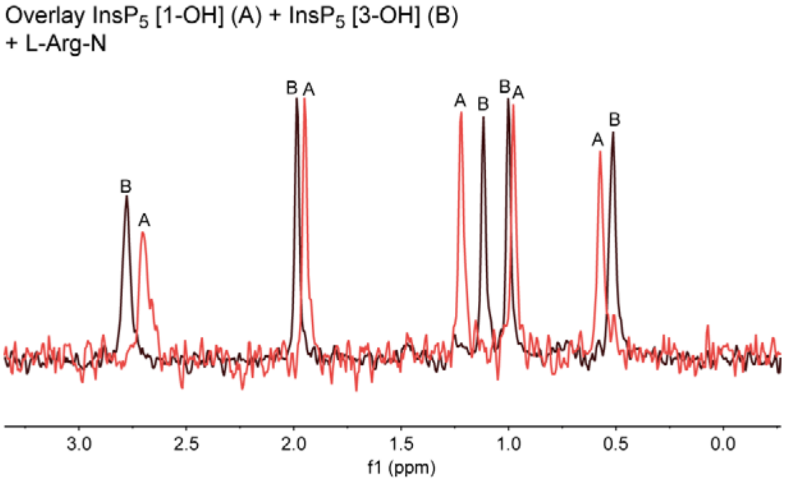

Supplementary Figure 6: L-Arg-N enables the discrimination of $InsP_5$ [1/3-OH] by ^{31}P-NMR.

Overlay of separately recorded mixtures of either $InsP_5$ [1-OH] (A) or $InsP_5$ [3-OH] (B), 100 µg each, in ammonium acetate buffer in the presence of excess of L-Arg-N (ca. 100 fold). The relative position of the peaks is identical to those found in spiking experiments.

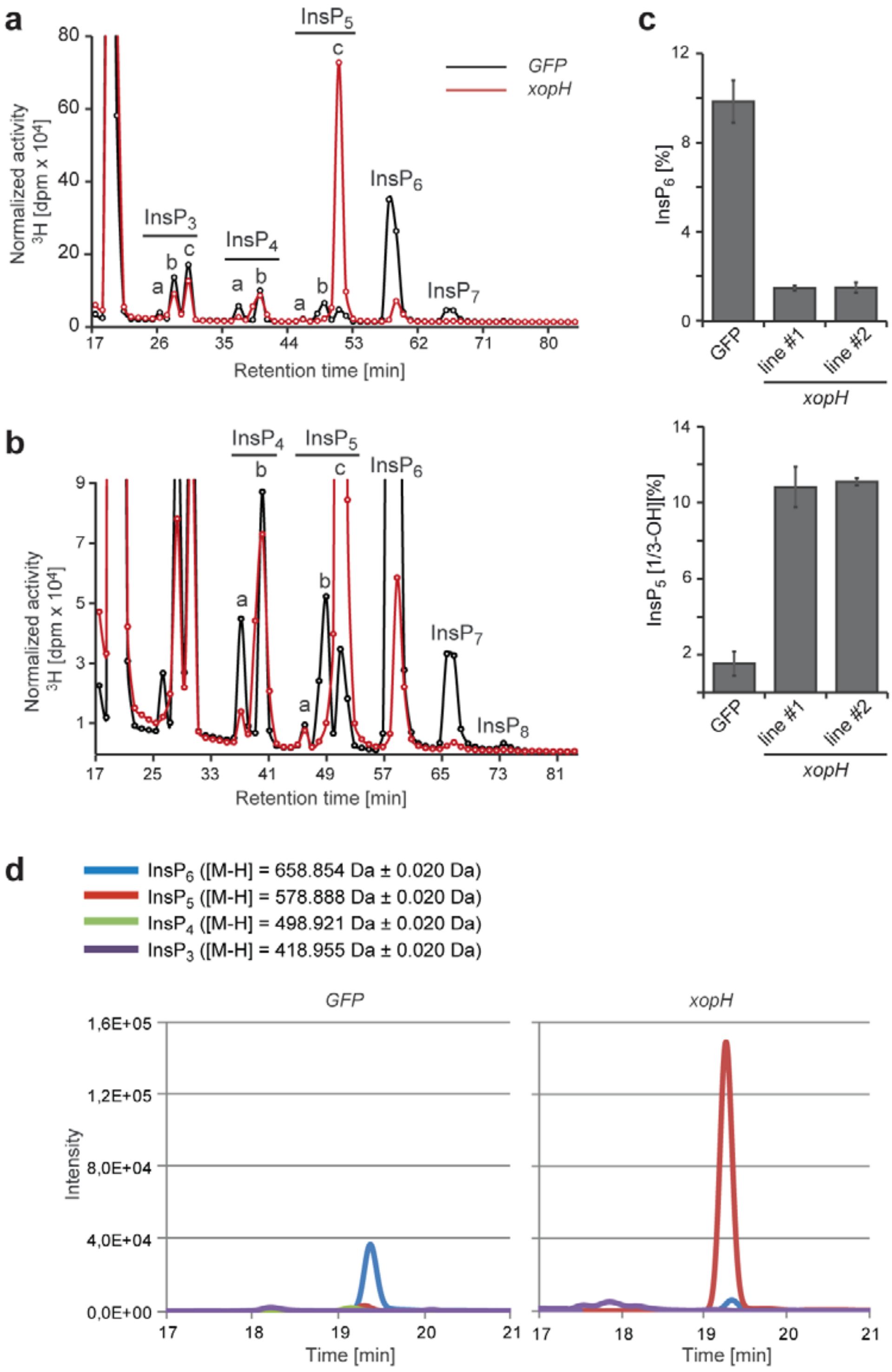

Supplementary Figure 7: XopH leads to $InsP_5$ accumulation *in planta*.

a, HPLC profiles of neutralized extracts from 16-day old *N. benthamiana* transgenic seedlings labelled with [^{3}H]-*myo*-inositol. Based on published chromatographic mobilities[1], $InsP_{5a}$ represents $InsP_5$ [2-OH] and $InsP_{5c}$ represents $InsP_5$ [1-OH] or its enantiomer $InsP_5$ [3-OH]. The

isomeric nature of $InsP_{3a\text{-}c}$, $InsP_{4a\text{-}b}$, $InsP_7$ and $InsP_8$ is unknown. **b**, Zoom-in into the HPLC profile. **c**, Relative amounts of $InsP_{5c}$ and $InsP_6$ in control line expressing *gfp* and two independent *xopH*-expressing lines. Error bars indicate s.e.m. **d**, LC-ToF-MS analysis of *myo*-inositolpolyphosphates in transgenic *N. benthamiana* seedlings. Plant extracts were generated as in **a**, but with unlabeled *myo*-inositol. Expected retention times: $InsP_6$: 19.33 min, $InsP_5$: 19.26 min (all isomers coelute in one peak), $InsP_4$: 19.08 min (all isomers coelute in one peak), $InsP_3$: 17.5-18.2 min (isomer-dependent). The experiments were performed three times with similar results.

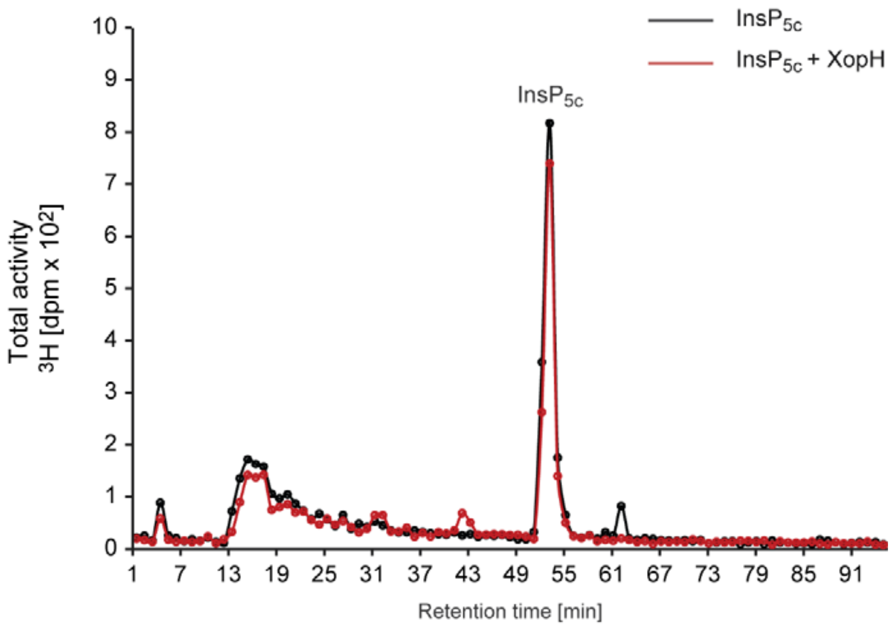

Supplementary Figure 8: The enantiomer identity of XopH-dependent $InsP_6$ hydrolysis *in planta* is $InsP_5$ [1-OH].

Digestion and HPLC analyses of plant-purified $InsP_{5c}$. $InsP_{5c}$ was purified from [^{3}H]-*myo*-inositol-labeled *xopH*- and *GFP*-expressing *N. benthamiana* seedlings (see methods). XopH-treated or non-treated $InsP_{5c}$ was then separated by SAX-HPLC. XopH was inactivated by incubating the reaction mixture at 95°C for 15 min. The figure shows the full chromatograms of the respective selections (min 45-71) depicted in Figure 7.

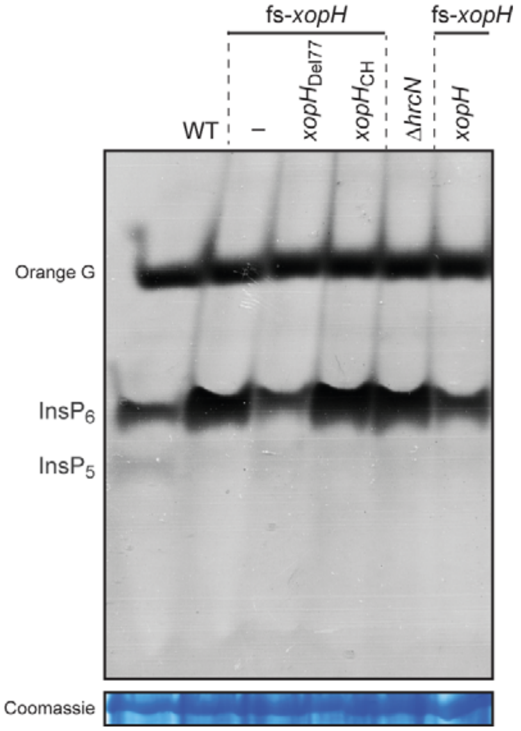

Supplementary Figure 9: XopH reduces the $InsP_6$ content of pepper leaves during *Xcv* infection.

$HClO_4$ extracts of *C. annuum* ECW leaves infected with *Xcv* strains as indicated were subjected to TiO_2 bead enrichment. Inositol polyphosphates were eluted from TiO_2 beads, resolved by PAGE and stained with toluidine blue. Protein extracts were visualized by Coomassie blue as a loading control. The experiment was repeated twice with similar results.

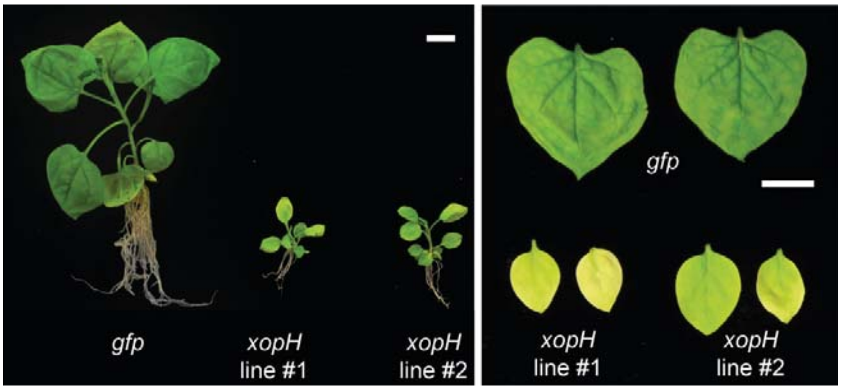

Supplementary Figure 10: XopH affects plant growth.
Photographs of two-months-old transgenic *N. benthamiana* plants grown on 0.5 MS + 1% sucrose. Scale bars, 1 cm.

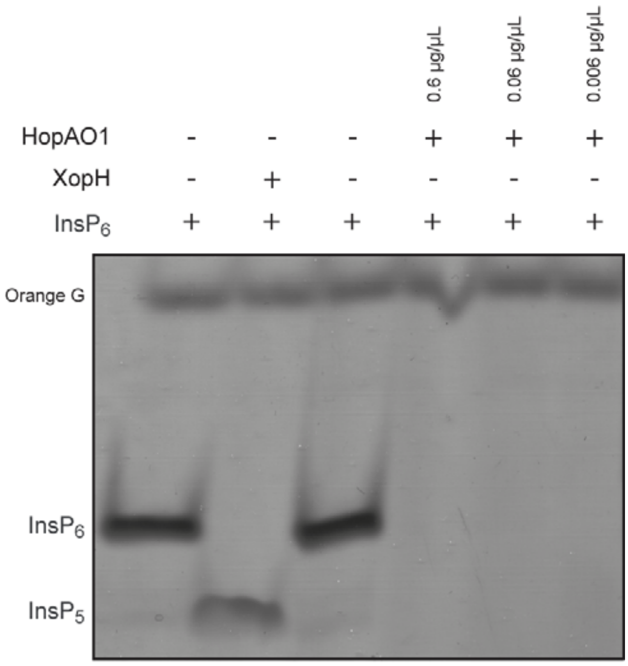

Supplementary Figure 11: HopAO1 from *Pseudomonas syringae* pv. *tomato* hydrolyzes $InsP_6$ into lower phosphorylated *myo*-inositol derivatives.
Recombinant XopH (0.13 µg/µL) or indicated amounts of recombinant HopAO1 were incubated with 10 nmol $InsP_6$ in reaction buffer (10 mM NaCl, 5% glycerol, 5 mM β-mercaptoethanol and 50 mM HEPES, pH 7.0) at 28°C for 30 min. Reaction products were separated by PAGE and visualized by toluidine blue staining. Orange G, loading dye. The experiment was repeated once with similar results.

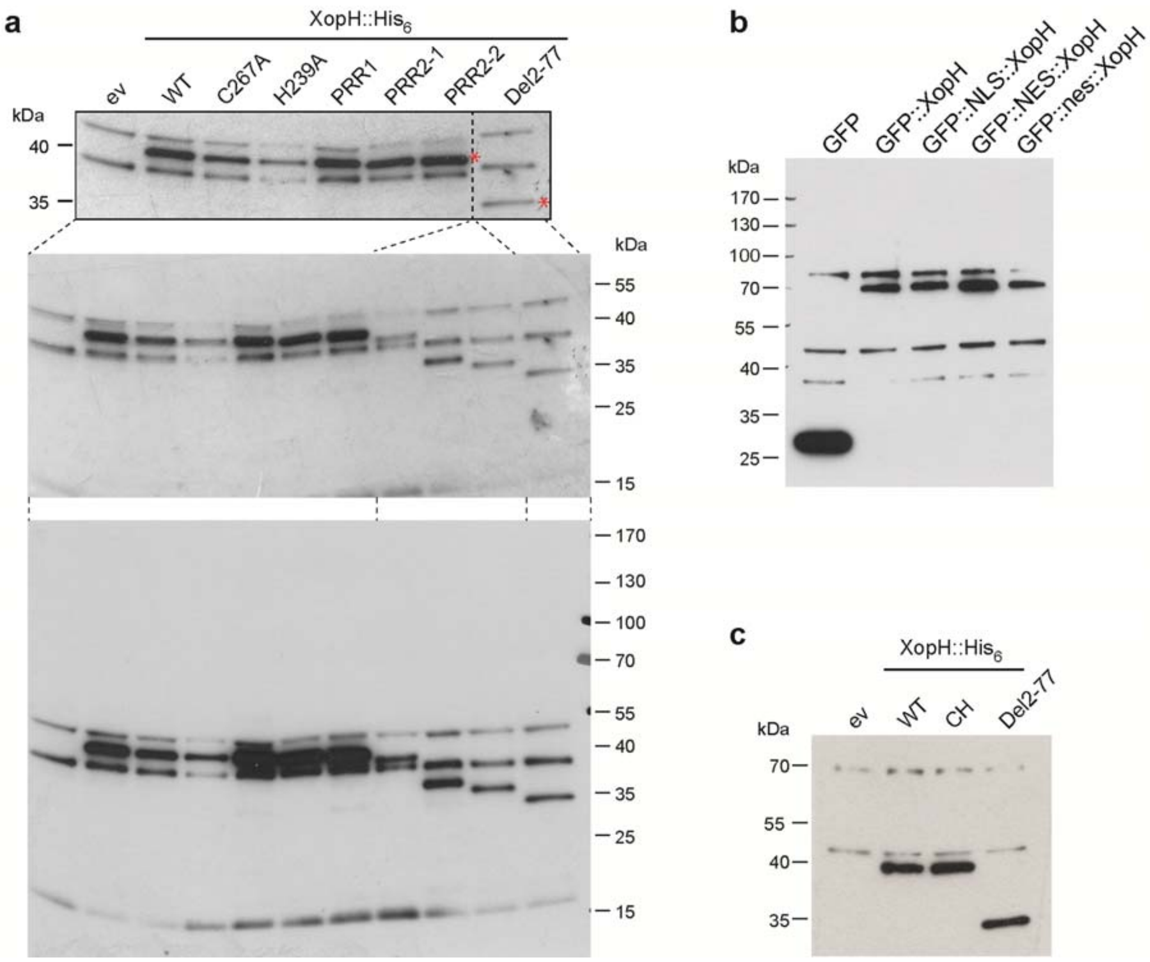

Supplementary Figure 12: Unprocessed Western blots.

a, Reproduction of Fig. 1e (*top*) and the full blot of the membrane including a longer exposure (*bottom*). Please note that we removed three lanes that were not relevant for the results presented (stippled lines). **b, c**, Full Western blots used in Fig. 3c (b) and Fig. 8b (c).

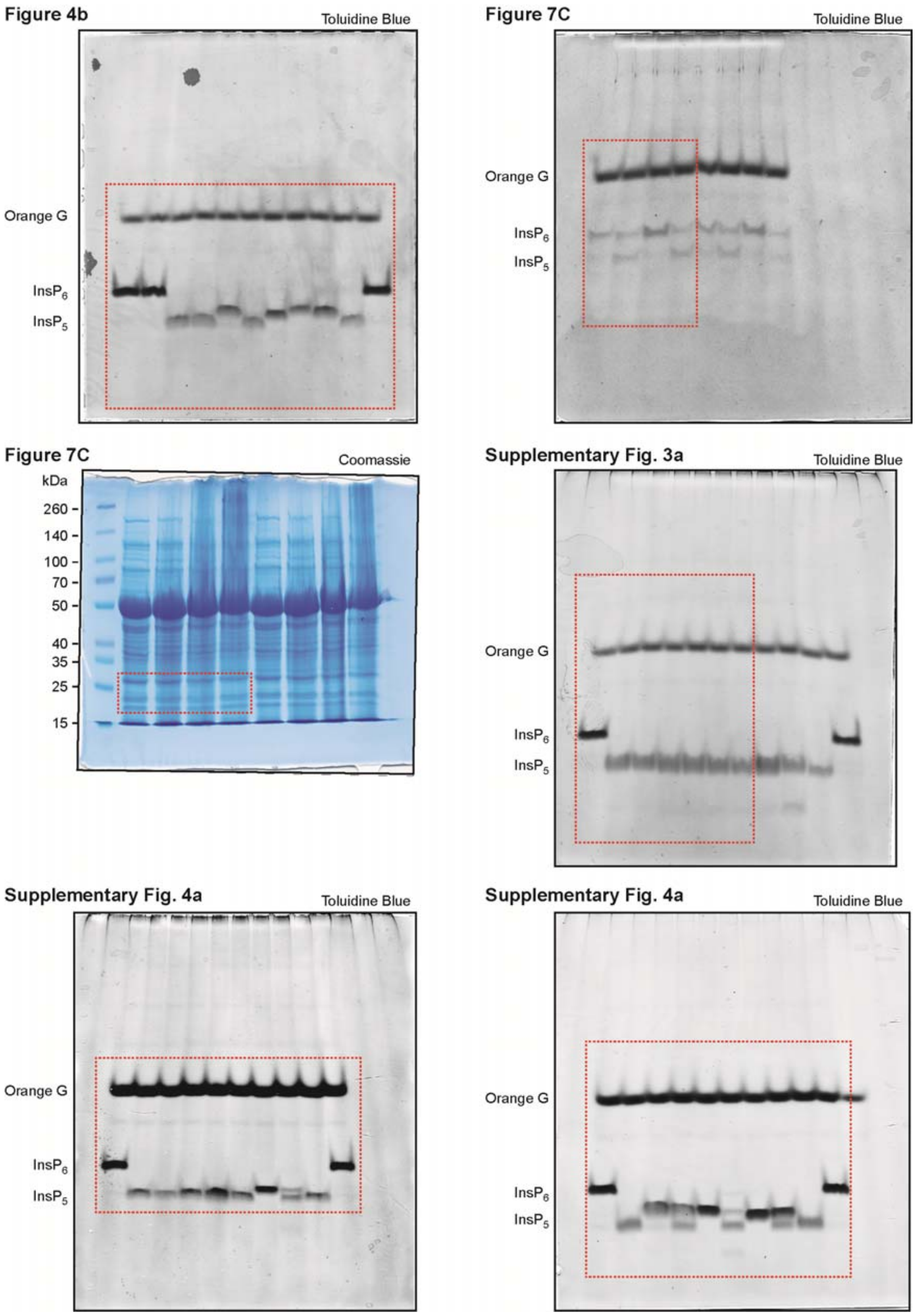

Supplementary Figure 13: Uncropped gels.

Supplementary Fig. 4b

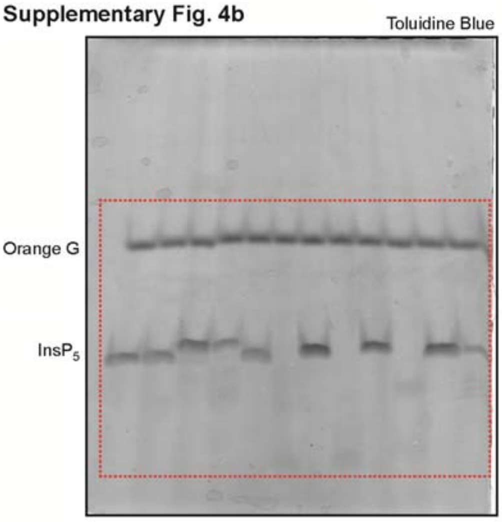

Supplementary Fig. 4c

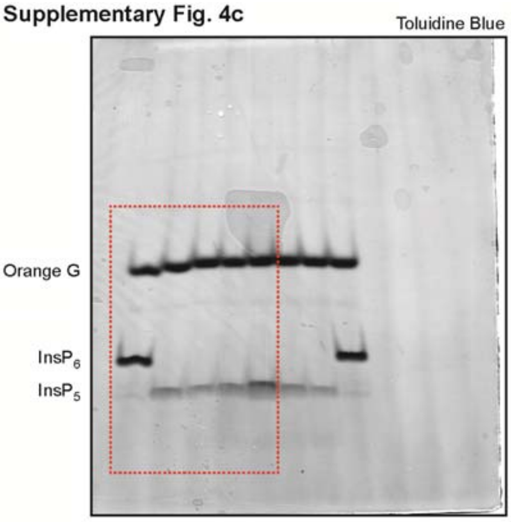

Supplementary Fig. 9

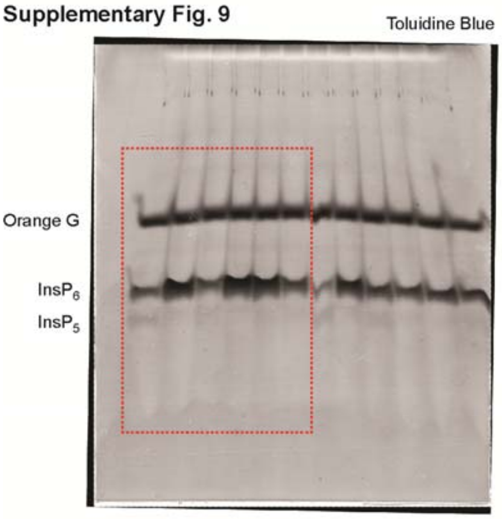

Supplementary Fig. 9

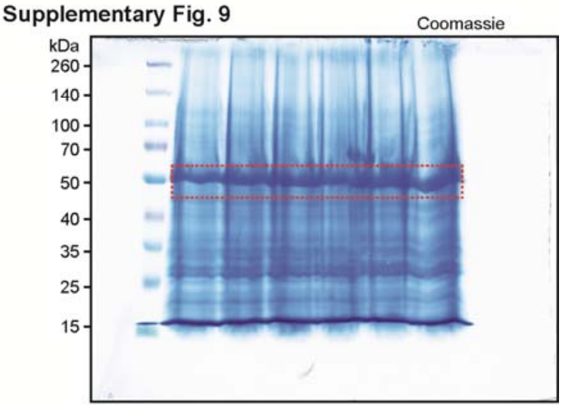

Supplementary Fig. 11

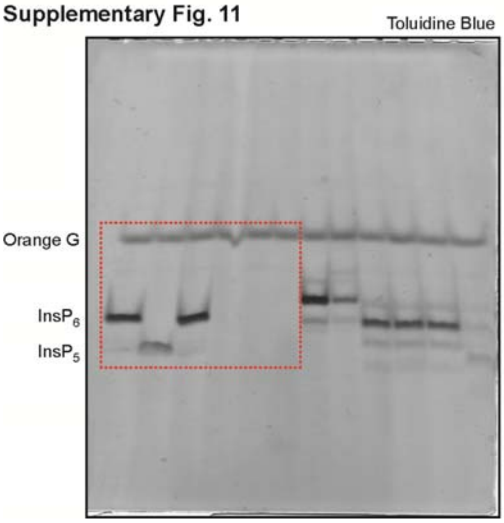

Supplementary Figure 13: Uncropped gels (continued).

Supplementary Table 1

Oligonucleotides used in this study.

Name	Sequence (5`-3`)	Purpose
XopH-GW-fw	CACCATGCCGAACAAAATCTCCGGCTCAA	GATEWAY cloning of *xopH-his*$_6$
XopH-del2-77 GW-fw	CACCATGTCACATCCTGTTCTAGCTTACGACAG	GATEWAY cloning of *xopH-del2-77-his*$_6$
XopH-C267A	TGCATGTACATGCTGGTATGGGCCT[a]	Site-directed mutagenesis (XopH-C267A)
XopH-262rev	GTCTCTCATCATGGGCCATCTCC[a]	
XopH-H239A-fw	GTTTGACAGTGACAGATGCTCTTTCACCACGGGCGGACGATATTG	Site-directed mutagenesis (XopH-H239A and -CH)
XopH-H239A-rv	CAATATCGTCCGCCCGTGGTGAAAGAGCATCTGTCACTGTCAAAC	
XopH-mutP43-52-53-fw	TCCCGCAGAACTCGCCGATCTAGCAAGCCGGCAAGCAGCTCGGTCAAAAACAGCACTTTA	Site-directed mutagenesis (PRR1 mutant)
XopH-mutP43-52-53-rv	TAAAGTGCTGTTTTTGACCGAGCTGCTTGCCGGCTTGCTAGATCGGCGAGTTCTGCGGGA	
XopH-mutP69-71-fw	TAATCCAGAAATTCAGAGACGCTTTGGCTCTCCCGCCACCACCCACGTCACATCCT	Site-directed mutagenesis (PRR2-1 mutant)
XopH-mutP69-71-rv	AGGATGTGACGTGGGTGGTGGCGGGAGAGCCAAAGCGTCTCTGAATTTCTGGATTA	
XopH-mutP73-74-75-76-fw	AGAAATTCAGAGACCCTTTGCCTCTCGCGGCAGCAGCCACGTCACATCCTGTTCTAGCTT	Site-directed mutagenesis (PRR2-2 mutant)
XopH-mutP73-74-75-76-rv	AAGCTAGAACAGGATGTGACGTGGCTGCTGCCGCGAGAGGCAAAGGGTCTCTGAATTTCT	Site-directed mutagenesis (PRR2-2 mutant)
caac-EGFP fw	CACCATGGTGAGCAAGGGCGAGGAGCT	SOE-PCR for *gfp-xopH* derivatives
NLS-XopH rv	TCCCGAGCCTCCAAAAAAGAAGAGAAAGGTCATGCCGAACAAAATCTCCGGCTCAA	SOE-PCR for *gfp-NLS-xopH-his*$_6$
NLS-XopH-fw	TTGAGCCGGAGATTTTGTTCGGCATGACCTTTCTCTTCTTTTTTGGAGGCTCGGGA	
NES-XopH-rv	TTGAGCCGGAGATTTTGTTCGGCATCTTGTTAATATCAAGTCCAGCCAACTTAAGAGCA	SOE-PCR for *gfp-NES-xopH-his*$_6$
NES-XopH-fw	TGCTCTTAAGTTGGCTGGACTTGATATTAACAAGATGCCGAACAAAATCTCCGGCTCAA	
nes-XopH-rv	TTGAGCCGGAGATTTTGTTCGGCATCTTGTTAGCATCTGCTCCAGCTGCCTTAAGAGCA	SOE-PCR for *gfp-nes-xopH-his*$_6$
nes-XopH-fw	TGCTCTTAAGGCAGCTGGAGCAGATGCTAACAAGATGCCGAACAAAATCTCCGGCTCAA	
XopH-His6 GW-rv	TTAGTGATGGTGATGGTGATGTGCATTGTGGTCGAGCCATTCGGAC	GATEWAY cloning of *xopH-his*$_6$, SOE-PCR *gfp-xopH* derivatives
eGFP-GW-rv	CTTGTACAGCTCGTCCATGCCGAGAGTGATCCCGGCGGCGGTCAC	GATEWAY cloning of *gfp*
eGFP-GW-fw	CACCATGGTGAGCAAGGGCGAGGAGCTGTTCACCGGGGTGGTGCCCATC	
pET-XopH-NdeI-fw	AAGGAGATATACATATGCCGAACAAAATCTCCGGC	In-Fusion HD cloning of *xopH- his*$_6$ into pET22b(+)
pET-XopH-del2-77-NdeI-fw	AAGGAGATATACATATGTCACATCCTGTTCTAGCTTACGAC	In-Fusion HD cloning of *xopH-del2-77-his*$_6$ into pET22b(+)
pET-XopH-XhoI-rv	GGTGGTGGTGCTCGAGTGCATTGTGGTCGAGCCATTCG	In-Fusion HD cloning into pET22b(+)
pUC57-BamHI-early-	TCTAGATATCGGATCCTACGCCAGCGTCGCTCT	pOK-early-stop-*xopH*

stop-fw		(fragment I)
xopH-early-stop-A-rv	CTTCCCGGATCGCGTCGCTGCT	
xopH-early-stop-B-fw	ACGCGATCCGGGAAGAGTGCCGACT	pOK-early-stop-*xopH* (fragment II)
pUC57-BamHI-early-stop-rv	GACGGGCCCGGGATCCACGTACATATTTGGCG CCCG	
si_CaCOI1_fwd	CACCAGATCTGCCACTTGATAATGGTGT[b]	COI1 silencing construct
si_NbCOI1_rev	TCCAGAAGGCCTTCATCGGAT	
si_CaEIN2_fwd	CACCAACGGGTACTTTCTGCTTC[b]	EIN2 silencing construct
si_CaEIN2_rev	CCAATACATAAGAATTTCGCAC	
si_CaEBF1_fwd	CACCAATAAGTGCTTGCAGTGGAG[b]	EBF1 silencing construct
si_CaEBF1_rev	GCAGTTCACCAGGGAAAGAG[b]	
NbMYC2_fwd	GATGGGATGCTATGATTCGTATAC[c]	qRT-PCR
NbMYC2_rev	CTGAAACACTAGCATGGTGCACATC[c]	
NbPI-II_fwd	GGGGAGCCTCAAAGTGCTGC[c]	qRT-PCR
NbPI-II_rev	CAGAGTTTAGCATGACAGTGC[c]	
NbPR1_fwd	CGTTACGGCGAAAACCTAGC	qRT-PCR
NbPR1_rev	CCCGGTGCACAAGTATTCGA	
NbPR4_fwd	GGCCAAGATTCCTGTGGTAGAT[d]	qRT-PCR
NbPR4_rev	CACTGTTGTTTGAGTTCCTGTTCCT[d]	
NbLR23_fwd	AGGATGCCGTCAAGAAGATGT	qRT-PCR
NbLR23_rev	TCTGGTGTCAATCGAACAAACG	
NbF-Box_fwd	CCTCCCAGGTATTTTCGTGCA	qRT-PCR
NbF-Box_rev	CCTTTGCCTTGGTTCCAGGA	
NbCOI1_fwd	ATGCACTTCTTGACACAGCAG	qRT-PCR
NbCOI1_rev	CCCCAACAACATTCCTTGTC	
NbEIN2_fwd	CCGTCTGTTGGTCGATCAAC	qRT-PCR
NbEIN2_rev	GGCGAATGTTCAAACCTCTG	
NbEBF1_fwd	ATGTCTAAAGTCTTCAATTTTAGTG	qRT-PCR
NbEBF1_rev	CAAGGGAGAGAAATAGGCTG	
XopH ATG fw	ATGCCGAACAAAATCTCCGG	Analysis of *xopH*-transgenic *N. benthamiana*
XopH rv-3	GTCACGTGATTTGCCAGCAT	

[a] 5´phosphorylation; [b] 1-bp exchange in *N. benthamiana*; [c] adapted for *N. benthamiana* from references 2 and 3.

Supplementary References

1 Stevenson-Paulik, J., Bastidas, R. J., Chiou, S.-T., Frye, R. A. & York, J. D. Generation of phytate-free seeds in Arabidopsis through disruption of inositol polyphosphate kinases. *Proc. Natl. Acad. Sci. USA* **102**, 12612-12617 (2005).

2 Shoji, T. & Hashimoto, T. Tobacco MYC2 regulates jasmonate-inducible nicotine biosynthesis genes directly and by way of the *NIC2*-locus *ERF* genes. *Plant Cell Physiol.* **52**, 1117-1130, doi:10.1093/pcp/pcr063 (2011).

3 Kiba, A. *et al.* SEC14 phospholipid transfer protein is involved in lipid signaling-mediated plant immune responses in *Nicotiana benthamiana*. *PLoS ONE* **9**, e98150, doi:10.1371/journal.pone.0098150 (2014).
