# Supplementary material for: A 1-phytase type III effector interferes with plant hormone signaling
Source: Nat Commun. 2017 Dec 18;8:2159. doi: 10.1038/s41467-017-02195-8 (PMC5735085; doi:10.1038/s41467-017-02195-8)
Supplement: Supplementary file 2 — Peer Review File [file 41467_2017_2195_MOESM2_ESM.pdf]

Reviewers' comments:

Reviewer #1 (Remarks to the Author):

Summary: This paper describes the biochemical activity of the Xanthomonas type three effector XopH, previously shown to exhibit dephosphorylation activity. The authors demonstrate that the effector can dephosphorylate phytate, a phosphate storage compound that is also involved in protecting plants from pathogens.

"Effector biology" or the sub field that aims to understand the molecular and biochemical function of T3Es is inherently interesting, important for translational biology and can be exceedingly difficult if interactions between pathogen proteins and host targets are not stable. In this work, the authors employ a novel NMR based approach to define XopH as a 1-phytase. Further, in planta data point towards a potential role for XopH in plant hormone pathways. Here, the authors took two approaches. First, transgenically expressing XopH in N. benthamiana plants resulted in stunted, chlorotic plants. Second, the authors transiently express XopH and measure defense gene expression with and without VIGS silencing of genes involved in hormone signaling. The main criticism here is simply that the biological relevance must be questioned. I do not believe that N. benthamiana is a natural host for this pathogen, the protein is being delivered not by Xanthomonas, but by an Agrobacterium T-DNA, and the host genes are silenced using VIGS, which would be expected to lead to patchy silencing. Nonetheless, the authors have assembled a nice dataset.

Suggestions/questions:
1. Unfortunately, I did not see the catalytically dead version of XopH included as a control for the N. benthamiana transgenics, perhaps because these experiments were initiated prior to the active domain being defined? Without this control, it is probably an overstatement to say that this phenotype is indicative of involvement in ethylene signaling since many things can lead to stunting and chlorosis.

2. Would it be possible to do any of the gene expression studies in the XopH expressing transgenic plants. For example, could the authors look at defense gene expression in these plants (we would expect to see high expression of PR1b and PR4), and potentially do VIGS on these plants to bolster the hormone connection? This may not be possible, given their developmental defect, but if so, this would strengthen this part of the paper.

Minor comments:
I think of the marker genes used in this study (especially PR1b) as general defense related genes. The authors should provide appropriate references if these genes have been conclusively tied to specific plant hormone pathways. Otherwise the text should be revised to reflect the conclusion that transient expression of XopH leads to higher defense related gene expression and that silencing EIN2 blocks the elevated expression of Pi-II.

Reviewer #2 (Remarks to the Author):

Summary

This reviewer (Charles Brearley) takes the view that the manuscript makes two notable discoveries, which, criticisms addressed, merit publication of the work in this journal.

The discoveries are:

XopH, a Type III effector, encodes a novel phytase (proven), but further confirmation of the identity of the IP5 product is needed. The character of this phytase appears highly novel.

Chiral shift reagents allow discrimination of enantiomers of inositol phosphate (pentakisphosphate). This requires further elaboration.

A third discovery: that the phytase has effect on inositol phosphate metabolism when introduced via Agrobacterium into plants, is proven. However, the identity of the inositol phosphate generated is undefined. It is likely that any phytase introduced by Agrobacterium would have effect on inositol phosphate metabolism. That expression of a phytase has effect on jasmonate- or auxin-responsive genes is, perhaps, incidental.

General comment

The manuscript lacks methodological detail in describing the hplc experiments, particularly around the amount of sample injected and does not include all relevant hplc runs.

Specific criticisms

NMR

Individual resonances should be assigned to specific phosphates, so that the effect of L-arg N can be more clearly elaborated. Does L-arg N generate additional resonances for other IP5 isomers?

The data shown for the chiral shift experiments do not disallow the possibility that the IP5 3-OH added has another (perhaps, lower) inositol phosphate impurity: no hplc trace is provided for the added IP5 3-OH.

The resonances arising (in the presence of the chiral shift reagent) from addition of IP5 3-OH is rather less than 50% of the assumed IP5 1-OH resonance , even though there is 50% more IP5 3-OH added than IP5 1-OH. This result is not wholly consistent with the interpretation of the veracity of the chiral shift reagent.

Fig. 2C should show traces for InsP5 1-OH and for InsP5 3-OH in the absence of XopH product, and in presence of the chiral shift reagent.

With the recombinant protein it would be straightforward to generate enough IP5 from IP6 (there are no other products) to assign the resonances in the 31P spectra to individual phosphates by the two-dimensional NMR techniques described by Georg Mayr or Pushpa Murthy.

HPLC

Fig. 2b should include a trace for IP5 3-OH, this will allow proper interpretation of the NMR experiments, by confirming or denying the presence of lower inositol phosphates in the IP5 added to the NMR experiment in Fig. 2c.

It is not shown that IP5 1-OH is the isomer produced in planta by Agrobacterium-delivered XopH expression (Fig. 3). Obviously, the chromatography does not formally distinguish between the 1-OH /3-OH enantiomers, but nor does it distinguish either, or both of them, from IP5 5-OH. These three IP5s co-elute on this column. Strictly, without standards, the chromatography does not properly identify any of the IP5s. (PP-InsP4, not yet found in plants has a similar retention time to IP5s).

Perhaps the simplest test is to show the absence of IP5 5-OH from this 3H-inositol labeled InsP5 peak. This is easily tested by analysis on an Adsorbosphere SAX column. Ideally, some standards should be used. Alternatively, the principal 3H IP5 peak could be desalted, boiled with 1M-HCl for 10 min and run on Part SAX and Adsorbosphere SAX columns. If it yields a peak of IP5 2-OH on Partisphere SAX, without a peak of IP5 5-OH on Adsorbosphere SAX (this isomer elutes first on this column), the peak does not contain IP5 5-OH.

Incidentally, IP5 1-OH has been described in plants (Stephens et al 1991 Biochem. J.) so it is possible that plants have a 1-phytase

Extended Data Fig. 4e should show separate traces for IP6, IP5 1-OH and IP5 3-OH without enzyme, and the legend should state the amount of inositol phosphate injected.

Minor points/comments

The experimental details of all assays generating inositol phosphates to identify products are not given. The reader needs to know enzyme and substrate concentrations, volumes, reaction time etc.

The hplc separations all lack a statement of how much inositol phosphate was injected.

The manuscript should cite the original reference identifying COI as a master regulator of JA signaling.

Page 10 line 16, level of inorganic phosphate is mM in plant cells, considerably higher than IP6

Reviewer #3 (Remarks to the Author):

The authors describe the characterization of the type III bacterial effector XopH and determine that the protein has phosphatase activity, phytase activity, and structural similarity to another phlytase (PhyA). The authors show also that the protein has weak tyrosine kinase activity. They further demonstrate that, with phytic acid as the substrate, that the primary product is IP5 with the phosphate removed from the 1 position. XopH also triggers a resistance reaction in plants with the B7 gene for resistance (here, resistance to bacterial spot disease of pepper, which is caused by Xanthomonas vesicatoria). Phytase activity is also required for the elicitation of resistance by XopH, suggesting that a product of the reaction is the direct elicitor of resistance.The authors also express the gene for XopH ectopically in Nicotiana and determine that a variety of phosphorylated derivatives are changed terms of compound profiles include accumulation of IP5. The plants also have alterations in the expression of a number of defense genes. The authors propose that XopH is a new and novel phyase, specifically cleaving the phosphate at the number 1 position and IP6 may have a signaling function in plants. The findings are very interesting and the phytase activity of XopH is well documented. As far as the work goes, the results are solid. At the same time, this reviewer would like to see some additional experiments and modifications to the manuscript. In brief, the finding that XopH has phytase activity does not indicate that the activity is the germane function of the effector in the plant. Critiques are itemized below somewhat in the order of priority:
1. They authors and previous work referenced show that XopH also has tyrosine phosphatase activity. Work by others, perhaps this group also some time ago, have shown that XopH inhibits flagella induced (flg22) immunity, which is mediated by receptor linked kinases. Kinase activity is regulated by phosphorylation. Macho et al demonstrated that HopAO1 has tyrosine phosphatase activity, interacts with EFR and reduces phosphorylation and, by implication, reduces the immunity response. Hops are

type III effectors from another group of plant pathogenic bacteria, namely Pseudomonas syringae pathovars. Now, HopAO1 (and other effectors with phosphatase activity, eg. HopPtoD2) may have phytase activity or may not. This reviewer is unaware of any testing for this activity. Noneth eless, the previous evidence behooves the authors to measure in vivo tyrosine kinase activity in pepper or, at least, Arabidopsis. In the absence of this evidence, the results are circumstantial. The reported better kinetics with IP6 or the prevalence of IP5 product does not really remove much doubt about the necessary activity in vivo. Also, the protein may degrade some other IP signaling molecule, eg IP1,3,5. Others could be attempted and not assumed that IP6 is a stand-in for all substrates. Also, the fact that JA and ET pathways are affected is little proof of what specifically is occurring.

2. The authors should report on the results of injection of IP5 into leaves of Bs7 pepper. The compound may not reach the active site due to solubility or absorption. However, the attempt should be reported.

3. More background on effector phosphatases and XopH biological activity should be included in manuscript Introduction and/or discussion (some suggestions/examples - Macho et al Science, Popov et al MPMI; Espinosa et al Molecular Microbiology).

4. The fact that that alteration of the active site of XopH causes the loss of Bs7 hypersensitivity was shown previously by Potnis et al (your ref 9) and probably should be included in Introduction.

5. Claims of first are usually not warranted in scientific writing, eg "to our knowledge this is the first" as being the first is not a scientific finding and a bit redundant since if it was not then one would expect some comment or reference. Better might be "novel" or "new" finding. Nonetheless, it occurs commonly in literature now and is perhaps up to the editors. Similar for emotionally laden adjectives (in the results section), eg intriguingly, interestingly - perhaps notably would be better choice. (It is the authors task to make it intriguing [interesting, clear, etc] not tell the reader it is.)

Reviewer #4 (Remarks to the Author):

My expertise is in the area of NMR spectroscopy and I was asked by the editor to specifically assess that portion of the work described in the manuscript. There are a number of comments I have that range from relatively minor to perhaps major issues that need to be addressed before I believe this work can be published.

Minor issues:
1. It would be helpful if early in the manuscript the authors showed the stereochemical structure of inositol with the positions labeled. Then it would be helpful if the authors described the number of phosphorus signals that would be observed for the different InsP5 derivatives based on symmetry arguments. This discussion could then discuss why InsP5 [1-OH] and InsP5[3-OH] represent an enantiomeric pair.

2. I do not understand why the chemical shifts of the phosphorus signals in Figure 6a are different from those in Figures 6b and 2c. This difference needs to be explained.

Major issues:
3. It is unacceptable that the authors only analyzed a racemic mixture of the two enantiomers since they have each in pure form. They must also examine an enriched sample (e.g., 2:1 major-to-minor enantiomer) to show (1) that the two sets of peaks in Figure 6a are in fact the result of differentiation of the enantiomers and (2) to be able to assign the resonances to the [1-OH] and [3-OH] isomers. It

is common in the field of chiral NMR differentiation to examine enriched mixtures of the enantiomers when at least one is available in pure form. The spectra for the enriched sample and racemic sample should be provided.

4. Every sample shown in the spectra in Figures 2 and 6 involve the addition of a large excess of the L-arginine that they use as the chiral solvating agent. If so, then I do not understand why the enantiomeric differentiation of the resonances is so different in the various spectra that are provided. In Figure 6a, it appears that four of the five phosphorus resonances are differentiated. But in Figure 6b, it seems as if only two show differentiation, and the extent of differentiation is not the same between the two spectra. It would help to show integrations of the peaks in Figure 6b as well and for the authors to indicate an assignment of the different resonances. Why does the differentiation change from sample to sample? I am not familiar with such an observation in other studies using chiral NMR solvating agents.

5. I am especially troubled by the spectrum at the top of Figure 2c where 45 ug of the [3-OH] is added. The information provided in the manuscript indicates that the concentration of the [1-OH] and [3-OH] isomers ought to be similar in this spectrum, yet the two new peaks in the top most spectrum are quite small (about equal in size to the impurity used as a reference point). The authors need to provide some convincing justification that these two small peaks are in fact the [3-OH] and not something else taking place in the sample.

If these questions can be satisfactorily answered, then it certainly is an interesting method that the authors have developed for distinguishing the two enantiomeric InsP5 derivatives.

Reviewer #5 (Remarks to the Author):

In this manuscript Blüher et al. describe the phytase activity of Xanthomonas T3E XopH protein. The authors ver nicely use an array of biochemical methods and assays to show that this protein selectively cleaves the 1-O-phosphate group of InsP6 yielding InsP5 [1-OH]. Interestingly, among these methods they also established a new approach based on 31P NMR for the discrimination of InsP5 [1-OH] and InsP5 [3-OH].
The work is novel, well rationalized executed and presented while the conclusions are undoubtfully and clearly supported by the obtained results. These discoveries are expected to have a significant impact in the understanding of bacteria caused crop diseases (on the molecular level) as well as in the potential usage of the obtained knowledge for the introduction of novel, more reliable solutions to fight this problem. Other interesting biotechnological applications may also emerge due to the important implication of polyphosphates in various biological pathways.
Here are a few comments/questions:
1) Throughout the manuscript the authors use the abbreviations InsP6, InsP5, etc. (purchased or synthesized compounds) but these practically refer to different structures (fully acidic form or various salts of the polyphosphate). A comment should be provided defining that these abbreviations refer to the Na salts, clearly defining the number of counter cations.
2) In the chemical synthesis of InsP6: "I. Synthesis of protected hexakisphosphate 2:_50.0 mg…" should be "I. Synthesis of protected hexakisphosphate 2: 50.0 mg…" ("2" in bold).
3) In the chemical synthesis of InsP6 and in the section "II. Synthesis of InsP6": "Piperidinium counter ions were exchanged to sodium ions by addition of excess NaI to a MeOH solution of 3." should be "Piperidinium counter ions were exchanged to sodium ions by addition of excess NaI to a MeOH solution of the piperidinium salt of 3.".
4) In the chemical synthesis of InsP6 and in the section "II. Synthesis of InsP6": "After 30 minutes of stirring, the pure sodium salt of 3 precipitated." How many Na+ this salt has? According to the HRMS

spectrum the obtained salt is the disodium one. Is this correct? Only two Na+? If it is correct then [M]2- has to be changed to [M-2Na+]2- and the authors should carefully check all the solutions concentrations of InsP6 they have prepared and used since commercially available InsP6 is usually the dodecasodium salt, therefore, it has a different molecular weight than the synthesized one.
5) Regarding 31P NMR spectra: It is puzzling to see quite large differences of chemical shifts in Figure 2c and Extended Figure 6a/b for the same P nucleus. Could the authors provide an explanation for these differences?

In total, I have found the work really interesting and I believe that is suitable for publication in Nature Communications regarding novelty, realization and importance of the results. Thus, I stongly recommend acceptance of this manuscript after the authors address the comments given above.

Alexandros E. Koumbis
Associate Professor
Laboratory of Organic Chemistry
Chemistry Department
Aristotle University of Thessaloniki
54124 Thessaloniki
GREECE

In this manuscript Blüher et al. describe the phytase activity of *Xanthomonas* T3E XopH protein. The authors ver nicely use an array of biochemical methods and assays to show that this protein selectively cleaves the 1-O-phosphate group of $InsP_6$ yielding $InsP_5$ [1-OH]. Interestingly, among these methods they also established a new approach based on ^{31}P NMR for the discrimination of $InsP_5$ [1-OH] and $InsP_5$ [3-OH].

The work is novel, well rationalized executed and presented while the conclusions are undoubtfully and clearly supported by the obtained results. These discoveries are expected to have a significant impact in the understanding of bacteria caused crop diseases (on the molecular level) as well as in the potential usage of the obtained knowledge for the introduction of novel, more reliable solutions to fight this problem. Other interesting biotechnological applications may also emerge due to the important implication of polyphosphates in various biological pathways.

Here are a few comments/questions:

1) Throughout the manuscript the authors use the abbreviations $InsP_6$, $InsP_5$, etc. (purchased or synthesized compounds) but these practically refer to different structures (fully acidic form or various salts of the polyphosphate). A comment should be provided defining that these abbreviations refer to the Na salts, clearly defining the number of counter cations.

2) In the chemical synthesis of $InsP_6$: "*I. Synthesis of protected hexakisphosphate 2:*_50.0 mg…" should be ""*I. Synthesis of protected hexakisphosphate* ***2****:* 50.0 mg…".

3) In the chemical synthesis of $InsP_6$ and in the section "*II. Synthesis of InsP*$_6$": "Piperidinium counter ions were exchanged to sodium ions by addition of excess NaI to a MeOH solution of **3**." should be "Piperidinium counter ions were exchanged to sodium ions by addition of excess NaI to a MeOH solution of the piperidinium salt of **3**.".

4) In the chemical synthesis of $InsP_6$ and in the section "*II. Synthesis of InsP6*": "After 30 minutes of stirring, the pure sodium salt of **3** precipitated." How many Na^+ this salt has? According to the HRMS spectrum the obtained salt is the disodium one. Is this correct? Only two Na^+? If it is correct then $[M]^{2-}$ has to be changed to $[M-2Na^+]^{2-}$ and the authors should carefully check all the solutions concentrations of $InsP_6$ they have prepared and used since commercially available $InsP_6$ is usually the dodecasodium salt, therefore, it has a different molecular weight than the synthesized one.

5) Regarding ^{31}P NMR spectra: It is puzzling to see quite large differences of chemical shifts in Figure 2c and Extended Figure 6a/b for the same P nucleus. Could the authors provide an explanation for these differences?

In total, I have found the work really interesting and I believe that is suitable for publication in Nature Communications regarding novelty, realization and importance of the results. Thus, I stongly recommend acceptance of this manuscript after the authors address the comments given above.

Alexandros E. Koumbis
Associate Professor
Laboratory of Organic Chemistry
Chemistry Department
Aristotle University of Thessaloniki
54124 Thessaloniki
GREECE

Dear Reviewers,

thank you very much for your constructive comments that have helped us to substantially improve the manuscript. Most concerns were addressed experimentally as detailed below. Summarizing the most important issues, we (i) have obtained further experimental evidence that the phytase activity of XopH is indeed the relevant biochemical activity, particularly with respect to its recognition in *Bs7* (resistant) plants; (ii) have obtained data showing that $InsP_5$ [1-OH] is the isomer produced *in planta* (excluding the possibility that we might be seeing $InsP_5$ [3-OH], $InsP_5$ [5-OH] or PP-$InsP_4$ in our plant SAX-HPLC chromatograms), (iii) demonstrate that HopAO1 and XopH have distinct biochemical activities and are unlikely to have similar functions in plants; (iv) have performed NMR analyses (including spiking experiments) to further support the ability of L-arginine amide (L-Arg-N) to allow the discrimination between $InsP_5$ [1-OH] and $InsP_5$ [3-OH] and to address concerns raised by reviewers 2, 4 and 5.

Detailed response to the reviewer's comments:

Reviewer #1 (Remarks to the Author):

Summary: This paper describes the biochemical activity of the Xanthomonas type three effector XopH, previously shown to exhibit dephosphorylation activity. The authors demonstrate that the effector can dephosphorylate phytate, a phosphate storage compound that is also involved in protecting plants from pathogens.

"Effector biology" or the sub field that aims to understand the molecular and biochemical function of T3Es is inherently interesting, important for translational biology and can be exceedingly difficult if interactions between pathogen proteins and host targets are not stable. In this work, the authors employ a novel NMR based approach to define XopH as a 1-phytase. Further, in planta data point towards a potential role for XopH in plant hormone pathways. Here, the authors took two approaches. First, transgenically expressing XopH in N. benthamiana plants resulted in stunted, chlorotic plants. Second, the authors transiently express XopH and measure defense gene expression with and without VIGS silencing of genes involved in hormone signaling. The main criticism here is simply that the biological relevance must be questioned. I do not believe that N. benthamiana is a natural host for this pathogen, the protein is being delivered not by Xanthomonas, but by an

Agrobacterium T-DNA, and the host genes are silenced using VIGS, which would be expected to lead to patchy silencing. Nonetheless, the authors have assembled a nice dataset.

Response: Although *N. benthamiana* is not a natural host of *Xcv*, we recently showed that this is solely due to recognition of a single T3E, XopQ, which triggers plant defense and acts as a host range determinant in *Nicotiana* spp. (Adlung et al. , 2016, Front Plant Sci. 7: 1796). Deletion of *xopQ* turns *Xcv* into an *N. benthamiana* pathogen that grows in the tissue and induces typical disease symptoms. In agreement with the reviewer's suggestion to use pepper, we want to point out that we had carried out transient expression studies in the *Xcv*-resistant pepper cultivar ECW-70R (Figure 1d, e): we show that mutant versions of XopH that lost tyrosine-phosphate phosphatase activity but retained phytase activity still induce the HR (suggesting that the phytase activity is the relevant biological activity that is recognized by the Bs7 resistance protein).

Agrobacterium-mediated transient expression in leaves is an accepted tool for functional T3E studies *in planta*. The main advantage is that effector activities can be analyzed separately from potential redundant effects of other T3Es (*Xcv* translocates more than 30 T3Es!). To follow the reviewer's advice and to avoid relying only on (*Agrobacterium*-delivered) T-DNA-dependent XopH expression, we have now performed infection assays with *Xcv* (and mutant *Xcv* strains) in the natural *Xcv* host pepper (*Capsicum annuum*) and in *N. benthamiana* (see below) to further address the biological activity of XopH.

Our experiments demonstrate that infection by *Xcv* increases $InsP_6$ in the absence of XopH and that XopH causes a robust degradation of $InsP_6$ with a concomitant increase in $InsP_5$. We want to point out that enrichment and visualization of unlabeled inositol polyphosphates from biological samples is very challenging and to our knowledge so far has not been achieved for $InsP_x$ species less phosphorylated than $InsP_6$ for any organism. We are therefore excited about the results, especially regarding the *N. benthamiana* samples where $InsP_5$ can be nicely visualized and are grateful for the reviewer's suggestion. The effect of XopH on $InsP_6$ during natural infection is very clear in both pepper and *N. benthamiana*.

Suggestions/questions:

1. Unfortunately, I did not see the catalytically dead version of XopH included as a control for the N. benthamiana transgenics, perhaps because these experiments were initiated prior to the active domain being defined? Without this control, it is probably an overstatement to say that this phenotype is indicative of involvement in ethylene signaling since many things can lead to stunting and chlorosis.

Response: Indeed, transgenic *N. benthamiana* lines were established before full appreciation of XopH's catalytic residues, and it would take us a long time to generate the respective stable lines. We agree with the reviewer that the chlorotic phenotype observed might be caused by many things and moved the respective figure into the supplement.

2. Would it be possible to do any of the gene expression studies in the XopH expressing transgenic plants. For example, could the authors look at defense gene expression in these plants (we would expect to see high expression of PR1b and PR4), and potentially do VIGS on these plants to bolster the hormone connection? This may not be possible, given their developmental defect, but if so, this would strengthen this part of the paper.

Response: Indeed, preliminary data showed *MYC2* downregulation and an upregulation of *PR1b* and *PR4* (*PI-II* was not tested). However, the absolute values were highly variable between different plants. Therefore, we switched to transient assays in *N. benthamiana* which are much more reliable because the plants are not affected by growth deficiencies due to *xopH* expression. All constructs can be tested on the same plants, even on the same leaf, thus minimizing biological variance. In addition, transient assays allowed the analysis of the inactive XopH version ($XopH_{CH}$) as well as the phytase domain alone ($XopH_{Del77}$), thus demonstrating that the phytase activity of XopH is required for gene induction. VIGS experiments showed that gene induction by XopH is inhibited if the ET but not the JA pathway is affected (Fig. 8d). By contrast, VIGS experiments in the *xopH* transgenics are not promising, because these plants are quite small and sensitive against suboptimal conditions like virus-induced biotic stress.

Minor comments:

I think of the marker genes used in this study (especially PR1b) as general defense related genes. The authors should provide appropriate references if these genes have been conclusively tied to specific plant hormone pathways. Otherwise the text should be revised to reflect the conclusion that transient expression of XopH leads to higher defense related gene expression and that silencing EIN2 blocks the elevated expression of Pi-II.

Response: *PR1b*, *PR4* and *PI-II* are described as JA-responsive and often used as marker genes (we now cite two studies in solanaceous plants). Of course, this does not mean that JA is the only stimulus that enhances expression of these genes. After all, we used the fact that ET also induces the genes as starting point for further analyses.

Reviewer #2 (Remarks to the Author):

Summary

This reviewer (Charles Brearley) takes the view that the manuscript makes two notable discoveries, which, criticisms addressed, merit publication of the work in this journal.

The discoveries are:

XopH, a Type III effector, encodes a novel phytase (proven), but further confirmation of the identity of the IP5 product is needed. The character of this phytase appears highly novel.

Chiral shift reagents allow discrimination of enantiomers of inositol phosphate (pentakisphosphate). This requires further elaboration. Response see below

A third discovery: that the phytase has effect on inositol phosphate metabolism when introduced via Agrobacterium into plants, is proven. However, the identity of the inositol phosphate generated is undefined. It is likely that any phytase introduced by Agrobacterium would have effect on inositol phosphate metabolism. That expression of a phytase has effect on jasmonate- or auxin-responsive genes is, perhaps, incidental. Response see below

General comment

The manuscript lacks methodological detail in describing the hplc experiments, particularly around the amount of sample injected and does not include all relevant hplc runs.

Response: We have added detailed information about the protocols and amount of samples injected.

Specific criticisms

NMR

Individual resonances should be assigned to specific phosphates, so that the effect of L-arg N can be more clearly elaborated.

Response: The experiment is designed in a way that additional resonances appearing in the presence of L-Arg-N clearly permit identification of the biologically relevant isomer. To do so, no assignment of the resonances is required, since it is not relevant which resonance displays the most pronounced shift. While the assignment could be achieved by P-H correlation spectroscopy, the presence of a large excess of L-Arg-N that is required for enantiomer designation will bury the relevant signals under its aliphatic resonances (aside from the buffer used in water). Therefore, to conduct the requested experiment, a deuterated L-Arg-N would need to be synthesized in a multistep, very expensive synthesis, as the compound is not commercially available. We think that to follow up on this would be beyond the scope of the current manuscript.

Does L-arg N generate additional resonances for other IP5 isomers?

Response: Such experiments would not help to address our primary question, i.e. to solve the enantiomer identity of the XopH/$InsP_6$ reaction product. We can exclude all other $InsP_5$ isomers including the [4/6-OH] enantiomers as XopH/$InsP_6$ reaction products based on our PAGE and IC experiments. However, we have looked at discrimination of the 4/6 enantiomers of $InsP_5$. So far, we were not able to achieve a peak separation.

However, our manuscript contains the first-proof-of-principle that discriminating inositol phosphate enantiomers using a chiral solvating agent is possible. In this paper, we precisely answer the pertinent question regarding the isomer identity produced by XopH.

We believe that our combined efforts (PAGE, IC and NMR experiments using spiking with chemically pure isomers of known enantiomer identity) provide all information required regarding the stereo-isomeric identity of the inositol phosphate of interest in this study.

The data shown for the chiral shift experiments do not disallow the possibility that the IP5 3-OH added has another (perhaps, lower) inositol phosphate impurity: no hplc trace is provided for the added IP5 3-OH.

Response: The reviewer probably refers to the NMR experiments. The NMR experiments themselves would enable the identification of other and also lower inositol phosphate impurities. To satisfy the reviewer´s concern, we performed a new series of NMR experiments and have added the resonances of enantiomerically pure $InsP_5$ [3-OH] (see also comments below, all experiments were carried out on a new instrument in Freiburg). We have also added IC traces of $InsP_5$ [3-OH] and all other isomers. While the commercial material sometimes contains small impurities (batch to batch variation; marked with an asterisk in Fig. 5a), we are confident that such minor impurities do not interfere with our analysis.

The resonances arising (in the presence of the chiral shift reagent) from addition of IP5 3-OH is rather less than 50% of the assumed IP5 1-OH resonance , even though there is 50% more IP5 3-OH added than IP5 1-OH. This result is not wholly consistent with the interpretation of the veracity of the chiral shift reagent.

Response: We would like to point out that the amount of sample we originally assigned was in the 2-digit microgram range after extraction of the compounds from gels. Therefore, the amount of compound available for the NMR analysis was a sophisticated guess (since loss of compound can occur during the extraction). The amounts added were then calculated based on our initial guess. Thus, the discrepancy in the peaks can easily be explained.

All of the experiments described to satisfy the Reviewer's concerns regarding NMR were carried out on a 600 MHz instrument without cryo-probe in Freiburg since after the move of the Jessen lab to Freiburg we lost access to the 500 MHz cryoprobe NMR instrument in Zürich, where we had obtained all data sets for our original submission. While this led to some changes in the experimental design, we hope the reviewers agree that all our original measurements have been validated and all concerns raised by the reviewers have been satisfactorily addressed.

In order to get a more precise dataset, we have omitted the gel purification to remove phosphate. This also enabled us to generate larger amounts of $InsP_5$ using XopH, so that the cryoprobe was not required anymore (sample amounts of 600nmol were now easy to obtain). However, omission of the purification leads to a phosphate signal in the NMR spectra. The phosphate peak can be identified measuring proton coupled spectra as it appears as a singlet, while the inositol bound phosphates appear as doublets (new Figure 5b). Our new spectra unambiguously identify $InsP_5$ [1-OH] as the product of XopH action on $InsP_6$.

Fig. 2C should show traces for InsP5 1-OH and for InsP5 3-OH in the absence of XopH product, and in presence of the chiral shift reagent.

Response: It needs to be pointed out that ^{31}P NMR is extremely sensitive to the matrix (e.g., little differences in pH). Hence, we have conducted the spiking experiments in a way to achieve identical conditions for the sample and additives. Resonance shifts that occur when obtaining spectra for different samples in slightly different matrices are ruled out in our spiking experiments. This is now explained in the text. We have added a separate analysis to compare the shifts of such samples. It is noteworthy that while the absolute chemical shift of the peaks is not identical, their order is (new

Supplemental Figure 6). In this analysis, we used as little as 100 micrograms of each sample to further underline the potential sensitivity of this approach.

With the recombinant protein it would be straightforward to generate enough IP5 from IP6 (there are no other products) to assign the resonances in the 31P spectra to individual phosphates by the two-dimensional NMR techniques described by Georg Mayr or Pushpa Murthy.

Response: Please see also comments above. We think the novel synthesis of deuterated L-Arg-N required for such analyses and the costly and time-consuming NMR analyses would not be justified, as we would not learn more about the isomer identity of the XopH product, which was the goal of this experiment.

HPLC

Fig. 2b should include a trace for IP5 3-OH, this will allow proper interpretation of the NMR experiments, by confirming or denying the presence of lower inositol phosphates in the IP5 added to the NMR experiment in Fig. 2c.

Response: We had performed IC runs for all inositol polyphosphates employed in our study. We did not detect any major impurities for $InsP_5$ [3-OH], in agreement with our NMR data. However, we agree with the reviewer that we have to demonstrate that the commercial $InsP_5$ isomers are of good quality and now included respective IC-traces in the revised Figure 4c. The quality of the compounds can also be judged from the ^{31}P NMR spectra. While there are minor impurities in some batches used, they do not interfere with our NMR analyses and therefore a multistep stereoselective synthesis of commercial material was not conducted.

It is not shown that IP5 1-OH is the isomer produced in planta by Agrobacterium-delivered XopH expression (Fig. 3). Obviously, the chromatography does not formally distinguish between the 1-OH /3-OH enantiomers, but nor does it distinguish either, or both of them, from IP5 5-OH. These three IP5s co-elute on this column. Strictly, without standards, the chromatography does not properly identify any of the IP5s. (PP-InsP4, not yet found in plants has a similar retention time to IP5s).

Perhaps the simplest test is to show the absence of IP5 5-OH from this 3H-inositol labeled InsP5 peak. This is easily tested by analysis on an Adsorbosphere SAX column. Ideally, some standards should be used. Alternatively, the principal 3H IP5 peak could be desalted, boiled with 1M-HCl for 10 min and run on Part SAX and Adsorbosphere SAX columns. If it yields a peak of IP5 2-OH on

Partisphere SAX, without a peak of IP5 5-OH on Adsorbosphere SAX (this isomer elutes first on this column), the peak does not contain IP5 5-OH.

Response: We agree that in our first submission, we could formally not be sure about the enantiomer identity of the XopH-dependent InsP5 isomer in planta. While we believed it to be likely that also *in planta* XopH executes 1-phytase activity, we were not able to exclude the possibility that an unknown enzymatic activity converts the resulting $InsP_5$ [1-OH] into $InsP_5$ [3-OH], $InsP_5$ [5-OH] or PP-$InsP_4$. To address this concern we followed the reviewer's suggestion to desalt the principal $InsP_5$ peak and then carried out a series of experiments with the purified inositol phosphate species that should convince the reviewer that $InsP_5$ [3-OH], $InsP_5$ [5-OH] and PP-$InsP_4$ can be excluded as the XopH-dependent $InsP_5$ isomer *in planta* and that the product likely represents the $InsP_5$ [1-OH] isomer. In short, we have done the following experiments:

I) To exclude PP-$InsP_4$: We have taken advantage of the published yeast *ipk1* mutant phenotype, i.e. lack of $InsP_6$ and high accumulation of PP-$InsP_4$. As shown in the Figure included here (see below), our HPLC system can nicely discriminate between $InsP_{5c}$ [1-OH/3-OH] and PP-$InsP_4$. These experiments include a spiking experiment where we observed peak separation of XopH-dependent $InsP_{5c}$ from tobacco and yeast *ipk1*-dependent PP-$InsP_4$ in a single HPLC run. We do not think that these data are relevant for the understanding of our experiments but we can include them into the manuscript if considered important.

II) To exclude $InsP_5$ [3-OH/5-OH]: We have purified the $InsP_{5c}$ peak from [^{3}H]-inositol-labeled XopH expressing *N. benthamiana* plants and showed by a series of digestion experiments using recombinant XopH coupled with HPLC analyses that the XopH-dependent product *in planta* cannot represent $InsP_5$ [3-OH] or $InsP_5$ [5-OH] (shown in Figure 7b). We are confident that these data provide overwhelming evidence that $InsP_5$ [1-OH] is the product generated by XopH also *in planta*.

Incidentally, IP5 1-OH has been described in plants (Stephens et al 1991 Biochem. J.) so it is possible that plants have a 1-phytase

Response: The Stephens et al. paper does not claim to be able to distinguish between $InsP_5$ [1-OH] and [3-OH]. Instead, they leave enantiomer identity unaddressed by speaking of D- and/or L-myo-[3H]inositol 1,2,4,5,6-pentakisphosphate...).

We are not sure if plants lack a 1-phytase but our data suggest that none is expressed in larger amounts in adult *N. benthamiana* leaves under the used conditions.

Extended Data Fig. 4e should show separate traces for IP6, IP5 1-OH and IP5 3-OH without enzyme, and the legend should state the amount of inositol phosphate injected.

Response: See above.

Minor points/comments

The experimental details of all assays generating inositol phosphates to identify products are not given. The reader needs to know enzyme and substrate concentrations, volumes, reaction time etc.

The hplc separations all lack a statement of how much inositol phosphate was injected.

Response: This is now taken care of.

The manuscript should cite the original reference identifying COI as a master regulator of JA signaling.

Response: Agreed, the original reference has been added.

Page 10 line 16, level of inorganic phosphate is mM in plant cells, considerably higher than IP6

Response: We find it unlikely that XopH's main function is to improve phosphate availability for the bacteria. However, we cannot exclude that possibility since we know very little about both concentrations and compartmentalization of phosphate and inositol polyphosphates during bacterial infection. For instance, external factors can change cytoplasmic phosphate as e.g. shown in work from Loughman and colleagues in 1989 ("Observations on the cytoplasmic and vacuolar orthophosphate pools in leaf tissues using in vivo 31P spectroscopy", FEBS Lett. 242, 279-284). The authors found 6-fold reduction of cytoplasmic phosphate (from 1.2 mM to 0.2 mM) in maize leaf discs after incubation with 5 mM of the readily phosphorylated glucose analogue D-mannose. A survey from Hadi Alkarawi & Zotz, 2014 ("Phytic acid in green leaves", Plant Biol 16:697-701) suggests that phytic acid concentration in leaves of higher plants is around 2-3 mg /g fresh weight (i.e. approx. 3-4 mM).

Reviewer #3 (Remarks to the Author):

The authors describe the characterization of the type III bacterial effector XopH and determine that the protein has phosphatase activity, phytase activity, and structural similarity to another phlytase (PhyA). The authors show also that the protein has weak tyrosine kinase activity. They further demonstrate that, with phytic acid as the substrate, that the primary product is IP5 with the phosphate removed from the 1 position. XopH also triggers a resistance reaction in plants with the B7 gene for resistance (here, resistance to bacterial spot disease of pepper, which is caused by Xanthomonas vesicatoria). Phytase activity is also required for the elicitation of resistance by XopH, suggesting that a product of the reaction is the direct elicitor of resistance. The authors also express the gene for XopH ectopically in Nicotiana and determine that a variety of phosphorylated derivatives are changed terms of compound profiles include accumulation of IP5. The plants also have alterations in the expression of a number of defense genes. The authors propose that XopH is a new and novel phyase, specifically cleaving the phosphate at the number 1 position and IP6 may have a signaling function in plants. The findings are very interesting and the phytase activity of XopH is well documented. As far as the work goes, the results are solid. At the same time, this reviewer would like to see some additional experiments and modifications to the manuscript. In brief, the finding that XopH has phytase activity does not indicate that the activity is the germane function of the effector in the plant. Critiques are itemized below somewhat in the order of priority:

1. The authors and previous work referenced show that XopH also has tyrosine phosphatase activity. Work by others, perhaps this group also some time ago, have shown that XopH inhibits flagella induced (flg22) immunity, which is mediated by receptor linked kinases. Kinase activity is regulated by phosphorylation. Macho et al demonstrated that HopAO1 has tyrosine phosphatase activity, interacts with EFR and reduces phosphorylation and, by implication, reduces the immunity response. Hops are type III effectors from another group of plant pathogenic bacteria, namely Pseudomonas syringae pathovars. Now, HopAO1 (and other effectors with phosphatase activity, eg. HopPtoD2) may have phytase activity or may not. This reviewer is unaware of any testing for this activity. Nonetheless, the previous evidence behooves the authors to measure in vivo tyrosine kinase activity in pepper or, at least, Arabidopsis. In the absence of this evidence, the results are circumstantial. The reported better kinetics with IP6 or the prevalence of IP5 product does not really remove much doubt about the necessary activity in vivo. Also, the protein may degrade some other IP signaling molecule, eg IP1,3,5. Others could be attempted and not assumed that IP6 is a stand-in for all substrates. Also, the fact that JA and ET pathways are affected is little proof of what specifically is occurring.

Response: We agree with the reviewer that it is difficult to demonstrate that a biochemical activity of a given enzyme is not circumstantial but is of functional relevance. As detailed above, we have shown that XopH not only functions as a 1-phytase *in vitro* and when expressed from an *Agrobacterium*-delivered T-DNA, but also during natural infection with *Xanthomonas* of both *N. benthamiana* and pepper. Please note that for the recognition of XopH by the product of the *Bs7* resistance gene in the resistant pepper cultivar ECW-70R the phytase activity, and not the phosphatase activity, is needed (Fig. 1d). The same is true for the gene induction in *N. benthamina* (Fig. 8a).

We think the reviewer's recommendation to measure *in vivo* tyrosine kinase activity in pepper or, at least, in Arabidopsis is difficult to carry out. Nonetheless, we tried to address the reviewer's point by two different experimental approaches:

I) We generated (and validated) XopH fusions with nuclear export (NES) and nuclear localization (NLS) signals and tested their ability to trigger the HR in the natural host pepper. The rationale behind this: If XopH dephosphorylated a (phosphorylated) Tyr residue in EFR or in any other membrane-resident PRR (as has been described for HopAO1) or in any other larger protein (complex) that cannot easily diffuse in and out of the nucleus, the localization of XopH should be important. But we found the opposite: a strong HR response independent of whether XopH is present in the nucleus or in the cytoplasm (our new Fig. 3a-c). This strongly suggests that the relevant substrate of XopH is likely a small protein or a small molecule such as inositol polyphosphate that can freely diffuse into and out of the nucleus. We also confirmed by HPLC analyses of ^{3}H-inositol labeled *N. benthamiana* plants transiently expressing respective XopH derivatives that XopH converted endogenous $InsP_6$ into $InsP_5$ independent of whether it was expressed in the nucleus or in the cytoplasm (new Fig. 3d).

II) The reviewer's point encouraged us to investigate the biochemical activity of HopAO1 from *P. syringae*. We generated recombinant protein and found indeed phytase activity *in vitro*. However, in contrast to XopH, HopAO1 degrades $InsP_6$ to something smaller than $InsP_3$ as revealed by PAGE analyses (new Supplemental Figure 10).

At this point, we do not want to question the work presented in Cyril Zipfel's Science paper (Macho et al.). To our knowledge, *in vivo* tyrosine phosphatase activity was not shown in their work, i.e. there is no evidence that endogenous EFR protein is less phosphorylated in presence of HopAO1. In fact, tyrosine phosphatase activity was only shown *in vitro* using recombinant HopAO1 protein.

2. The authors should report on the results of injection of IP5 into leaves of Bs7 pepper. The compound may not reach the active site due to solubility or absorption. However, the attempt should be reported.

Response: $InsP_5$ is highly charged (depending on pH about -5 to -7) and does not cross biological membranes. To make inositol polyphosphates membrane permeable, sophisticated chemical precursors have to be synthesized. The Jessen lab has worked several years to develop a protocol for the chemical synthesis of membrane-permeable precursors for a specific $InsP_7$ isomer (Pavlovic et al., 2015, Angew Chem Int Ed Engl) that works in mammalian cells. It would be similarly difficult in the case of $InsP_5$ [1-OH] from a chemical perspective. We think that the suggested experiment is beyond the scope of this paper but represents an interesting direction for future work.

3. More background on effector phosphatases and XopH biological activity should be included in manuscript Introduction and/or discussion (some suggestions/examples - Macho et al Science, Popov et al MPMI; Espinosa et al Molecular Microbiology).

Response: We mention these studies in the revised version of the MS and discuss the Macho et al. Science paper in more detail. We want to thank the reviewer for the suggestion.

4. The fact that that alteration of the active site of XopH causes the loss of Bs7 hypersensitivity was shown previously by Potnis et al (your ref 9) and probably should be included in Introduction.

Response: Done.

5. Claims of first are usually not warranted in scientific writing, eg "to our knowledge this is the first" as being the first is not a scientific finding and a bit redundant since if it was not then one would expect some comment or reference. Better might be "novel" or "new" finding. Nonetheless, it occurs commonly in literature now and is perhaps up to the editors. Similar for emotionally laden adjectives (in the results section), eg intriguingly, interestingly - perhaps notably would be better choice. (It is the authors task to make it intriguing [interesting, clear, etc] not tell the reader it is.)

Response: Point taken.

Reviewer #4 (Remarks to the Author):

My expertise is in the area of NMR spectroscopy and I was asked by the editor to specifically assess that portion of the work described in the manuscript. There are a number of comments I have that range from relatively minor to perhaps major issues that need to be addressed before I believe this work can be published.

Minor issues:

1. It would be helpful if early in the manuscript the authors showed the stereochemical structure of inositol with the positions labeled. Then it would be helpful if the authors described the number of phosphorus signals that would be observed for the different InsP5 derivatives based on symmetry arguments. This discussion could then discuss why InsP5 [1-OH] and InsP5[3-OH] represent an enantiomeric pair.

Response: We thank the reviewer for these suggestions and have included the requested explanations (new Figures 4a and S2). This section also allowed us to discuss the expected multiplicity in proton-coupled spectra of phosphate vs. phosphate esters of inositol. This special feature is now used to assign the enantiomers directly from a digest of $InsP_6$ by XopH so that the purification step by gel electrophoresis was avoided (as explained above, this was necessary to obtain larger amounts of material for the analysis on a 600 MHz NMR without cryoprobe).

2. I do not understand why the chemical shifts of the phosphorus signals in Figure 6a are different from those in Figures 6b and 2c. This difference needs to be explained.

Response: This was caused by a matrix effect (gel purified and extracted vs. not-gel purified). Since the spectra have now been removed, a detailed discussion has not been inserted. The new spectra are discussed in detail, specifically pointing out the sensitivity of ^{31}P chemical shifts to matrix effects (such as pH), which requires spiking experiments. This sensitivity is well-documented in the literature and has actually been used to measure local pH changes. We included a citation (Moon, Richards, JBC, 248, 7276-7278, 1973)

Major issues:

3. It is unacceptable that the authors only analyzed a racemic mixture of the two enantiomers since they have each in pure form. They must also examine an enriched sample (e.g., 2:1 major-to-minor enantiomer) to show (1) that the two sets of peaks in Figure 6a are in fact the result of differentiation of the enantiomers and (2) to be able to assign the resonances to the [1-OH] and [3-OH] isomers. It is common in the field of chiral NMR differentiation to examine enriched mixtures of the enantiomers when at least one is available in pure form. The spectra for the enriched sample and racemic sample should be provided.

Response: We have done experiments accordingly (see new Figure 5a). The ratios chosen were 1:1.5, 1:1 and 1.5:1. All resonances have been assigned in the spectra to the individual enantiomers (A/B).

Also a spectrum is now shown of a 1.5:1 mixture in the absence of L-Arg-N in buffer. Addition of L-Arg-N leads to large changes in chemical shift and separation of the individual signals.

4. Every sample shown in the spectra in Figures 2 and 6 involve the addition of a large excess of the L-arginine that they use as the chiral solvating agent. If so, then I do not understand why the enantiomeric differentiation of the resonances is so different in the various spectra that are provided. In Figure 6a, it appears that four of the five phosphorus resonances are differentiated. But in Figure 6b, it seems as if only two show differentiation, and the extent of differentiation is not the same between the two spectra. It would help to show integrations of the peaks in Figure 6b as well and for the authors to indicate an assignment of the different resonances. Why does the differentiation change from sample to sample? I am not familiar with such an observation in other studies using chiral NMR solvating agents.

Response: As explained above, chemical shifts of free phosphates are massively depending on pH (and counterions). With our initial setup it was impossible to maintain the same conditions for different experiments, as we performed extractions from digests from PAGE (down to estimated 20 micrograms, which is usually not easily resolved by NMR). Therefore, matrix variations occurred (especially, when compared to non-extracted material). This was the reason for conducting spiking experiments. While the conclusions of our first set of data are still valid, we opted to repeat the experiments with larger sample amounts without PAGE purification. This helps to minimize deviations. Since deviations in the matrix still occur, it is mandatory to conduct spiking experiments, as we did. We are confident that the spectra now give conclusive evidence of the identity of the XopH digest product (see also response to reviewer 2).

5. I am especially troubled by the spectrum at the top of Figure 2c where 45 ug of the [3-OH] is added. The information provided in the manuscript indicates that the concentration of the [1-OH] and [3-OH] isomers ought to be similar in this spectrum, yet the two new peaks in the top most spectrum are quite small (about equal in size to the impurity used as a reference point). The authors need to provide some convincing justification that these two small peaks are in fact the [3-OH] and not something else taking place in the sample.

Response: We agree with the referee that the spectrum should be improved. We would like to point out again that this experiment was conducted with an estimated amount of material (ca. 20 micrograms), which is very little for an NMR experiment. Integration was not conducted due to the significant noise obtained in that experiment. For conclusive evidence, we have repeated all experiments on a larger scale (600 nmol) without removing the phosphate (see response to reviewer

2). Only the addition of $InsP_5$ [1-OH] does not cause the appearance of additional peaks in the spiking experiments, while $InsP_5$ [3-OH] in the presence of L-Arg-N causes the appearance of new resonances, also at different ratios (new Fig. 5b-d). These resonances have been assigned to the respective enantiomers. We are confident that these experiments now unambiguously establish L-Arg-N as a chiral solvating agent for $InsP_5$ enantiomers.

If these questions can be satisfactorily answered, then it certainly is an interesting method that the authors have developed for distinguishing the two enantiomeric InsP5 derivatives.

Reviewer #5 (Remarks to the Author):

In this manuscript Blüher et al. describe the phytase activity of Xanthomonas T3E XopH protein. The authors ver nicely use an array of biochemical methods and assays to show that this protein selectively cleaves the 1-O-phosphate group of InsP6 yielding InsP5 [1-OH]. Interestingly, among these methods they also established a new approach based on 31P NMR for the discrimination of InsP5 [1-OH] and InsP5 [3-OH].
The work is novel, well rationalized executed and presented while the conclusions are undoubtfully and clearly supported by the obtained results. These discoveries are expected to have a significant impact in the understanding of bacteria caused crop diseases (on the molecular level) as well as in the potential usage of the obtained knowledge for the introduction of novel, more reliable solutions to fight this problem. Other interesting biotechnological applications may also emerge due to the important implication of polyphosphates in various biological pathways.
Here are a few comments/questions:
1) Throughout the manuscript the authors use the abbreviations InsP6, InsP5, etc. (purchased or synthesized compounds) but these practically refer to different structures (fully acidic form or various salts of the polyphosphate). A comment should be provided defining that these abbreviations refer to the Na salts, clearly defining the number of counter cations.

Response: This information has been added.

2) In the chemical synthesis of InsP6: "I. Synthesis of protected hexakisphosphate 2:_50.0 mg…" should be "I. Synthesis of protected hexakisphosphate 2: 50.0 mg…" ("2" in bold).

Response: Changed accordingly.

3) In the chemical synthesis of InsP6 and in the section "II. Synthesis of InsP6": "Piperidinium counter ions were exchanged to sodium ions by addition of excess NaI to a MeOH solution of 3." should be "Piperidinium counter ions were exchanged to sodium ions by addition of excess NaI to a MeOH solution of the piperidinium salt of 3.".

Response: Changed accordingly.

4) In the chemical synthesis of InsP6 and in the section "II. Synthesis of InsP6": "After 30 minutes of stirring, the pure sodium salt of 3 precipitated." How many Na+ this salt has? According to the HRMS spectrum the obtained salt is the disodium one. Is this correct? Only two Na+? If it is correct then [M]2- has to be changed to [M-2Na+]2- and the authors should carefully check all the solutions concentrations of InsP6 they have prepared and used since commercially available InsP6 is usually the dodecasodium salt, therefore, it has a different molecular weight than the synthesized one.

Response: Synthetic $InsP_6$ was obtained as the 12Na+ salt. This can simply be judged by the full disappearance of the piperidinium counterions in the ^1H NMR of the precipitate (a comment has been added in the methods section). Thus, a full exchange occurred. In addition, no NaI excess was observed in the product (by IC, monitoring I-). It is common that during the ionization process in MS ion exchange occurs (usually by protonation, as H^+ and Na^+ in water are in dynamic equilibrium), so that different mixtures of ions can be observed. In the methods section, we calculate the mass for the protonated $InsP_6$ with 2 negative charges remaining on phosphate groups as M2-: $C_6H_{16}O_{24}P_6$, to indicate that there is no sodium attached, but that it is in a protonated form. This mass is observed as main peak in the mass spectrum. The detector of the hr MS device would not resolve a potential ion with 12 negative charges. In conclusion, MS is not a suitable method to characterize the amount and identity of counterions originally attached to the molecule.

5) Regarding 31P NMR spectra: It is puzzling to see quite large differences of chemical shifts in Figure 2c and Extended Figure 6a/b for the same P nucleus. Could the authors provide an explanation for these differences?

Response: As explained above (responses to reviewers 2 and 4), chemical shifts of free phosphates are massively depending on pH (and counterions, buffers). With our initial setup it was impossible to maintain the same conditions for different experiments, as we performed extractions from digests from PAGE (down to estimated 20 micrograms, which is usually not easily resolved by NMR). Therefore, the variations occurred (especially, when compared to non-extracted material).

While the conclusions of our first set of data are still valid, we have repeated the experiments with larger sample amounts without PAGE purification (to minimize deviations) and conducted spiking experiments. We are confident that the provided spectra (Fig. 5) now give conclusive evidence of the identity of the XopH digest product.

In total, I have found the work really interesting and I believe that is suitable for publication in Nature Communications regarding novelty, realization and importance of the results. Thus, I stongly recommend acceptance of this manuscript after the authors address the comments given above.

Response: We appreciate the reviewer’s positive feedback on our work.

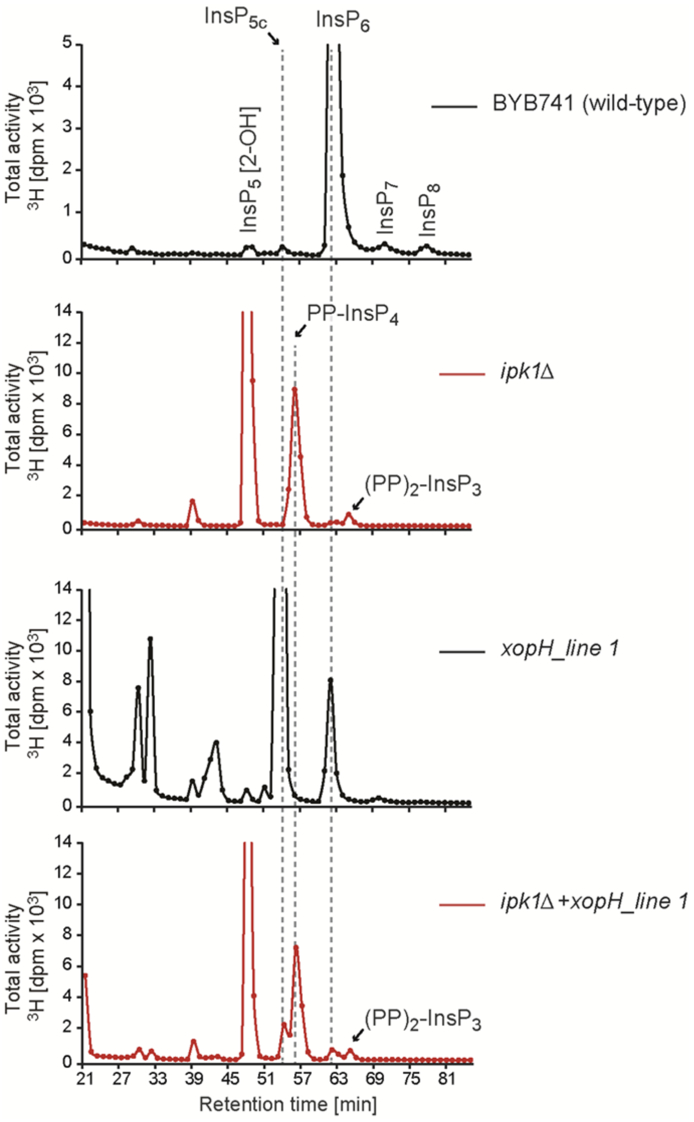

Editorial Note: The top panel in this figure incorrectly iindicated BYB741 as the wild type yeast strain in the response-to-reviewer file. The label should have read EGY48 (wild-type).

Figure: Separation of PP-InsP$_4$ and InsP$_{5c}$ by SAX-HPLC.

Indicated *yeast* strains and *xopH-expressing* transgenic *N. benthamiana* seedlings were labeled with [^{3}H]-*myo*-inositol (see methods). Panels depict HPLC analyses of extracts from indicated genotypes. Based on published chromatographic mobilities, InsP$_{5c}$ represents InsP$_5$ [1/3-OH]. A robust signal of PP-InsP$_4$ peak is detectable in yeast *ipk1Δ* as indicated (York D et al., Science 1999 and Sweetman et al., Biochem J 2006).

Reviewers' comments:

Reviewer #1 (Remarks to the Author):

The authors have addressed my concerns or replied with reasonable explanations regarding why suggested experiments were not feasible. Specifically, inclusion of additional assays within the pepper host improve this manuscript.

Reviewer #2 (Remarks to the Author):

This reviewer (Charles Brearley) will restrict himself solely to the points raised in his review of the original manuscript.

In this revised manuscript the authors have made considerable efforts to clarify their use of chiral shift reagents to assign identity to the IP5 product generated in vitro with recombinant XopH, this aspect of the work is much more convincing for the revision.

The authors have also provided data indicating the quality of the commercial IP5s used in the manuscript and have provided much of the detail of methodology lacking from the original manuscript.

With regards to identification of IP5 species present in plants infected with Agrobacterium bearing XopH, the authors have performed additional experiments: they have recovered IP5 and IP6 from 3H Inositol-labelled plants and shown Fig. 7 that when this 3H IP6 is presented as substrate to recombinant XopH the product runs on the original column in the position of 3H IP5c. This still does not exclude the possibility that parent plant-derived 3H IP5c peak does not contain IP5 5-OH or some IP5 3-OH.

Incidentally, Figure 7b should show the whole chromatograms. This may help show that lower IP products were not detected when plant-derived 3H IP5 was presented to XopH, this would be consistent with the in vitro properties of XopH revealed in Suppl.Fig. 3. viz. the degradation of IP5 3-OH and not IP5 1-OH.

A closer reading of Stephens et al 1991, Page 496 Discussion Paragraph 2, clearly states that the predominant IP5 in the D-and/or L-Ins(1,2,4,5,6)P5 fraction of labelled germinating mung bean is the L-enantiomer ie. D-Ins(2,3,4,5,6)P5 [IP5 1-OH].

In summary, the authors have gone to great lengths to improve the manuscript and it should be published. The authors should be encouraged to make clear for the reader the limitations of the HPLC methods used and to cite Stephens et al 1991.

Reviewer #3 (Remarks to the Author):

The work represents an important new finding regarding type 3 effector function and should be valuable to the scientific community. The authors have presented considerable evidence that, indeed, XopH can function as a 1-phytase and that the activity could represent the critical function of XopH as a virulence factor. The findings are correlative in nature, though strong, and open a new window in the analysis of host/plant pathogen, possibly animal pathogen research.

Reviewer #4 (Remarks to the Author):

The authors have done an excellent job addressing the two minor and three major issues on the NMR parts of their experiment that I raised in my review. This portion of the manuscript satisfactorily supports their assertions about the system they are studying and I would now support publication of this work.

Reviewer #5 (Remarks to the Author):

As I had previously commented, to my opinion, this work is really interesting and suitable for publication in Nature Communications regarding novelty, realization and importance of the results. The presented work was further improved since the authors followed the instructions of the referees. Thus, I recommend acceptance of this manuscript after the authors make the minor corrections given below.

Original Comment 1:
Throughout the manuscript the authors use the abbreviations InsP6, InsP5, etc. (purchased or synthesized compounds) but these practically refer to different structures (fully acidic form or various salts of the polyphosphate). A comment should be provided defining that these abbreviations refer to the Na salts, clearly defining the number of counter cations.
Authors response: This information has been added.
Comment: I could not locate this information either in the main text or in the supporting information.

Original Comment 4:
In the chemical synthesis of InsP6 and in the section "II. Synthesis of InsP6": "After 30 minutes of stirring, the pure sodium salt of 3 precipitated." How many Na+ this salt has? According to the HRMS spectrum the obtained salt is the disodium one. Is this correct? Only two Na+? If it is correct then [M]2- has to be changed to [M-2Na⁺]2<sup>-</sub> and the authors should carefully check all the solutions concentrations of InsP6 they have prepared and used since commercially available InsP6 is usually the dodecasodium salt, therefore, it has a different molecular weight than the synthesized one.
Authors response: Synthetic InsP6 was obtained as the 12Na+ salt. This can simply be judged by the full disappearance of the piperidinium counterions in the 1H NMR of the precipitate (a comment has been added in the methods section). Thus, a full exchange occurred. In addition, no NaI excess was observed in the product (by IC, monitoring I-). It is common that during the ionization process in MS ion exchange occurs (usually by protonation, as H+ and Na+ in water are in dynamic equilibrium), so that different mixtures of ions can be observed. In the methods section, we calculate the mass for the protonated InsP6 with 2 negative charges remaining on phosphate groups as M2-: C6H16O24P6, to indicate that there is no sodium attached, but that it is in a protonated form. This mass is observed as main peak in the mass spectrum. The detector of the hr MS device would not resolve a potential ion with 12 negative charges. In conclusion, MS is not a suitable method to characterize the amount and identity of counterions originally attached to the molecule.
Comment: The ion exchange in MS could be a possible explanation. However, it seems rather strange that all Na+ are exchanged since there are many reports in the literature which give peaks for M-Na or M-2Na in HRMS of various myo-inositol polyphosphates. Questions: a) Have you counted the piperidinium protons in the 1H NMR of InsP6 piperidinium salt? b) Have you observed in HRMS peaks for M-Na or M-2Na (where M = C6H6O24P6Na12)? c) Have you considered elemental analysis in order to overcome the lack of MS suitability to define countercations? In any case, if you have obtained the 12Na+ salt of InsP6 then you have to write [M-12Na⁺+10H⁺]2< sup>-

</sup> instead of [M]2-.

Alexandros E. Koumbis
Professor
Laboratory of Organic Chemistry
Chemistry Department
Aristotle University of Thessaloniki
54124 Thessaloniki
GREECE

<u>Reviewer #1 (Remarks to the Author):</u>

The authors have addressed my concerns or replied with reasonable explanations regarding why suggested experiments were not feasible. Specifically, inclusion of additional assays within the pepper host improve this manuscript.

<u>Reviewer #2 (Remarks to the Author):</u>

This reviewer (Charles Brearley) will restrict himself solely to the points raised in his review of the original manuscript.

In this revised manuscript the authors have made considerable efforts to clarify their use of chiral shift reagents to assign identity to the IP5 product generated in vitro with recombinant XopH, this aspect of the work is much more convincing for the revision.

The authors have also provided data indicating the quality of the commercial IP5s used in the manuscript and have provided much of the detail of methodology lacking from the original manuscript.

With regards to identification of IP5 species present in plants infected with Agrobacterium bearing XopH, the authors have performed additional experiments: they have recovered IP5 and IP6 from 3H Inositol-labelled plants and shown Fig. 7 that when this 3H IP6 is presented as substrate to recombinant XopH the product runs on the original column in the position of 3H IP5c. This still does not exclude the possibility that parent plant-derived 3H IP5c peak does not contain IP5 5-OH or some IP5 3-OH.

Incidentally, Figure 7b should show the whole chromatograms. This may help show that lower IP products were not detected when plant-derived 3H IP5 was presented to XopH, this would be consistent with the in vitro properties of XopH revealed in Suppl.Fig. 3. viz. the degradation of IP5 3-OH and not IP5 1-OH.

<u>Response:</u> The observation that the amount of purified $InsP_{5c}$ (from *xopH*-expressing *N. benthamiana* seedlings as presented in Fig. 7b) remains unaltered when incubated with recombinant XopH, strongly suggests that the XopH-dependent, plant-derived $InsP_{5c}$ largely represents $InsP_5$ [1-OH]. We followed the reviewer's advice and now present the full chromatograms as new Supplementary Figure 8. We hope, the reviewers agree that when compared with the full chromatogram of $InsP_{5c}$ alone, there is no major change in the InsP profile of $InsP_{5c}$ treated with

XopH suggesting that the species indeed largely represents $InsP_5$ [1-OH]. However, we want to point out that our method might not be sensitive enough to exclude the possibility that the endogenous $InsP_{5c}$ peak (underlying the XopH-dependent $InsP_5$ [1-OH]) might contain small amounts of either $InsP_5$ [5-OH] and/or $InsP_5$ [3-OH]. While we think XopH might be useful to follow up on this question, it was our prime interest to identify the *in planta* isomer identity of XopH-dependent $InsP_6$ hydrolysis. To answer the question whether the endogenous plant $InsP_{5c}$ peak contains $InsP_5$ [5-OH] and/or $InsP_5$ [3-OH], certainly a different strategy to obtain sufficient material of this peak (in the absence of XopH expression) is necessary. In addition, the exact identity of XopH-mediated hydrolytic products of $InsP_5$ [5-OH] and $InsP_5$ [3-OH] would be required which in our opinion is beyond the scope of the current study.

A closer reading of Stephens et al 1991, Page 496 Discussion Paragraph 2, clearly states that the predominant IP5 in the D-and/or L-Ins(1,2,4,5,6)P5 fraction of labelled germinating mung bean is the L-enantiomer ie. D-Ins(2,3,4,5,6)P5 [IP5 1-OH].

In summary, the authors have gone to great lengths to improve the manuscript and it should be published. The authors should be encouraged to make clear for the reader the limitations of the HPLC methods used and to cite Stephens et al 1991.

Response: We very much appreciate that the reviewer would like to see our manuscript published and apologize for omitting the Stephens *et al.* study. We overlooked these data because they were presented as "results not shown" in the cited paragraph. We have now included this important paper (and point out its strength to propagate the use of enzyme activities to discriminate InsP enantiomers) and made a comment on the limitation of HPLC analyses (pp. 15 and 9, respectively).

Reviewer #3 (Remarks to the Author):

The work represents an important new finding regarding type 3 effector function and should be valuable to the scientific community. The authors have presented considerable evidence that, indeed, XopH can function as a 1-phytase and that the activity could represent the critical function of XopH as a virulence factor. The findings are correlative in nature, though strong, and open a new window in the analysis of host/plant pathogen, possibly animal pathogen research.

Reviewer #4 (Remarks to the Author):

The authors have done an excellent job addressing the two minor and three major issues on the NMR parts of their experiment that I raised in my review. This portion of the manuscript satisfactorily supports their assertions about the system they are studying and I would now support publication of this work.

Reviewer #5 (Remarks to the Author):

As I had previously commented, to my opinion, this work is really interesting and suitable for publication in Nature Communications regarding novelty, realization and importance of the results. The presented work was further improved since the authors followed the instructions of the referees. Thus, I recommend acceptance of this manuscript after the authors make the minor corrections given below.

Original Comment 1:

Throughout the manuscript the authors use the abbreviations InsP6, InsP5, etc. (purchased or synthesized compounds) but these practically refer to different structures (fully acidic form or various salts of the polyphosphate). A comment should be provided defining that these abbreviations refer to the Na salts, clearly defining the number of counter cations.

Authors response: This information has been added.

Comment: I could not locate this information either in the main text or in the supporting information.

Response: The reviewer might have missed the relevant sentence. The information was indeed provided in the methods section (p. 27): “$InsP_5$ [1-OH] and $InsP_5$ [3-OH] as decasodium salts (1 mg batches, dissolved as a stock solution in 100 µL D_2O) were from Sichem.”

Original Comment 4: In the chemical synthesis of InsP6 and in the section “II. Synthesis of InsP6”: “After 30 minutes of stirring, the pure sodium salt of 3 precipitated.” How many Na+ this salt has? According to the HRMS spectrum the obtained salt is the disodium one. Is this correct? Only two Na+? If it is correct then [M]2- has to be changed to [M-2Na+]2- and the authors should carefully check all the solutions concentrations of InsP6 they have prepared and used since commercially available InsP6 is usually the dodecasodium salt, therefore, it has a different molecular weight than the synthesized one.

Authors response: Synthetic InsP6 was obtained as the 12Na+ salt. This can simply be judged by the full disappearance of the piperidinium counterions in the 1H NMR of the precipitate (a comment has been added in the methods section). Thus, a full exchange occurred. In addition, no NaI excess was

observed in the product (by IC, monitoring I-). It is common that during the ionization process in MS ion exchange occurs (usually by protonation, as H+ and Na+ in water are in dynamic equilibrium), so that different mixtures of ions can be observed. In the methods section, we calculate the mass for the protonated InsP6 with 2 negative charges remaining on phosphate groups as M2-: C6H16O24P6, to indicate that there is no sodium attached, but that it is in a protonated form. This mass is observed as main peak in the mass spectrum. The detector of the hr MS device would not resolve a potential ion with 12 negative charges. In conclusion, MS is not a suitable method to characterize the amount and identity of counterions originally attached to the molecule.

Comment: The ion exchange in MS could be a possible explanation.

Response: We agree with the referee.

However, it seems rather strange that all Na+ are exchanged since there are many reports in the literature which give peaks for M-Na or M-2Na in HRMS of various myo-inositol polyphosphates.

Response: This will depend on the conditions used (e.g. adding excess Na^+ to the solution prior to ionization in ESI). We used only water in the ESI process. However, it does not change the fact that MS is not suitable to determine the number of counterions. If an $InsP_6$ 12Na is dissolved in water as shown in the following scheme, it will act as a base, deprotonate water and generate (dissociated) NaOH. This can happen up to 12 times.

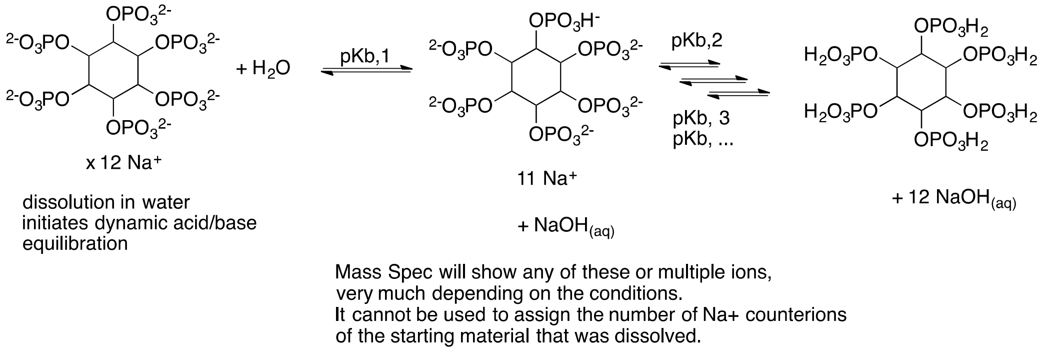

There will be many dynamic equilibria delivering a complex mixture of different protonated states, that will also significantly shift during the ESI process. Mass Spec will not enable the assignment of the number of counterions that were initially put into the system upon dissolution of the salt. We usually focus on the least charged states, i.e. , 1- or 2-. In this case, we observed the protonated form (see spectrum attached).

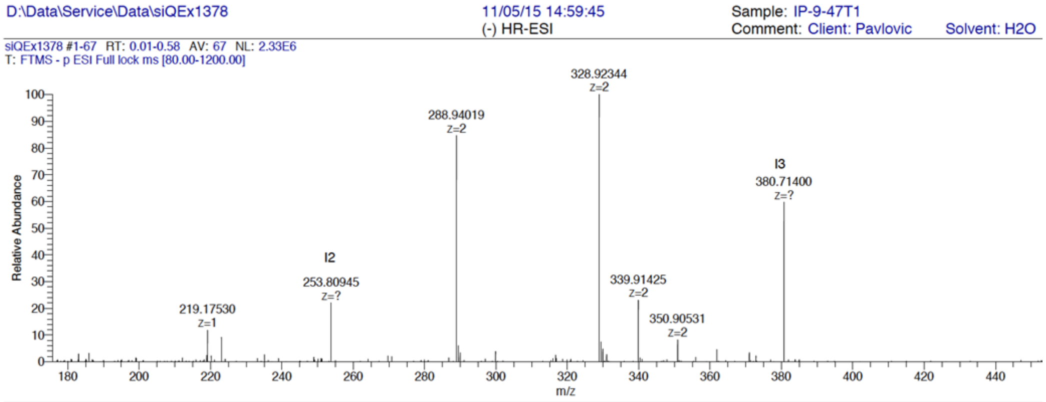

siQEx1378#1-67 RT: 0.01-0.58 AV: 67
T: FTMS - p ESI Full lock ms [80.00-1200.00]
m/z= 277.37777-359.10556

m/z	Intensity	Relative	Charge	Theo. Mass	Delta (ppm)	RDB equiv.	Composition
288.94019	1991299.1	83.55	2.00	288.94024	-0.05	2.0	$C_6H_{15}O_{21}P_5$
328.92344	2383234.0	100.00	2.00	328.92341	0.03	2.0	$C_6H_{16}O_{24}P_6$
339.91425	539330.9	22.63	2.00	339.91438	-0.13	2.0	$C_6H_{15}O_{24}NaP_6$

The most intense peak (which we indicate in the paper) is 328.92344. This corresponds to the protonated form with two negative charges. 339.91425 is the monosodium adduct 2- and 350.90531 is the disodium adduct 2-. There are also forms in which the ionization process leads to hydrolysis of a phosphate (288.94019; However, this species is not relevant, as it cannot be seen in the ^{31}P NMR, it only appears during ionization)

This analysis is not meant to characterize the number of counterions, as it is dynamic in solution. It simply gives evidence of the successful synthesis of $InsP_6$ (no counterions defined; that is why we reference the mass to the protonated state). We derive the number of counterions from the synthesis as shown in the following scheme:

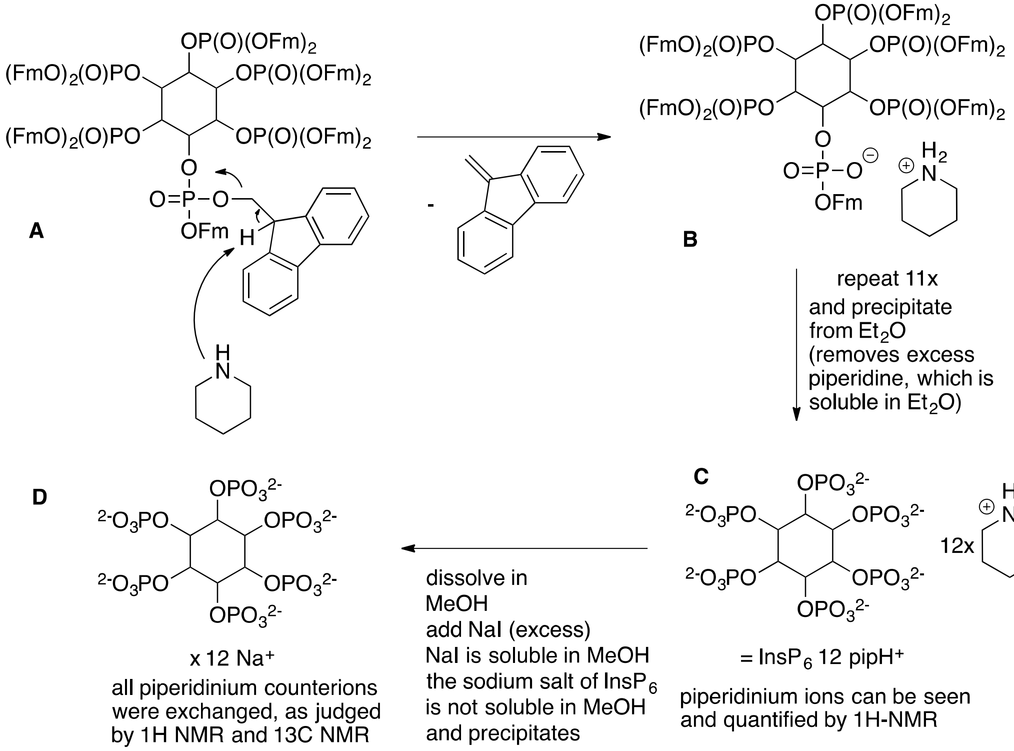

Shown is the deprotection of synthetic material **A** with 12 Fm groups. 1 Fm group is drawn with its chemical structure. Addition of piperidine leads to deprotonation of the acidic proton in the protecting group (following an E1cb mechanism), which leads to elimination of the phosphate in its negatively charged state. In this event, the proton is transferred to piperidine, giving protonated piperdinium ions (pipH+, part **B**). Thus, removal of each Fm group leads to generation of 1 $pipH^+$. Removal of all protecting groups therefore leads to the generation of 12x $pipH^+$ (part **C**). Addition of Et_2O leads to precipitation of this salt (as described in Methods). The ^1H NMR shows the presence of 12 $pipH^+$ - this is also the answer to referee´s question a; integration of the relevant resonances indicates 12 counterions per $InsP_6$ molecule. Thus, also $pipH^+$ does not protonate the $InsP_6$ as pip is soluble in Et_2O and did not precipitate. The 12 $pipH^+$ $InsP_6$ salt is soluble in MeOH, as is NaI. We added excess of NaI to induce precipitation of the sodium salt of $InsP_6$ (which is not soluble in MeOH). The precipitate does not show any $pipH^+$ resonances, therefore, all $pipH^+$ must have been exchanged (this is described in the methods section: "*Piperidinium counter ions were exchanged to sodium ions by addition of excess NaI to a MeOH solution of the piperidinium salt of* ***3****. After 30 minutes of stirring, the pure sodium salt of* ***3*** *precipitated, evidenced by the absence of proton resonances of piperidinium ions in the* ^1H *NMR spectrum. The purity was confirmed by ion chromatography as described in the next section.")*. If all 12 $pipH^+$ were exchanged, and only Na^+ is present as an alternative counterion (added as NaI), the product must contain 12 Na^+ (part **D**). There

was no excess of Na^+ in the product, since this would have a counterion I^-, which was not seen upon analysis of the product by ion chromatography monitoring I^- (also mentioned in the methods section).

Also, as a hypothetical argument, the following scheme gives the relative masses of two different protonated states of $InsP_6$ (one time 12 Na^+, one time 6 Na^+):

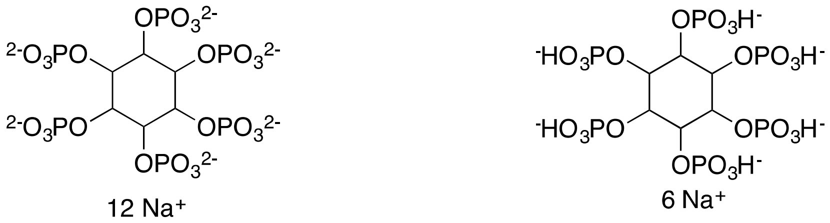

Chemical Formula: $C_6H_6Na_{12}O_{24}P_6$
Molecular Weight: 923.81

Chemical Formula: $C_6H_{12}Na_6O_{24}P_6$
Molecular Weight: 791.92

The form with 6 Na^+ (and 6 H^+) still has 86% of the molecular weight of the form with 12 Na^+. Therefore, even if there was a difference in Na^+ content (which we did not observe when we compared synthetic and commercial material based on IC integration), it would be small and not change any conclusions of the current manuscript. However, comparison of precisely weighed in commercial substances ($InsP_5$) and the digest of synthetic $InsP_6$ analyzed by IC revealed almost identical peak integrals, thereby further proving the identity of the synthetic material as $12Na^+$.

Questions: a) Have you counted the piperidinium protons in the 1H NMR of InsP6 piperidinium salt?

Response: See above.

b) Have you observed in HRMS peaks for M-Na or M-2Na (where M = C6H6O24P6Na12)?

Response: We have indicated the main peak of the spectrum. The obtained MS is shown above. It cannot be used to assign the number of counterions; therefore, reporting all peaks that appear would not provide significant additional information. However, if one adds excess NaI to the solution for ESI analysis, usually higher Na^+ adducts can be seen. In our analysis, we did not add NaI and simply used pure water.

c) Have you considered elemental analysis in order to overcome the lack of MS suitability to define countercations?

Response: This is an interesting suggestion. Combustion analysis will, however, only provide CHNSO composition. Na^+ would not be observable. We are confident that the analysis provided here with regards to $pipH^+$ to Na^+ exchange makes our point clear. We would, however, not like to include this analysis in detail in the manuscript (main aspects of it are in the methods section) as also for commercial materials no such analyses are usually provided. With this analysis, we are following a rationale previously published by us in Nat Commun, 2016, volume: 7, pages: 10622)

In any case, if you have obtained the 12Na+ salt of InsP6 then you have to write [M-12Na++10H+]2- instead of [M]2-.

Response: We were referring to the fully protonated form as reference. We have changed this now accordingly and thank the referee for this suggestion. We hope that this is now clearer.

REVIEWERS' COMMENTS:

Reviewer #5 (Remarks to the Author):

The authors have addressed my concerns and replied with reasonable explanations to my comments. They have also made the appropriate additions to their manuscript.
To my opinion, this work is particularly interesting and definitely suitable for publication in Nature Communications regarding novelty, realization and importance of the results. Thus, I recommend acceptance of this manuscript as it is.

Alexandros E. Koumbis
Professor
Laboratory of Organic Chemistry
Chemistry Department
Aristotle University of Thessaloniki
54124 Thessaloniki
GREECE
